# Estimation of Future Patient Populations Eligible for Radioligand Therapies in the EU and the UK: A Modelling Study

**DOI:** 10.1016/j.lanepe.2026.101733

**Published:** 2026-06-04

**Authors:** Uwe Holzwarth, Roberta Cirillo, Margarida Goulart, Diego Hernandez, Platon Peristeris, Andreas Poschenrieder, Panagiota Papaioannou, Panos Kanavos, Christian la Fougère, Leonhard Schaetz

**Affiliations:** a European Commission, Joint Research Centre, Ispra, Italy; b European Commission, Joint Research Centre, Brussels, Belgium; c WifOR – Macroeconomic Research Institute & Think Tank, Darmstadt, Germany; d RLT Healthcare Systems Readiness & Partnerships, Novartis, Geneva, Switzerland; e RLT Healthcare Systems Readiness & Partnerships, Novartis Pharma AG, Basel, Switzerland; f Department of Health Policy, London School of Economics and Political Science, London, UK; g Nuklearmedizin und Klinische Molekulare Bildgebung, Universitätsklinikum Tübingen, Tübingen, Germany

**Keywords:** Nuclear medicine, Radioligand therapy, Patient numbers, Treatment capacity, Cancer

## Abstract

**Background:**

The eligible patient population for radioligand therapies (RLTs) has the potential to grow rapidly in the coming years. European healthcare systems may be ill-prepared to provide the additional treatment capacity required on top of current workload. To address this challenge, data on the size of the eligible patient pool and the potential utilisation of RLTs are needed.

**Methods:**

The maximum pool of patients theoretically eligible for RLTs over the next decade was estimated for the 27 EU member states (EU-27) using incidence data, and additionally for France, Germany, Italy, Spain (EU-4) and the UK using prevalence data. Conditions for which RLT are currently authorised, potential expansions of existing authorisations to earlier disease stages, and anticipated authorisations for products in development were analysed.

**Findings:**

Between 2025 and 2035 in the EU-27, the patient pool theoretically eligible for RLTs could increase from 11,000 to 13,000 to approximately 130,000–180,000. A scenario based on preliminary utilisation data suggests that, in the EU-4, 17,000–35,000 patients from this pool could be treated in 2030, increasing to approximately 37,000 patients by 2035.

**Interpretation:**

The expected uptake of RLTs in EU healthcare systems over the coming decade may overwhelm available treatment capacity, even in countries with well-developed nuclear medicine infrastructure. Efforts across all EU countries will be required to ensure adequate patient access to RLTs and to minimise health inequalities in cancer care.

**Funding:**

Research by WifOR was funded by Novartis Pharma AG. JRC research was covered by the European Commission’s Research Framework Programmes budget.

**Research in context**

**Evidence before this study**

The rapid adoption of radioligand therapies (RLTs) for neuroendocrine tumours and prostate cancer underscores the growing importance of nuclear medicine in precision oncology. With promising clinical advancements, RLTs for additional cancer types are nearing approval, offering innovative treatment options for an expanding patient population. However, scaling nuclear medicine capacity to meet the increasing demand requires substantial time and resources, raising concerns about potential access limitations. The JRC and WifOR performed literature studies, which were periodically repeated between 2023 and 2025 to catch new data and developments. Targeted literature search was conducted to identify existing evidence on the use of RLTs, their clinical development, and the readiness of healthcare systems to accommodate the increasing demand. The JRC used the search string “(radioligand OR radiopharmaceutical OR radio-pharmaceutical) AND (therapy OR treatment OR therapeutic) AND (“patient number” OR “number of patients”) AND (Europe OR European) AND healthcare” using PubMeD and SCOPUS (first, 13.09.2023). WifOR’s search strategy combined keywords related to radioligand therapy and healthcare system capacity, including: radioligand therapy/radiopharmaceutical therapy/nuclear medicine, cancer/neoplasia/tumor, healthcare system, readiness, infrastructure, capacity, and Europe. The search was performed in PubMed and Google Scholar for peer-reviewed publications (first, 17.04.2023). Grey literature sources were also explored to capture reports and data relevant to healthcare system readiness and infrastructure that may not be published in academic journals. The result was that to date, until 11 February 2026 (cut-off for the present work), no quantitative data exist on how many patients could benefit from RLT treatments that could be used for informed decisions on expanding nuclear medicine services in EU healthcare systems. The present study systematically evaluated authorised RLTs listed by the European Medicines Agency and ongoing phase 2/3 clinical trials registered in the US National Library of Medicine’s database (as of 03 July 2025). By analyzing these sources, we identified clinical indications for which RLTs are currently approved and those likely to receive expanded authorisations or first-time approvals by 2035. For each indication, a secondary targeted search of published peer reviewed articles was performed to retrieve therapy-specific parameters (e.g., indication, eligibility criteria) required to estimate the maximum expected pool size of biologically and clinically eligible patients. Incidence data were retrieved from the European Cancer Information System (covering the entire European Union and its 27 member states), and prevalence data from the Institute for Health Metrics and Evaluation (for Germany, France, Italy, Spain, and the UK), both accessed in 2024.

**Added value of this study**

This study quantifies the expected increase in the pool of patients biologically and clinically eligible for currently available and emerging RLTs across all EU countries and the UK over the next decade. To achieve this, we assumed that new therapies would obtain marketing authorisation according to indicative timelines for their anticipated approval years. We conducted an extensive literature review of relevant biological and clinical data to estimate the proportion of patients meeting authorised clinical indications and those under investigation in clinical trials. It also presents a scenario for the likely uptake of RLTs in the healthcare systems of the EU-4 (Germany, France, Italy, and Spain), based on preliminary utilisation data in literature. These projections may support informed decision-making and planning in European national healthcare systems on how to address the challenges of providing patient access to RLTs at the necessary scale.

**Implications of all the available evidence**

The estimated number of patients in the biologically and clinically eligible patient pool for RLTs is based on anticipated extensions of marketing authorisations to treat earlier in the course of disease and on expected regulatory approvals for new therapeutics. These estimates provide an indication of the magnitude of the challenge national healthcare systems are likely to face. However, the actual number of treated patients will depend on uptake patterns and timelines for RLT adoption by national healthcare systems and is likely to be lower due to factors such as national reimbursement decisions, treatment guidelines, and limitations in adequately trained medical workforce, hospital infrastructure and economic resources. Despite these challenges, we modelled an uptake scenario for RLT in the four largest EU countries (Germany, France, Italy, and Spain), which have well-developed nuclear medicine infrastructure, based on initial utilisation data from high-income countries. The projected patient numbers are substantial enough to warrant immediate consideration by national healthcare decision-makers. Proactive planning is essential to ensure that European cancer patients continue to have access to state-of-the-art treatments, in line with the goals of the European Cancer Mission. The finding that the uptake scenario shows only 30–40% of the patients in the eligible pool receiving treatment should not diminish the commitment to devising strategies that make RLTs accessible to all patients who could benefit clinically and by improved quality of life, particularly where infrastructural and workforce shortages contribute to constraints. This challenge may be even greater in the EU countries where there was no ^177^Lu-RLT availability in 2020.

## Introduction

Radioligand therapy (RLT) is a field of precision oncology that utilises a radionuclide-labelled ligand specific to a molecule overexpressed on the surface of cancer cells, allowing for targeted delivery of cytotoxic radiation to maximise damage to the cancer cells while minimising damage to neighbouring healthy tissues.^1^^,^^2^ The systemic nature of RLT allows for the treatment of metastatic and disseminated disease, whereby patients are selected for therapy using a companion diagnostic compound that allows for quantitative assessment of target expression through imaging such as positron emission tomography/computed tomography (PET/CT).^1^^,^^2^ This theranostic approach ensures the selection of patients who may benefit the most from treatment due to high target expression.^3^

The initial development of radiopharmaceuticals such as [^177^Lu]Lu-DOTA-TATE (^177^Lu-DOTATATE) and, more recently, ^177^Lu-PSMA RLTs was pioneered by a small number of committed clinicians in academic centres in the Netherlands, Germany, Italy, Switzerland, and Australia^4^; and two small enterprises demonstrated the clinical and commercial viability of these therapies.^5^ More recently, pharmaceutical companies have become engaged in RLT development, supporting availability on a global scale.^6^ RLTs are poised to drive a paradigm shift, significantly increasing the role of nuclear medicine in cancer care.^7^

Pivotal trials of ^177^Lu-DOTATATE in patients with somatostatin receptor 2 (SSTR2)-positive advanced, progressive, midgut neuroendocrine tumors (NETs) and [^177^Lu]Lu-PSMA-617 (^177^Lu-PSMA-617) in patients with progressive prostate-specific membrane antigen (PSMA)-positive metastatic castration-resistant prostate cancer (mCRPC) showed that RLTs were generally well-tolerated and associated with improved progression-free survival compared with established therapies.^8^^,^^9^ Furthermore, RLT improved or maintained health-related quality of life (HRQoL) for a longer period for these patients.^10^^,^^11^ With the authorisation of ^177^Lu-DOTATATE by the European Medicines Agency (EMA) and the US Food and Drug Administration (FDA) in 2017 and 2018, respectively,^12^^,^^13^ RLT passed the proof of principle and was integrated into the clinical management of patients with NETs.^14^ While ^177^Lu-DOTATATE is indicated for a relatively small patient population and rare tumor type, the FDA and EMA authorisation of ^177^Lu-PSMA-617 for patients with PSMA-positive mCRPC treated with a prior androgen receptor pathway inhibitor (ARPI) and taxane-based chemotherapy in 2022,^15^, ^16^, ^17^ substantially increased the number of patients that are candidates for RLT.^18^

In 2025, following positive outcomes in the PSMAfore study, the indication for ^177^Lu-PSMA-617 was expanded in the US to include patients with PSMA-positive taxane-naive mCRPC who have been treated with an ARPI and are considered appropriate to delay taxane-based chemotherapy.^16^^,^^18^^,^^19^ Over one hundred phase 2 and 3 clinical trials of RLTs are ongoing, including studies of PSMA- and SSTR-targeted RLT agents in earlier disease stages, as well as additional molecular targets for other cancer types such as haematological malignancies and clear cell renal cell carcinoma (ccRCC), which may translate to further approvals of RLTs in the future (Supplemental Tables S1 and S2).

Nuclear medicine departments have historically been structured around radioiodine treatment for benign thyroid disease and thyroid cancer. Over time, ^131^I-MIBG therapy (^131^iodine metaiodobenzylguanidine; mainly for the treatment of neuroblastoma in paediatric patients), radioembolisation of liver lesions using SIRT (Selective Internal RadioTherapy) spheres (radiolabelled microspheres regulated as medical devices), and bone pain palliation (e.g., with ^223^RaCl_2_ [Xofigo®]) were added as important therapies. Together, these therapies account for more than 80% of therapeutic nuclear medicine procedures. However, not all therapies are currently available in every EU country.^19^ For our assessment, we assume that all therapies authorised prior to ^177^Lu-DOTATATE in 2017 constitute an approximately constant baseline workload for nuclear medicine departments and will continue to do so for the foreseeable future, notwithstanding the substantial variability between countries.^19^ Therefore, the present work focuses exclusively on ^177^Lu-DOTATATE and ^177^Lu-PSMA-617, whose marketing authorisations (in 2017 and 2022, respectively) led to a significant broadening of patient access, and on the most promising new radiotheranostics in fast development. However, most European healthcare systems do not appear to be adequately prepared. The anticipated increase in patient numbers may overwhelm available treatment capacity and restrict future patient access to RLTs.^20^ Since PET/CT is obligatory for patient selection for RLT, insufficient imaging capacity may also limit patient access.

Central goals of the European Union (EU) Cancer Mission and Europe’s Beating Cancer Plan are to ensure access to innovative treatments and improve quality of life (QoL) for patients with cancer.^21^ Despite the uncertainties surrounding the timing of marketing authorisations and the uptake of RLTs by national healthcare systems, data on the evolution of the pool of eligible patients for RLTs are required to inform decisions on nuclear medicine service investments. The challenges of integrating radiotheranostics into clinical practice at the required scale have been described in detail elsewhere.^22^^,^^23^

The primary aim of this study was to project how the pool of biologically and clinically eligible patients for RLTs, across currently approved indications, potential authorisation extensions to earlier disease stages, and promising emerging therapies, could grow over the next decade. These maximum-eligibility estimates indicate the order of magnitude of the challenge and serve as input for a scenario analysis of the hypothetical uptake of RLTs in the four largest EU countries (Germany, France, Italy, and Spain) grounded in early uptake data from high-income countries to gauge how many patients in the pool are likely to receive RLT.^18^^,^^19^^,^^24^ These estimates should provide decision-makers with an indicative baseline for assessing their national healthcare landscape and for planning how to enable patient access to the potential benefits of RLT at the required scale.

## Methods

### Identification of products and relevant medical indications for RLT

Current and potential future indications for RLT were identified by searching for RLT products already authorised in the EU by the EMA and for advanced (phase 2 and 3) clinical trials listed in the US National Library of Medicine database.^25^^,^^26^ From these, associated/expected indications or sub-indications were summarised, accounting for factors including molecular target, disease stage, line of treatment, or required prior treatments (e.g., for ^177^Lu-PSMA-617 in mCRPC); the anticipated approval year was also estimated. Where more than one product was available for a given indication/sub-indication, patient numbers were calculated from the year in which the first authorisation for that indication was expected.

### Incidence- and prevalence-based estimates and data sources

The theoretical maximum size of the pool of patients biologically and clinically eligible for RLT was estimated based on patient numbers that comply with identified authorised and in-development RLT indications using two approaches, one incidence-based and the other prevalence-based, which can be considered complementary. Incidence counts the number of newly diagnosed cases of a given disease within a defined time interval, usually one year, and therefore may underestimate the number of patients requiring treatment by only considering those that have newly entered the patient pool. Prevalence accounts for all living patients diagnosed with a particular disease at any time during their lifetime, including those whose disease is in remission but could potentially relapse. As such, this approach overestimates the number of potential patients. Together, these approaches should provide an upper (prevalence-based) and lower (incidence-based) boundary for the estimated patient pools.

Cancer incidence data were obtained from the European Cancer Information System (ECIS), which collates data from national cancer registries and collaborates with these registries to ensure data quality, completeness, and comparability across EU countries.^27^ Estimates of the maximum pool size of patients eligible for RLTs were calculated for individual EU member states and combined for all member states (EU-27) for the years 2022, 2025, 2030, and 2035. Cancer prevalence data were obtained from the Institute for Health Metrics and Evaluation (IHME) database, which is open access, regularly updated, and offers systematic, comprehensive high-quality data on global cancer (Supplementary Material, page 17).^28^ Estimates were calculated for Germany, France, Italy, Spain, and the UK (EU-4 + UK) for the years 2022, 2025, 2030, and 2033, based on availability of data within the IHME database.

Incidence and prevalence data were supplemented with data from the UK Haematological Malignancies Research Network (HRMN) applied to population estimates from the Organisation for Economic Co-operation and Development (OECD),^29^^,^^30^ or from additional literature searches where data were unavailable for certain indications or cancer subtypes e.g., the ECIS database classifies cancer data by affected organ system using ICD-10 codes, with incidence data only available for the hierarchically highest ICD-10 code; therefore, additional literature searches were required for cancers such as NETs, which can occur in various organ systems.

The treatment-relevant prevalences and incidences were then estimated multiplying the prevalence and incidence figures per country and per year for each cancer type with the prevalence of target expression, the fraction of patients in the relevant stage of metastatic disease, receiving the proper line of therapy, and having received the required pretreatments to comply with the marketing authorisations or the selection criteria of the clinical trials, as appropriate. For some indications, the availability of multiple therapies or lack of access to therapy was also considered. The corresponding epidemiological data were gathered from literature. The variability of literature data was addressed through sensitivity analyses, giving rise to the ranges reported for incidence-derived estimates, as explained in the disease-specific subsections. Details are presented in the Supplementary Material (Supplemental Figs. S1–S5 and Tables S12–S23).

For the prevalence-derived datasets, all identified sources were carefully assessed to ensure that the data matched exactly the prevalent cancer population considered. In this context, the prevalence of the relevant metastatic disease stage in the prevalent population is the most important input. Due to the scarcity of such data, we found no meaningful way to perform a systematic sensitivity analysis of the prevalence-derived datasets.

### Role of the funding source

Research by the WiFOR Institute and medical writing support was funded by Novartis Pharma AG. In collaboration with the other authors, the funder was involved in the study design, collection, analysis, and interpretation of data, and the writing of the manuscript. The European Commission, Joint Research Centre did not receive external or additional funding and executed the work within its institutional mission.

### Ethics approval

Not required – the study used only publicly available aggregated data from the European Cancer Information System, the Institute for Health Metrics and Evaluation and peer-reviewed literature; no individual patient data were accessed.

## Results

### Identified therapeutics and relevant medical indications for RLT

Currently authorised and potential future indications and sub-indications for RLTs in Europe, including disease stage and line of treatment, are summarised in Supplemental Table S2, on which the estimation of the pool size of patients biologically and clinically eligible for RLTs is based.

### Incidence-based estimation of the patient pool eligible for RLT in the EU-27

The calculation of the patient pools for individual disease types and indications is detailed in the following sections, with incidence-based results summarised in Table 1. Detailed calculations and sensitivity analyses are presented in the Supplemental Figs. S3–S5 and Tables S12–S23.

**Table 1:** Summary of the estimated patient pool eligible for RLT in the EU-27 from 2022 to 2035 based on incidence data for indications/potential future indications for RLT.

Indication (sub-indication), by cancer type	2022	2025	2030	2035
Neuroendocrine tumors				
GEP-NET	9100–10,000	9300–10,100	10,900–11,200	10,900–11,200
Lung NET	–	–	5000	5000
PPGL	–	–	1400–2200	1400–2200
Total	**9100**–**10,000**	**9300**–**10,100**	**17,300**–**18,400**	**17,300**–**18,400**
Prostate cancer				
mCRPC, 3L	1400–3000	1400–3100	–	–
mCRPC, 3, 2L^a^	–	–	–	–
mCRPC, all lines	–	–	29,200–64,100	–
All mPC (mCRPC and mHSPC)	–	–	–	93,000–136,000
Total	**1400**–**3000**	**1400**–**3100**	**29,200**–**64,100**	**93,000**–**136,000**
Haematological malignancies				
AML^b^	–	–	9900–12,500	10,400–15,300
LPL/WM	–	–	1600–2500	1600–2500
MM	–	–	200	200
Total	–	–	**11,700**–**15,200**	**12,200**–**18,000**
Kidney cancer				
ccRCC (metastatic, 2L)	–	–	–	**3100–8100**
Brain and CNS tumors				
Malignant meningioma	–	–	200–1600	200–1600
Neuroblastoma/ CNS (children)	–	–	100–500	100–500
Total	–	–	**300**–**2100**	**300**–**2100**
All indications				
Total	**10,500**–**13,000**	**10,700**–**13,200**	**58,500**–**99,800**	**125,900**–**182,600**

Numbers are rounded to the nearest hundred.

Where available, incidence data for 2022 and projected incidence for 2025, 2030, and 2035, were obtained from ECIS. For neuroendocrine tumors, incidence data were obtained by literature search.

1L, first-line treatment; 2L, second-line treatment; 3L, third-line treatment; AML, acute myeloid leukemia; GEP-NET, gastroenteropancreatic neuroendocrine tumor; LPL, lymphoplasmacytic lymphoma; mCRPC, metastatic castration-resistant prostate cancer; mHSPC, metastatic hormone sensitive prostate cancer; MM, multiple myeloma; mPC, metastatic prostate cancer; NET, neuroendocrine tumor; PPGL, pheochromocytoma and paraganglioma; WM, Waldenström macroglobulinemia.

a 2L treatment of mCRPC, as approved by the FDA in 2025, could contribute 8700–19,000 patients to the pool; authorization of this indication by the EMA is delayed, therefore only 3L treatment is considered for 2025.

b Estimate based on number of stem cell transplantations, the upper limit assumes a 3% increase per year after 2022.

#### Neuroendocrine tumours

NETs develop in various organs, with GEP-NETs (occurring in digestive tract organs such as the stomach, small intestine, appendix, colon, cecum, rectum, and pancreas) accounting for approximately 65% of all NETs, lung and bronchi for 25%, and NETs in other organs for the remaining 10%.^31^ Although relatively rare, the incidence of NETs has increased considerably in recent decades.^32^ Since ECIS data are encoded using ICD-10 codes, incidence data for NETs were obtained through literature searches, with overall population data for EU member states obtained from ECIS. ^177^Lu-DOTATATE is currently authorised for unresectable or metastatic, progressive, well-differentiated (G1 and G2), SSTR2 positive GEP-NETs.^12^ GEP-NET incidence ranges from 4.66/100,000 to 4.76/100,000,^32^ with SSTR2 expressed in approximately 87% of GEP-NETs, and 80–85% classified as G1 or G2.^33^ About 60% of patients with GEP-NETs have inoperable primary tumors at diagnosis.^34^ With increasing experience with RLT for the treatment of GEP-NETs, and at least two RLT products in clinical trials (Supplemental Table S2), there is potential for authorisation in poorly differentiated G3 GEP-NETs, thereby covering >96% of patients with GEP-NETs. This would increase the pool of eligible patients to approximately 10,900–11,200 per year from 2030 (Table 2, Supplemental Fig. S3 and Table S12).^17^

**Table 2:** Estimation of the patient pool eligible for RLT in the EU-4, the UK, and the EU-4 + UK from 2022 to 2033, derived from prevalence data for indications/potential future indications for RLT, making use of the data compiled in Supplemental Tables S5–S9.

Indication (sub-indication), by cancer type	2022	2025	2030	2033
Neuroendocrine tumors				
NET (metastatic, 2L)	–	–	7000	7000
GEP-NET (G1–G2, metastatic, 2L)	19,500	19,700	19,800	19,900
GEP-NET (G2–G3, metastatic)	–	–	6500	6500
Total	**19,500**	**19,700**	**33,300**	**33,400**
Prostate cancer				
mCRPC (2L–3L)	1400	1500	11,700	12,500
mHSPC	–	–	24,200	25,700
OMPC	–	–	6000	6400
Total	–	**1500**	**41,900**	**44,600**
Hematological malignancies				
AML	–	–	43,800	45,500
LPL/WM	–	–	2600	2600
MM	–	–	500	500
Total	–	–	**46,900**	**48,600**
Kidney cancer				
ccRCC (metastatic, 2L)	–	–	–	**3200**
Brain and CNS tumors				
Neuroblastoma/ CNS (children)	–	–	**9500**	**9500**
Total EU-4	20,900	21,200	131,600	139,300
Total UK	5400	5500	25,600	27,100
Total EU-4 + UK	**26,300**	**26,700**	**157,200**	**166,400**

Numbers are rounded to the nearest hundred.

2L: second line of treatment; AML: acute myeloid leukemia; ccRCC: clear cell renal cell carcinoma; CNS: central nervous system; G1: Grade 1; GEP-NET: gastroenteropancreatic neuroendocrine tumor; mCRPC: metastatic castration resistant prostate cancer; mHSPC: metastatic hormone sensitive prostate cancer; LPL/WM: lymphoplasmacytic lymphoma/Waldenström Macroglobulinemia; MM: multiple myeloma; NET: neuroendocrine tumor; OMPC: oligometastatic prostate cancer; RLT, radioligand therapy.

In addition to GEP-NETs, SSTR2 is expressed by NETs in other organ systems, albeit at lower expression levels.^35^ Based on ongoing clinical trials, there is potential for RLT authorisations for lung NETs and pheochromocytomas and paragangliomas (PPGL) by 2030. With a reported incidence of 1.47/100,000 and SSTR2 expression of 75%, lung NETs could add an estimated 5000 per year to the patient pool.^32^^,^^36^ PPGL, a rare group of NETs with an incidence between 0.417/100,000 and 0.661/100,000 per year,^37^ and 74.8% of tumors expressing sufficient SSTR2 levels for targeted treatment,^38^ may add 1400–2200 per year to the patient pool to give an overall estimate of the patient pool with NETs eligible for RLT of 17,300–18,400 by 2030 (Table 1, Supplemental Tables S13 and S14).

#### Prostate cancer

Based on reports indicating that 79–87% of prostate cancer patients exhibit PSMA-expressing lesions and an incidence of 330,492 prostate cancer cases in the EU-27 in 2022 (ECIS data), there were an estimated 262,000–288,000 new PSMA-positive patients in the EU-27 in 2022.^39^^,^^40^ Currently, ^177^Lu-PSMA-617 is authorised in the EU for treatment of PSMA-positive mCRPC after treatment with an ARPI and taxane-based chemotherapy.^15^ Approximately 95% of prostate cancer patients have local or locally advanced disease at the time of first diagnosis; after initial treatment with surgery and/or external beam radiotherapy, 30–40% of these develop metastatic disease, and 10–20% eventually develop mCRPC.^41^^,^^42^ Consequently, 26,000–57,000 new PSMA-positive patients per year are likely to develop mCRPC in the EU-27. Leith et al. report that 31.83% and 5.19% of mCRPC patients receive a second line (2L) and third line (3L) of treatment, respectively, yielding 1400–3000 patients per year in 2022 for the currently authorised indication of ^177^Lu-PSMA-617 (Table 2).^43^ If the EMA authorises ^177^Lu-PSMA-617 for chemotherapy-naïve mCRPC, in line with the 2025 US label update,^16^ this number is expected to increase to approximately 8,700–19,000 patients (Supplemental Fig. S4 and Table S16).

Potential authorisation of PSMA-targeted RLT for metastatic hormone-sensitive prostate cancer (mHSPC), based on the PSMAddition trial (NCT04720157), combined with the estimated 30–40% of prostate cancer patients progressing to metastatic disease,^42^ could increase the patient pool with metastatic prostate cancer biologically and clinically eligible for RLT to 93,000–136,000 per year after 2030 (Table 2). These figures represent a maximum theoretical ceiling, as the advanced median age of patients with metastatic disease influences treatment decisions. Increasing age is significantly associated with a decreased likelihood of receiving definitive therapy (radiotherapy or surgery).^44^ Data from the US show that access to definitive therapy declines from 80.9% among patients aged 75–79 years to 55.2% among those over 80 years, who are mainly managed with androgen deprivation therapy.^44^ Life expectancy is considered an important factor in decision-making for elderly patients, when tumours progress slowly and the risk of prostate cancer-related death is judged to be lower than the competing risk of age-related mortality.^44^ Moreover, the marketing authorisation may initially be restricted as the longer life expectancy of younger mHSPC patients may motivate regulators to require longer follow-up periods to assess long-term risks.

#### Haematological malignancies

Several indications for haematological malignancies with hypothetical authorisations in 2030 were identified (Supplemental Table S2) based on ongoing phase 2 and 3 trials, including acute myeloid leukaemia (AML), lymphoplasmacytic lymphoma (LPL), Waldenström macroglobulinaemia (WM), and multiple myeloma (MM) (see Supplementary Material, pages 39–45).

For AML, the phase 3 SIERRA trial (NCT02665065) is investigating Iomab-B with a reduced intensity conditioning regimen and protocol-specified allogeneic haematopoietic stem cell transplantation (HSCT) in patients with active relapsed or refractory disease. Iomab-B is a radio-immunoconjugate targeting CD45, which is expressed on the leukaemia cells of 97.2% of patients with AML.^45^ In 2012, 4784 allogeneic HSCT procedures were reported for AML in Europe,^46^ increasing to 6676 in 2017, with the procedure still considered underused.^47^ Assuming the same growth rate, approximately 13,000 allogeneic HSCT procedures are estimated for the hypothetical authorisation year of 2030 in this clinical setting, corresponding to up to 12,500 patients with CD45-positive disease. Although the phase 2 CLOVER-1/CLOVER-WaM trial (NCT02952508) of ^131^I-iopofosine (CLR 131) included multiple B-cell malignancies, the parts with chronic lymphocytic leukaemia (CLL)/small lymphocytic lymphoma (SLL), marginal zone lymphoma (MZL), mantle cell lymphoma (MCL), and diffuse large B-cell lymphoma (DLBCL) patients have closed, with no peer-reviewed publications identified, and no progression to phase 3 clinical trials, therefore only MM and LPL/WM were included in the estimate. Based on an incidence rate of 0.36/100,000 to 0.55/100,000,^48^ an estimated 1600–2500 patients with LPL/WM per year could become candidates for RLT in the EU-27 from 2030 onwards.

In a small number of heavily pre-treated patients with MM (median of nine prior treatments) in CLOVER-1, ^131^I-iopofosine treatment achieved stable disease in all patients with an overall response rate of 50%.^49^ If the eligibility criteria for MM patients in CLOVER-1 are kept i.e., a requirement for at least five prior treatment regimens, less than 0.5% of MM patients (approximately 200 patients per year) could become candidates for this treatment from 2030 as a last-resort option.^50^

#### Clear cell renal cell carcinoma

The indication of metastatic/2L ccRCC was identified based on the phase 2 trial of ^177^Lu-DOTA-girentuximab (LX250; NCT05239533), with hypothetical authorisation after 2030 (Supplemental Table S2). ^177^Lu-DOTA-girentuximab targets carbonic anhydrase 9 (CAIX), a transmembrane carbonic anhydrase expressed in 91.7–97% of ccRCCs,^51^^,^^52^ which accounts for 59.5–72.8% of all kidney cancers.^53^^,^^54^ Surgery is the 1L treatment for localised disease,^53^ and up to 50% of patients treated with partial or radical nephrectomy for non-metastatic renal cell carcinoma are considered at high risk for metastatic progression,^55^ while 13% of patients with ccRCC have metastatic disease at initial diagnosis.^56^

While monotherapies and combination therapies with immune checkpoint inhibitors (ICI) and tyrosine kinase inhibitors (TKI) have improved survival, nearly all patients develop resistance to TKI and ICI therapy.^57^^,^^58^ This motivates the development of RLTs with distinct mechanisms of action. However, girentuximab has a long half-life in blood; therefore, myelotoxicity may need to be controlled e.g., through combination treatments with an ICI. Based on data from Muselaers et al.^59^ it is assumed that even with a reduced dosage scheme, toxicity will limit therapeutic use to approximately 10–20% of patients who have, or will develop, metastatic disease.^59^ Faster targeting and blood clearance with peptide vectors could address a broader patient population; however, this approach is currently being evaluated in phase 1/2 clinical trials and has not been considered.

The ECIS projection for 2035 is 101,062 kidney cancer cases, of which 59.5–72.8% are estimated to be ccRCC with 91.7–97% of these expressing CAIX. Considering half of the patients with localised disease at high risk after nephrectomy, in addition to the patients with metastatic disease at diagnosis, up to 56.5% of CAIX-positive patients with ccRCC could be considered candidates for RLT; however, due to myelotoxicity, only approximately 3100–8100 may benefit from RLT (Supplemental Fig. S5 and Table S21).

#### Brain tumors and central nervous system tumors

Several types of brain and central nervous system (CNS) tumors express somatostatin receptors.^35^ A number of phase 2 trials are ongoing investigating RLT for malignant meningioma and neuroblastoma (Supplemental Table S1), which could lead to potential authorisations for ^177^Lu-DOTATATE and ^131^I-omburtamab, respectively, around 2030 (Supplemental Table S2).

Meningiomas account for about 30% of all intracranial tumours, with an incidence of 7.86/100,000 per year; however, most are histologically benign and can be treated surgically. The incidence of malignant meningiomas requiring systemic and aggressive treatment is reported to range between 0.18/100,000 and 0.36/100,000 per year^60^; although, incidence rates as low as 0.054/100,000 per year have been reported.^61^ SSTR2 expression in meningiomas also varies, ranging from 64 to 100%.^62^ Based on these values, the number of patients who could potentially benefit from RLT in the EU-27 from 2030 is estimated at 200–1600 per year (Table 2).

Neuroblastoma, the most common extracranial solid tumor in children, is associated with a poor prognosis.^63^ Incidence rates range from 0.012/100,000 to 0.10/100,000,^61^^,^^64^ and B7-H3/CD276 is expressed in neuroblastoma tissue in 82% of patients,^65^ corresponding to approximately 100–500 of patients per year in the biologically and clinically eligible patient pool for RLT in the EU-27 from 2030 (Supplemental Tables S22 and S23).

### Prevalence-based estimation of the patient pool eligible for RLT

The estimation of the patient pool eligible for RLT based on cancer prevalence was performed for the EU-4 and the UK. Prevalence data for each identified indication or sub-indication were obtained from the IHME database or by literature search, where IHME data were not available. Supplemental Table S4 summarises the input parameters, including disease prevalence, target marker expression levels, patient fractions for relevant lines of treatment for each indication and sub-indication, as well as the corresponding information sources. The results for the EU-4 are compiled in Table 2 for the respective indications and lines of treatment, where applicable. In summary, the prevalence-derived patient pool for the EU-4 could increase from 21,200 in 2025 to 139,300 in 2033, and the pool in the UK from 5500 to 27,100 during the same period. More detailed and country-specific estimates are provided in the Supplemental Tables S5–S10.

### Summary of the estimates of the patient pool eligible for RLT patients

**Fig. 1:**
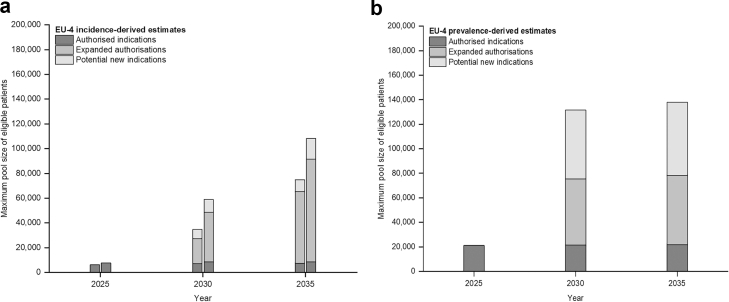
(a) Incidence-derived and (b) prevalence-derived estimates of the maximum patient pool biologically and clinically eligible for radioligand therapy in the EU-4 (Germany, France, Italy, and Spain) for authorised indictions for ^177^Lu-DOTATATE and ^177^Lu-PSMA-617, potential authorisation expansions to earlier disease stages, and potential new authorisations of radioligand therapies in phase 2 or 3 clinical trials. In panel (a), the columns represent the lower and upper bound of the sensitivity analysis.

Table 1 (and Supplemental Fig. S7) presents the incidence-derived maximum sizes of the patient pools eligible for RLT for the EU-27 for 2025, 2030, and 2035. Lower and upper bounds were estimated in a sensitivity analysis of the biological and clinical input parameters. Fig. 1a and b compare estimates for the EU-4 derived from incidence and prevalence data, respectively. The Figures distinguish between contributions to the RLT-eligible patient pool from (i) authorised indications for ^177^Lu-DOTATATE and ^177^Lu-PSMA-617; (ii) anticipated marketing authorisations for these products to treat earlier stages of disease; and (iii) new products, which are associated with a higher development and regulatory risk if clinical trials do not report positive results. The expansion of patient pools is primarily driven by ^177^Lu-DOTATATE and ^177^Lu-PSMA-617. Notably, extensions to the marketing authorisations for these two products carry regulatory uncertainty, as the EMA may delay, restrict, or decline authorisation for certain indications. As expected, the prevalence-derived estimates are higher than those derived from incidence. Since both datasets reflect a “common sense” assessment covering different countries and populations, the agreement between them is reasonable, indicating a maximum eligible-patient pool of approximately 110,000–140,000 patients in 2035.

Table 3 compiles the overall evolution of the estimated patient pools eligible for RLT in EU member states and the UK, based on both incidence data and, where available, prevalence data. The ranges provided reflect the sensitivity analysis applied to refine the incident population according to the biological and clinical characteristics required for the current or anticipated authorisations.

**Table 3:** Country-specific information and estimate of the patient pool biologically and clinically eligible for RLT.

Country	^177^Lu-RLTs available in 2020^a^	Population 2022 (ECIS)	Time from EMA approval – reimbursement (in days)^c^	Eligible patient pool		
				2025	2030	2035
Austria	Yes	8,978,929	250	200–300	1100–1900	2300–3400
Belgium	Yes	11,617,623	600	300–400	1800–3100	3900–5700
Bulgaria	No	6,838,937	780	200	600–1000	1200–1700
Croatia	No	3,862,305	640	100	500–900	1100–1600
Cyprus	No	904,705	570	<100	100–200	200–300
Czechia	Yes	10,516,707	460	300	1400–2300	2900–4300
Denmark	Yes^b^	5,873,420	140	100–200	900–1500	1900–2800
Estonia	Yes	1,331,796	670	<100	200–300	400–600
Finland	Yes	5,548,241	370	100–200	900–1600	2000–2900
France	Yes	67,871,925	550	1400–2100	9900–17,000	21,200–30,800
France (∗)				5500	25,700	27,000
Germany	Yes	83,237,124	100	2000–2500	11,300–19,500	24,800–36,000
Germany (∗)	Yes			7000	65,300	68,100
Greece	Yes	10,459,782	570	200–300	1300–2100	2600–3800
Hungary	No	9,689,010	560	200–300	1100–1900	2400–3400
Ireland	No	5,060,004	590	100–200	700–1300	1700–2500
Italy	Yes	59,030,133	400	1400–1700	7300–12,200	15,300–22,200
Italy (∗)	Yes			4800	21,500	25,500
Latvia	No	1,875,757	860	<100	200–400	600–800
Lithuania	No	2,805,998	920	100	500–800	1100–1600
Luxembourg	No	645,397	420	<100	100	200–300
Malta	No	520,971	Not available	<100	100	100–200
Netherlands	Yes	17,590,672	320	400–500	2300–3800	4700–6800
Poland	Yes	37,654,247	830	900–1100	4300–7100	8700–12,600
Portugal	Yes	10,352,042	800	200–300	1400–2300	2900–4200
Romania	No	19,042,455	830	400–500	1900–3200	3900–5600
Slovakia	Yes	5,434,712	710	100–200	700–1100	1500–2100
Slovenia	Yes	2,107,180	540	100	300–500	800–1100
Spain	Yes	47,432,893	720	1100–1400	6000–10,500	13,600–19,700
Spain (∗)				3800	17,000	18,400
Sweden	Yes	10,452,326	370	300	1700–3100	3900–5700
UK (∗)	Yes		240	5500	25,600	27,100
EU-27		446,735,291		**10,700**–**13,200**	**58,500**–**99,800**	**125,900**–**182,600**
EU-4		257,572,075		6100–7600	34,500–59,000	74,800–108,900
EU-4 (∗)				21,100	131,600	139,300

Incidence-derived data are presented for all EU-27 countries and prevalence-derived data are presented for the EU-4 (Germany, France, Italy, and Spain) and UK (marked with (∗); for these data, the last column refers to 2033). Numbers are rounded to the nearest hundred.

a ^177^Lu-therapies were considered as not available in 2020 based on Lightvoet et al.^19^ when the number of reported procedures using ^177^Lu-DOTATATE or ^177^Lu-PSMA was zero (cf. Supplemental Table S3).

b Although data on nuclear medicine procedures in Denmark are not reported,^19^ Denmark confirmed the availability of ^177^Lu-DOTATATE.

c The number of days between EMA approval and the local reimbursement decision was extracted from Fig. 2 in Hofmarcher et al.^66^ No data are available for Malta.

## Discussion

The estimate of the patient pool eligible for RLTs is subject to substantial uncertainty due to variability in the clinical literature used to define patient populations in specific clinical settings, which give rise to the ranges reported for the incidence-based estimates. Additional uncertainty arises from the assumptions regarding timelines for granting marketing authorisations, or modifications thereof, as well as development risk for products targeting new cancer types. Table 4 summarises the assumptions made, the limitations of our approach, and the mitigation measures adopted.

**Table 4:** Compilation of the essential assumptions used to calculate the patient pool eligible for RLT and the uptake scenario, along with the limitations of the approach and the mitigation measures where possible.

Estimate of the maximum size of the pool of eligible patients		
Assumptions	Limitations	Mitigation/ Comment
Authorised products/indications: In each EU country all patients count towards the pool if their clinical status complies with the medical indications stipulated in the marketing authorisation (MA).	National health care systems may restrict access to a subset of the authorised medical indications due to limited resources, clinical and cost effectiveness considerations, perceptions of uncertainty around aspects of the (clinical and/or economic) evidence, or due to country-specific national treatment guidelines or defined quota. In some healthcare systems, the therapy may not be available (cf. Table 3).	Population restrictions and risk mitigation strategies usually apply to mitigate concerns around incomplete or early phase evidence.
Extensions of MAs or new MAs will be granted for the indications investigated in the respective clinical trials.	EMA and/or national regulatory bodies (including Health Technology Assessment agencies) may restrict medical indications or reject authorisation.	
For authorised indications, patients can be treated in the same year the MA is granted or modified.	In the EU, there is no immediate link between a central marketing authorisation and reimbursement decisions taken in individual Member States. The delay between regulatory approval and reimbursement decisions may be up to 3 years (cf. Table 3) and not all products will receive reimbursement in each country.^66^	Delays of 1–3 years are not expected to materially affect the estimated sizes of the eligible patient pools reported for 2030 and 2035. The effect on the data for 2030 is mitigated by the fact that for most indications there are several products considered in different trials by different sponsors.
The trials compiled in Supplemental Table S2 will successfully lead to extended MAs and/or new authorised products.	Some clinical trials may not be sufficiently successful and be delayed/rejected by regulatory bodies and/or trigger strategic decisions by sponsors in response.	Despite the rapidly evolving research landscape of RLT,^6^ the development risk has already been reduced by excluding phase 1 clinical trials irrespective of how promising the new targets and indications are.
The timelines for obtaining an extended or new MA as compiled in Supplemental Table S2 can reasonably be projected.	Particularly for new products and trials in phase 2 the year of authorisation is a reasonable hypothesis. For extensions to treat a patient population with a longer life expectancy, regulatory bodies may have additional requests requiring additional clinical trials and granting extensions may be delayed.	Without guaranteed accuracy, the estimates are partially based on industrial experience with regulatory approvals.
Literature to determine target expression, population progression to specific advanced stages of disease, fractions of the population that receive a certain line of treatment, and other parameters to determine the size of the patient pool are adequately precise and relevant for the patient population concerned.	Data reported in literature may exhibit a considerable range due to different research focus, different methodologies applied, geographically different populations investigated, and different national treatment guidelines and peculiarities. The scarcity of data necessitates applying data from a specific country or region (e.g., the US) to the EU-27 or EU-4. The transferability of data to each EU country could not be verified. There is a general scarcity of data precisely matching the prevalence of certain features with the prevalent population concerned.	Whenever possible, European data were used, notwithstanding appreciable differences among EU countries. The wide range of data in the literature has been considered in a sensitivity analysis leading to a wide range in the incidence-derived data.
All patients that meet the criteria of target expression, stage of disease, line of treatment, and necessity for systemic treatment will be counted eligible.	Age-related effects that could reduce the number of eligible patients in the pool, such as comorbidity, frailty, and competing therapies, could not be evaluated quantitatively and considered for the pool estimates.	In some cases, competitive treatments led to the exclusion of whole indications from the analysis.
The RLTs considered in this assessment will have negligible effects in terms of replacing radiopharmaceutical therapies established before 2017.	The introduction of ^177^Lu-DOTATATE and ^177^Lu-PSMA might affect the utilisation of ^131^I-MIBG and ^223^RaCl_2_ for neuroendocrine tumour treatment and bone pain palliation, respectively.	^131^I-MIBG is only used in approximately 500 patients per year in the EU;^24^^223^RaCl_2_ is considered a combination treatment with ^177^Lu-PSMA.^67^ This limits the impact of potential replacement.

Uptake scenario for Germany, France, Italy, Spain and the UK		
Assumption	Limitation	Mitigation/comment
Preliminary real-world data on the uptake of therapy (Table 5) are considered to incorporate effects on patients treated with competing therapies, due to age-related effects (comorbidities, frailty, etc.) or limited availability of RLT.	It is questionable whether initial data on the actual numbers of treated patients compared with the number of patients considered eligible are already approaching saturation or will increase further as RLTs gains awareness. The real impact of comorbidity and frailty on the real-world data could not be assessed.	The uptake scenario has only been applied to Germany, France, Italy, and Spain (EU-4), which have well-developed nuclear medicine infrastructure (comparable to the US, considered as high-income countries). These real-world data should not be applied to countries with less developed nuclear medicine infrastructure (cf. Table 3)
The medical indications in real-world utilisation data correspond to those applied to estimate the European patient pools eligible for RLT. Clinical and biological eligibility criteria are the same.	The number of eligible patients may be overestimated by Abdel-Wahab et al.^24^ and Czernin et al.^18^ Abdel-Wahab et al.^24^ only report aggregated data for high-income countries; Czernin et al.^18^ reports precise utilisation data, but only for the US.	In 2024, ^177^Lu-PSMA-617 was authorised equally in the EU and the US for the treatment of mCRPC after therapy with an androgen receptor pathway inhibitor (ARPI) and a taxane-based chemotherapy. Therefore, utilisation data in the US and the EU for 2024 correspond to the same authorisation scheme. Overestimated eligible patient populations will lead to smaller ratios of treated/eligible patients in support of a more conservative uptake scenario.
For the scenario analysis, it is assumed that uptake was not significantly different between countries in terms of reimbursement schemes and clinical practice.	The uptake of new RLTs in EU countries will be heterogeneous due to differences in resources, infrastructure, interpretation of the evidence supporting these therapies, and national treatment guidelines. In some EU countries, nuclear medicine departments cannot offer all therapies that are considered standard of care in others (cf. Table 3). The US is known for the highest healthcare spending per capita.	The uptake scenario analysis focused on a subgroup of European high-income countries (Germany, France, Italy, and Spain) with well-developed nuclear medicine infrastructure i.e., those that are most similar to the US.
The first real-world data used for the assessment provide ratios of treated/eligible patients that will also be applicable to extended or newly authorised treatments in the future.	National healthcare systems may restrict or prioritise access to RLT for economic or capacity reasons, especially in the absence of health technology assessments based on large patient numbers and real-world data. Some of the considered future indications may not ultimately be clinically successful.	Initial health economic assessments point towards economic benefits when RLT is applied in earlier lines of treatment.^75^ The development risk has been considered by removing AML and oligometastatic prostate cancer from the scenario.
Radioligand therapies will be available at the required scale without interruptions of supply or radionuclide shortages.		The availability of radionuclides, in particular, ^177^Lu and ^225^Ac, will satisfy anticipated future utilisation (see Supplementary Material p.53)
Patient preference will not have an impact on RLT uptake.	The extent to which patients might have concerns with the use of radiopharmaceuticals is unknown. However, patients contribute to the healthcare decision-making process in many EU countries.	Potential for a favourable safety profile and QoL maintenance could encourage patients to opt for RLTs. Real-world data showing improved overall survival would support this assessment.

Combination therapies in current clinical trials are not expected to increase the pool of eligible patients, as they combine therapies for the same medical indications. However, combining therapies with different mechanisms of action can involve different medical specialties. This may contribute to a broader acceptance of RLTs and increase the fraction of eligible patients receiving RLT. However, while ICIs or chemotherapies may be co-administered with RLT in radiation-shielded rooms; the reverse is not feasible in an oncology ward, and handling of radiopharmaceuticals requires specific expertise and authorisations.

Tandem regimens combining beta-emitting RLT with future alpha-emitting RLT directed at the same target and combinations with ICI are considered promising approaches.^68^ There is increasing evidence that RLT rechallenge in NET and prostate cancer patients is feasible and tolerable in cases of disease progression after initial RLT.^69^^,^^70^ Retreatment requiring additional RLT cycles is more likely to increase the effort per patient than the number of patients. However, literature does not yet allow estimation of the frequency and timing of rechallenge.

A forecast how many patients in the eligible patient pools will ultimately be treated with RLT depends on several parameters that are difficult to disentangle (Table 4). In the following, we provide a scenario based on the currently scarce real-world data on the utilisation of authorised RLTs in healthcare systems and consider development risk for some indications in this context. Table 5 compiles data on the number of treated patients compared with the estimated pool of eligible patients. The numbers from Abdel-Wahab et al. are based on therapy utilisation per million population in high-income countries.^24^ Since uptake of ^177^Lu-PSMA-617 in the US (approximately 11,000 patients treated in 2024) appears to be faster than in Europe, the mean value of 14.2 per million may overestimate European utilisation and underestimate US utilisation.^18^ Abdel-Wahab et al. base their estimate of eligible patients on a stage IV prostate cancer prevalence of 18% within the prevalent prostate cancer population, without distinguishing between mCRPC and mHSPC.^24^ Consequently, this may overestimate the number of eligible patients based on current EMA authorisations.

**Table 5:** Comparison of real-world RLT utilisation data^a^ with eligible patient pools.

Source	Number of treated patients			Number of patients in eligible patient pool			
	Abdel-Wahab et al.^b^	Czernin et al.	Ligtvoet et al.^c^	Abdel-Wahab et al.^d^	Czernin et al.	Present work	
						Incidence-based	Prevalence-based
Year	2024	2024	2020	2024	2024	2022	
	^177^Lu-PSMA-targeting radioligand therapies (+ ^225^Ac-PSMA)^e^						
EU-27	6500	–	1800 (+400)	26,000	–	1400–3000	–
EU-4	3800	–	1400 (+400)	15,000	–	800–1800	1400
UK	1000	–	350	4000	–	–	350
US	4900	11,000	–	19,000	34,000	–	–
	^177^Lu-DOTATATE						
EU-27	7300	–	3100	–	–	9100–10,000	–
EU-4	4300	–	2400	–	–	5300–5800	19,500
UK	1100	–	900	–	–	–	5100
US	5500	3000	–	–	7500	–	–

a RLT utilisation data were obtained from Abdel-Wahab et al. for 2024 in high-income countries, Czernin et al. for 2024 in the US, and Ligtvoet et al. for 2020 in Europe (see Supplemental Table S3), and compared with the estimated patient numbers for 2024 by Abdel-Wahab et al. and Czernin et al. and the estimated eligible patient pool for 2022 in the current work.^18^^,^^19^^,^^24^

b Treated patients were calculated based on a utilisation per million population of 16.3 for ^177^Lu-DOTATATE, 14.2 for ^177^Lu-PSMA, and 0.3 for ^225^Ac-PSMA for high income countries (see Table 2 from Abdel-Wahab et al.).^24^

c Data were collated from the country fact sheets in Appendix C from Ligtvoet et al. (see Supplemental Table S3).^19^

d Patients eligible for PSMA-targeting RLT were calculated using a rate of eligible patients of 56.7 per million population in high-income countries (see Section 3 from Abdel-Wahab et al.^24^).

e Data in brackets show the additional patients treated with ^225^Ac-PSMA in Germany based on 1658 procedures (see Supplemental Table S3), assuming 4 procedures per patient.

Czernin et al. reported 11,000 patients treated with ^177^Lu-PSMA-617 in 2024 in the US, based on published commercial data.^18^ Considering 11,000 treated patents, the resulting treated-to-eligible ratio ranges from 58% (11,000/19,000) to 32% (11,000/34,000). However, Czernin et al. estimated the number of eligible patients from mortality data and did not consider regulatory restrictions by line of treatment.^18^ Therefore, the estimate of 34,000 eligible patients is likely to be overestimated, and the corresponding 32% ratio may be underestimated.

By contrast, the figures reported by Ligtvoet et al., reflecting European utilisation of ^177^Lu-PSMA in 2020 (when the therapy was available only through early access schemes), align with our estimates for 2022 and are consistent with the medical need for ^177^Lu-PSMA.^19^ For France, Massard et al. reported 3198 patients treated with ^177^Lu-PSMA-617 over a three-year period ending January 2025, which is three times our prevalence-based estimate.^71^ Therefore, we used 45% as a conservative estimate of the average proportion of patients from the pool eligible for ^177^Lu-PSMA-617 who may receive treatment.

For ^177^Lu-DOTATATE, Czernin et al. report 3000 treated patients and 7500 eligible patients; thus, 40% of eligible patients received RLT.^18^ From Ligtvoet et al., we derive a treatment ratio of approximately 33% in the EU-27 (in 2020) and approximately 40% in the EU-4, which may reflect unequal access to RLT.^19^ In 2020, approximately 50 million EU citizens had no access at all to RLT (Table 3). The utilisation estimates by Abdel-Wahab et al. are based on an average value across high-income countries, which may overestimate European and underestimate US utilisation.^24^

**Fig. 2:**
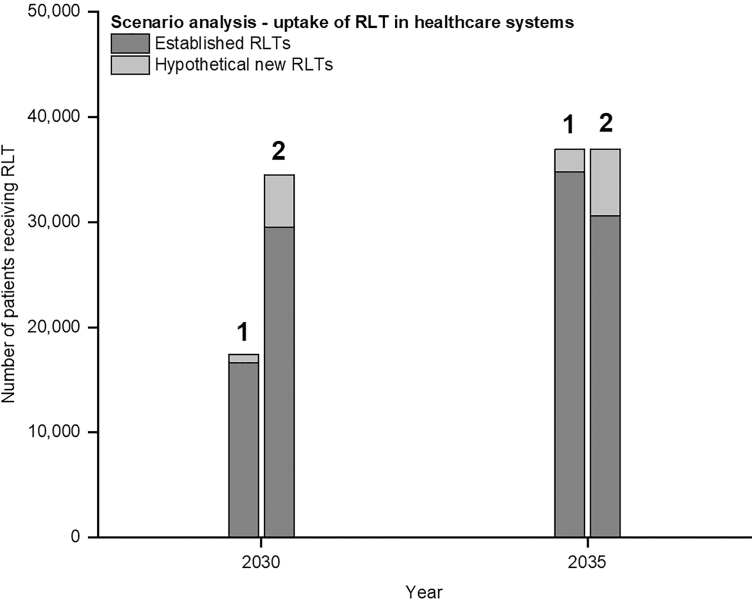
Hypothetical uptake scenario of radioligand therapy in the EU-4 (Germany, France, Italy, and Spain). Estimates are based on (1) incidence-derived data (mean values from the sensitivity analysis are presented) and (2) prevalence-derived data.

With these data, we constructed a scenario for the EU-4 countries to estimate how many patients from the eligible pool might be treated with RLT. We assume that 45% of prostate cancer patients will receive RLT during their disease course; for all other indications we assume 40%. Development risk was considered by hypothesizing that an AML RLT may face competition from immune and cell-therapies and may be unable to demonstrate superiority, and that treatment for oligometastatic prostate cancer might not enter clinical practice. Treatments for haematological and brain cancers in later lines of therapy in small patient numbers were retained in this scenario. The calculations are presented in Supplemental Table S24 and shown in Fig. 2. For 2030 the scenario yields between 17,000 and 35,000 patients treated in the EU-4, based on incidence-derived and prevalence-derived estimates, respectively. For 2035, the scenario yields approximately 37,000 patients treated with RLT. Fig. 2 shows that ^177^Lu-DOTATATE and ^177^Lu-PSMA account for most treatments; new therapies contribute only marginally to uptake, which is consistent with initial authorisations limited to later lines of therapy. A hypothetical breakdown for EU-27 countries is presented in Supplemental Table S25. However, these projections compare unfavourably with available information on treatment capacity.

A recent survey-based study estimated that the current capacity available for RLT administration at clinical centres in the EU-4 is 25,000–28,000 patients (see Supplemental Table S26), which appears insufficient to accommodate 17,000–35,000 patients in 2030 and falls below our scenario estimate of 37,000 treated patients in 2033/2035.^20^ An additional report from France estimates a need for 34,200 treatment cycles in 2024 – more than 2.5 times higher than the available free capacity for 13,400 treatment cycles.^72^ A complete RLT regimen with ^177^Lu-DOTATATE or ^177^Lu-PSMA-617 requires up to 4 or 6 treatment cycles, respectively. An assessment in Germany concluded that capacity may be sufficient in the short term but was likely to become limited in the medium term.^73^ The situation is for sure worse for more than 50 million European citizens living in countries that had no access to ^177^Lu-RLTs in 2020 (Table 3).

After the administration of radiopharmaceuticals, patients represent a source of radiation and therefore appropriate precautions are required. In many countries, radiopharmaceutical therapies are administered in outpatient settings, whereas other countries require extended hospitalisation, which directly affects treatment capacity. Harmonisation of patient-release criteria, based on robust data on radiation exposure to caregivers, family members, and members of the public is desirable. However, it will not increase capacity in countries that already rely predominantly on outpatient delivery.

The resource-related, logistical, infrastructural, financial, political, and regulatory challenges associated with delivering RLTs at the required scale have been extensively reviewed elsewhere.^22^^,^^23^ For the effective use of RLTs, nuclear medicine physicians, oncologists, and other medical specialties must collaborate closely, ideally in multidisciplinary tumour boards. The administration of radiopharmaceuticals requires a multidisciplinary skill mix, including radiochemists and radiopharmacists, specialised nurses, medical physicists, and radiation protection staff. Likely, staff availability may be a bottleneck for the rapidly increasing utilisation of RLTs. Education and training of medical staff to the required levels will require coordinated efforts to deliver consistent training and to expand training capacity.^74^ Additional focus is needed on harmonised training for medical physicists to enable them to fulfil their legal responsibilities. Professional organisations in nuclear medicine (e.g., European Association of Nuclear Medicine) and medical physics (e.g., European Federation of Organisations for Medical Physics), as well as EU-level initiatives in radiation protection cannot shoulder these efforts alone; updates to university curricula will be required. More information on staffing is provided in the Supplementary Material pp.54.

Decision-makers should account for the time required to expand infrastructure, obtain nuclear licenses, and train staff. Delays in planning and decision-making may risk European healthcare systems falling behind those of comparable economies and may negatively affect patient outcomes, equal access to therapies and diminish Europe’s attractiveness as a hub for medical research.

## Contributors

LS, MG, UH, AP, PPa, DH, PPe conceptualization; UH original draft and editing, retrieval of incidence data, synthesis of incidence-based data; UH, DH, PPe, AP, PPa elaborating methodology; DH, PPe retrieval and analysis of prevalence data, synthesis of prevalence-based data; UH, DH, PPe, AP, CF, PK literature research and evaluation; LS, AP, MG validation of data; all authors review and final editing. DH and UH accessed and verified the data from IHME and ECIS. All authors had full access to all data and have the final responsibility for the decision to submit the manuscript for publication.

## Data sharing statement

All incidence data used for this work are reported in the Supplementary Material, and all literature sources are appropriately cited. The EXCEL files with the executable calculations will be made available by the corresponding author on demand. The IHME prevalence data accessed are subject to licensing and cannot be redistributed by the authors. Upon reasonable request, we can provide a description of the specific data extracts used.

## Declaration of interests

All authors received medical writing support from Novartis. UH, MG, RC, and PK declare no further conflict of interest. Data analysis by DH and PPe was funded by Novartis. PPe received honoraria from Roche Hellas. CF received grants and consulting fees from Advanced Accelerator Applications (a Novartis company), Bayer, Telix, and Novartis (unrelated to the present work), honoraria and travel support from Novartis, participated in a data safety monitoring board/advisory board for Telix, has a leadership/fiduciary role in NCT, and received materials or services from Recordati. PPa is employed by Novartis. AP and LS are employed by and hold stock or stock options in Novartis.
