# Supplementary material for: Estimation of Future Patient Populations Eligible for Radioligand Therapies in the EU and the UK: A Modelling Study
Source: Lancet Reg Health Eur. 2026 Jun 4;67:101733. doi: 10.1016/j.lanepe.2026.101733 (PMC13265898; doi:10.1016/j.lanepe.2026.101733)
Supplement: Supplemental Figs. S1–S7 and Tables S1–S27 [file mmc1.pdf]

## Supplementary materials

### Table of Contents


| Table of Contents | Page |
|---|---|
| Table S1: Active clinical trials with radioligand therapies in phase 2 and 3 | 1-13 |
| Table S2: Identified indications/potential future indications for RLT based on products authorised by the European Medicines Agency or in active phase 2 or phase 3 trials and actual/estimated year of authorisation | 14-15 |
| Table S3: Compilation of annual therapeutic procedures in nuclear medicine in 2020 in the EU | 16 |
| Figure S1: Data flow for estimating the number of patients biologically and clinically eligible for radioligand therapies based on prevalence and incidence data. | 17 |
| Description of Data Sources – ECIS and IHME | 18 |
| Table S4: Input parameters for the calculation of the patient pool eligible for RLT based on cancer prevalence in the EU-4 + UK and molecular target expression levels | 19-21 |
| Table S5: Evolution of patient pools eligible for radioligand therapies in France by medical indication and sub-indication from 2021 to 2033 | 22 |
| Table S6: Evolution of patient pools eligible for radioligand therapies in Germany by medical indication and sub-indication from 2021 to 2033 | 23 |
| Table S7: Evolution of patient pools eligible for radioligand therapies in Italy by medical indication and sub-indication from 2021 to 2033 | 24 |
| Table S8: Evolution of patient pools eligible for radioligand therapies in Spain by medical indication and sub-indication from 2021 to 2033 | 25 |
| Table S9: Evolution of patient pools eligible for radioligand therapies in the UK by medical indication and sub-indication from 2021 to 2033 | 26 |
| Table S10: Evolution of patient pools eligible for radioligand therapies in the EU-4 countries (France, Germany, Italy, Spain) and the UK by medical indication and sub-indication from 2021 to 2033 | 27 |
| Figure S2: Graphical presentation of the overall evolution of patient pools eligible for radioligand therapies from 2021 to 2033 | 28 |
| Methodology – Estimating the number of eligible patients from incidence figures | 29-30 |
| Table S11a: Illustration of a steady state with constant incidence numbers over time | 29 |
| Table S11b: Effect of increasing incidence over time - deviation from a steady state | 30 |
| Methodology – Calculation schemes and sensitivity analysis for incidence data | 31-32 |
| Figure S3: Calculation scheme applied in Table S12 for GEP-NETs (valid for 2022 and 2025) | 31 |
| Figure S4: Calculation scheme applied in Table S16 for the sensitivity analysis to estimate the pools size of prostate cancer patients in the mCRPC state (a) and the mHSPC state (b). Data sources are given in Table S14. | 32 |
| Figure S5: Calculation scheme applied in Table S21 for the sensitivity analysis to estimate the maximum pools size of patients with clear cell Renal Cell Carcinoma (ccRCC) that might benefit from radioligand therapy. | 32 |

Table S12: Estimate of the pool of sstr2+ GEP-NET patients eligible for radioligand therapy by authorisation criteria in the EU-27 33

Table S13: Estimate of the pool of sstr+ lung-NET patients eligible for radioligand therapy by conjectured authorisation criteria in the EU-27 34

Table S14: Patient pool estimate of sstr+ pheochromocytoma and paraganglioma patients eligible for RLT by conjectured authorisation criteria (based on clinical trials) in the EU-27 35

Table S15: Estimated overall evolution of the patient pool for patients with sstr2+ GEP-NETs, lung NETS, pheochromocytoma and paraganglioma (PPGL) eligible for radioligand therapies according to current and conjectured future authorisation criteria in the EU-27 36

Prostate Cancer – Cross checks 37

Table S16: Estimate of the evolution of the pool of PSMA+ metastatic prostate cancer patients eligible for radioligand therapies as currently authorised by EMA (2022, 2025) and for the expected extensions of future authorisations to earlier lines of treatment for the EU-27 38

Partitioning ECIS incidence numbers for haematological cancer sub-indications 39-42

Table S17a**:** Distribution of ECIS incidence data for leukaemia, non-Hodgkin's lymphoma and multiple myeloma data on the disease subtypes 39

Table S17b: Linking the incidence rates compiled by the Haematological Malignancies Research Network to the sub-types of leukaemia, Non-Hodgkins Lymphoma and multiple myeloma 40

Figure S6: Partitioning scheme of haematological ECIS incidence data into disease sub-types 41

Table S17c: Distribution of ECIS data for Leukaemia, Myeloma and Non-Hodgkin Lymphoma on disease subtypes 42

Table S18: Evolution of the estimated pool of AML patients eligible for radioligand therapy used as conditioning for allogeneic hematopoietic stem cell transplantation 43

Table S19: Evolution of the estimated pool of patients eligible for radioligand treatment of Waldenström's Macroglobulinemia 44

Table S20: Estimate of the pool of Multiple Myeloma patients eligible for radioligand therapy after at least five prior treatment regimes 45

Table S21: Estimate of the pool of patients eligible for radioligand therapy treating clear cell Renal Cell Carcinoma (ccRCC) 46

Table S22: Estimate of the pool of B7-H3+ patients eligible for neuroblastoma treatment with radioligand therapy 47

Table S23: Estimate of the pool of sst2+ malignant meningioma patients eligible for radioligand therapy and summary for brain cancer indications 48

Figure S7: Incidence-derived estimate of the evolution of the maximum patient pool eligible for radioligand therapy in the EU-27 until 2035. 49

Table S24: Scenario for the uptake of RLTs (authorised 2017 and later) in the healthcare systems of the EU-4 (Germany, France, Italy and Spain). 50

Table S25: Patients treated with $^{177}$Lu radioligand therapies in 2020 as derived from radionuclide utilisation data in Table S3 and breakdown of the uptake scenario to individual EU countries. 51

Table S26: IQVIA estimate of RLT treatment capacity in Germany, France, Italy, and Spain in 2023 52

Radionuclide production and availability 53

Challenges of staffing and training for RLT 54-56

Table S27: Additional staffing requirements for nuclear medicine healthcare professionals based on the RLT uptake scenario (see Table S24) applied to EU-27 incidence derived data in Table 1. 55

**Table S1: Active clinical trials with radioligand therapies in phase 2 and 3 (source https://www.clinicaltrials.gov/; last *a*ccessed 08 November 2025 ). Excluded were combined phase 1/2 trials.**

| NCT identifier | Brief Description | Disease Setting | Sponsor | Phase | Completion Date |
|---|---|---|---|---|---|
| **PSMA targeting** | | | | | |
| NCT05803941 | Post-marketing study to further characterize the long-term safety of $^{177}$Lu-PSMA-617 in patients with PC | PC | Novartis Pharmaceuticals | PHASE 4 | 2033-07 |
| NCT05939414 | PSMA-DC - Efficacy and safety of $^{177}$Lu-PSMA-617 in patients with PSMA+ oligometastatic prostate cancer progressing after therapy of their primary tumor, treating in early-stage PC controlling recurrent tumor and preventing progression to fatal metastatic disease while preserving quality of life by delaying treatment with ADT | oligometastatic PC | Novartis Pharmaceuticals | PHASE 3 | 2031-10 |
| NCT06320067 | STAMPEDE2 - is a systematic clinical trial comparing three new treatments with standard of care in people with mHSPC<br>1) SoC (ADT + ARSI ± docetaxel + local RT) vs stereotactic ablative body radiotherapy & SoC (ADT + ARSI ± docetaxel + local RT) + SABR<br>2) SoC (ADT + ARSI ± docetaxel ± local RT) vs $^{177}$Lu-PSMA-617 & SoC (ADT + ARSI ± docetaxel ± local RT) + $^{177}$Lu-PSMA-617<br>3) SoC (ADT + Apalutamide ± docetaxel ± local RT) vs (Niraparib + AA + P) & SoC (ADT ± docetaxel ± local RT)<br>ADT = bilateral orchidectomy, LHRH agonists or LHRH antagonists<br>ARSI = Abiraterone Acetate and Prednisolone [AA+P], Enzalutamide, Apalutamide or Darolutamide | mHSPC | University College, London | PHASE 3 | 2032-03 |
| NCT04647526 | SPLASH - Efficacy and safety of $^{177}$Lu-PNT2002 in patients with mCRPC progressing after ARPI therapy | mCRPC | POINT Biopharma (Eli Lilly) | PHASE 3 | 2028-03 |
| NCT04876651 | PROSTACT - Benefits and risks associated with the radiolabelled PSMA-targeted antibody, $^{177}$Lu DOTA rosopatamab, administered together with Standard of Care (SoC) versus the best SoC alone in patients with PSMA+ mCRPC progressing after ARPI treatment | mCRPC | Telix Pharmaceuticals (Innovations) Pty Limited | PHASE 3 | 2028-12 |
| NCT05204927 | Safety and efficacy of $^{177}$Lu-PSMA-I&T versus hormone therapy (abiraterone with prednisone or enzalutamide) in patients with mCRPC | mCRPC | Curium US LLC | PHASE 3 | 2029-02 |
| NCT04720157 | PSMAddition - Comparing efficacy $^{177}$Lu-PSMA-617 in combination with SoC (=ARPI + ADT), versus SoC alone in patients with mHSPC | mHSPC | Novartis Pharmaceuticals | PHASE 3 | 2027-02 |

| NCT identifier | Brief Description | Disease Setting | Sponsor | Phase | Completion Date |
|---|---|---|---|---|---|
| NCT04689828 | PSMAfore - Efficacy of $^{177}$Lu-PSMA-617 versus ARPI in patients with progressive mCRPC previously treated with an alternate ARPI and not exposed to a taxane-containing regimen in the CRPC or mHSPC settings | mCRPC | Novartis Pharmaceuticals | PHASE 3 | 2026-09 |
| NCT06520345 | Efficacy and safety of $^{177}$Lu-TLX591 in patients with metastatic castration-resistant prostate cancer who have progressed following treatment with Androgen Receptor Pathway Inhibitor Treatment | mCRPC | Telix Pharmaceuticals | PHASE 3 | 2030-12 |
| NCT06496581 | Standard of care +/- $^{177}$Lu-PSMA-617 in de novo mHSPC patients with poor PSA response (PEACE6-Poor Responders) | mHSPC | UNICANCER | PHASE 3 | 2039-08 |
| NCT06855277 | A phase III, open-label, multi-center, randomized study comparing AAA817+ARPI versus standard of care in adult participants with PSMA-positive metastatic castration resistant prostate cancer (AAA817: [$^{225}$Ac]Ac-PSMA-617) | mCRPC | Novartis Pharmaceuticals | PHASE 3 | 2032-11 |
| NCT06402331 | FPI-2265 ($^{225}$Ac-PSMA-I&T) for patients with PSMA-positive metastatic castration-resistant prostate cancer (mCRPC) (AlphaBreak) - Randomized, open-label, multicenter study to evaluate the safety and efficacy of FPI-2265 ($^{225}$Ac-PSMA-I&T) in mCRPC patients, previously treated with $^{177}$Lu-PSMA radioligand therapy | mCRPC | Fusion Pharmaceuticals Inc. | PHASE 2/3 | 2031-01 |
| NCT05867615 | Radiometabolic Therapy (RMT) with $^{177}$Lu PSMA in PSMA PET/CT positive advanced/metastatic tumours: a Basket Trial - Phase 2 study, single arm trial enrolling patients with a $^{68}$Ga/$^{18}$F PSMA positive PET/CT in solid tumours (e.g. of the kidney, salivary glands, the duodenum and the central and peripheral nervous system) in order to be treated with $^{177}$Lu-PSMA. | PSMA-positive solid tumours of various types | Istituto Scientifico Romagnolo per lo Studio e la cura dei Tumori | PHASE 2 | 2028-12 |
| NCT03939689 | ARROW - Safety and efficacy of $^{131}$I-1095 RLT in combination with enzalutamide compared to enzalutamide alone in chemotherapy-naive patients with PSMA+ mCRPC progressing after abiraterone therapy | mCRPC | Progenics Pharmaceuticals, Inc. | PHASE 2 | 2024-09 |
| NCT05219500 | TATCIST - Safety and efficacy of PSMA-directed Targeted Alpha Therapy with FPI-2265 ($^{225}$Ac-PSMA-I&T) for the treatment of mCRPC | mCRPC | Fusion Pharmaceuticals Inc. | PHASE 2 | 2026-07 |
| NCT04419402 | Efficacy and safety of adding $^{177}$Lu-PSMA-617 to enzalutamide versus enzalutamide alone in chemotherapy naive patients with mCRPC | mCRPC | Australian and New Zealand Urogenital and Prostate Cancer Trials Group | PHASE2 | 2025-01 |
| NCT00859781 | Efficacy of the $^{177}$Lu-labelled antibody HuJ591 ($^{177}$Lu-J591) in combination with ketoconazole and hydrocortisone against prostate cancer | PC | Weill Medical College of Cornell University | PHASE2 | 2026-05 |

| NCT identifier | Brief Description | Disease Setting | Sponsor | Phase | Completion Date |
|---|---|---|---|---|---|
| NCT05893381 | LUST - safety and efficacy of $^{177}$Lu-PSMA-I&T and Stereotactic Radiotherapy versus Stereotactic Radiotherapy alone in patients with oligometastatic prostate cancer | oligometastatic PC | Istituto Scientifico Romagnolo per lo Studio e la cura dei Tumori | PHASE 2 | 2032-04 |
| NCT05114746 | Efficacy, tolerability, safety, pharmacokinetic (PK) and dosimetry of $^{177}$Lu-PSMA-617 in participants with progressive PSMA+ mCRPC | mCRPC | Novartis Pharmaceuticals | PHASE 2 | 2028-01 |
| NCT05146973 | External Beam Therapy with theranostic radioligand therapy for oligometastatic prostate cancer (ProstACT TARGET) efficacy of a radiolabelled PSMA-targeting antibody, $^{177}$Lu-TLX591, given in combination with external beam radiation therapy (EBRT) in patients with biochemically recurrent, oligometastatic, PSMA+ PC | oligometastatic PC | Telix Pharmaceuticals (Innovations) Pty Limited | PHASE 2 | 2024-03 |
| NCT06549465 | A phase 2, open-label study evaluating dosimetry, randomized dose optimization, dose escalation and efficacy of Ac-225 Rosopatamab Tetraxetan in participants with PSMA PET-positive castration-resistant prostate cancer | mCRPC | Convergent Therapeutics | PHASE 2 | 2027-04 |
| NCT07150715 | Phase II trial comparing the use of $^{225}$Ac-PSMA-617 to $^{177}$Lu-PSMA-617, along with stereotactic body radiotherapy for the treatment of recurrent prostate cancer that has spread from the primary site to multiple other places in the body (oligometastatic). | oligometastatic recurrent prostate cancer | Jonsson Comprehensive Cancer Center | PHASE 2 | 2031-10 |
| NCT04443062 | Safety and efficacy of $^{177}$Lu-PSMA-617 in oligo-metastatic HSPC | oligometastatic HSPC | Radboud University Medical Center | PHASE 2 | 2026-01 |
| NCT05691465 | Efficacy of $^{177}$Lu-DOTATATE in patients with metastatic sstr+ prostate cancer with neuroendocrine differentiation; neuroendocrine differentiation refers to cells that have traits of both hormone-producing endocrine cells and nerve cells. | mPC with neuroendocrine differentiation | National Cancer Institute (NCI) | PHASE 2 | 2025-11 |
| NCT06288113 | RE-LuPSMA Trial: safety and efficacy of re-treatment of mCRPC with $^{177}$Lu-PSMA-617 progressing after ADT and having shown favourable response to initial treatment with $^{177}$Lu-PSMA-617. | mCRPC | Jonsson Comprehensive Cancer Center | PHASE 2 | 2027-01 |
| NCT05579184 | Safety and efficacy of $^{177}$Lu-DOTAdipep in CRPC | mCRPC | FutureChem | PHASE 2 | 2024-12 |
| NCT06259123 | Neoadjuvant PSMA-RLT in oligometastatic PC with systemic radioligand therapy $^{177}$Lu-PSMA-I&T in patients planned for radical prostatectomy | oligometastatic PC | Medical University of Vienna | PHASE 2 | 2027-06 |
| NCT05658003 | Efficacy of $^{177}$Lu-PSMA-617 vs. a change of ARPI therapy in taxane naive Chinese patients with progressing mCRPC previously treated with another ARPI as last treatment who are considered appropriate for delaying taxane-based chemotherapy | mCRPC | Novartis Pharmaceuticals | PHASE 2 | 2027-01 |
| NCT06894511 | A phase II, open-label, multi-center, randomized study comparing the combination of Lutetium ($^{177}$Lu) Vipivotide Tetraxetan (AAA617) and Androgen Receptor Pathway Inhibitor (ARPI) vs. | mCRPC | Novartis Pharmaceuticals | PHASE 2 | 2029-04 |

| NCT identifier | Brief Description | Disease Setting | Sponsor | Phase | Completion Date |
|---|---|---|---|---|---|
| | Lutetium ($^{177}$Lu) Vipivotide Tetraxetan (AAA617) in first-line treatment of patients with prostate-specific membrane antigen (PSMA)-positive progressive metastatic castration resistant prostate cancer (mCRPC) | | | | |
| NCT06084338 | Efficacy of stereotactic ablative radiotherapy combined with $^{177}$Lu-PSMA-617 RLT and cessation of castration with or without subsequent testosterone replacement in patients with mCRPC | mCRPC | VA Office of Research and Development | PHASE 2 | 2028-12 |
| NCT06200103 | De-escalation of use of $^{177}$Lu-PSMA-617 for treating patients with mCRPC utilizing a treatment pause after 5 cycles versus standard continuous 6 cycles. | mCRPC | Mayo Clinic | PHASE 2 | 2029-12 |
| NCT06145633 | Efficacy of the combination of $^{177}$Lu-PSMA-617 and Vorinostat in mCRPC patients with low expression of PSMA | mCRPC | University of Washington | PHASE 2 | 2027-12 |
| NCT06220188 | Efficacy of RLT with only 2 cycles of $^{177}$Lu-PSMA-I&T (1st 3 GBq and 2nd 6 GBq) at 6-week intervals after biochemical (PSA > 0.2 ng/ml) but not radio-morphological local recurrence following radical prostatectomy or after external beam radiotherapy. | recurrent PC | Medical University of Vienna | PHASE 2 | 2027-01 |
| NCT05150236 | EVOLUTION - Efficacy and safety $^{177}$Lu-PSMA-617 RLT therapy versus $^{177}$Lu-PSMA-617 in combination with Ipilimumab and Nivolumab in patients with mCRPC | mCRPC | Australian and New Zealand Urogenital and Prostate Cancer Trials Group | PHASE2 | 2024-12 |
| NCT05766371 | Efficacy of $^{177}$Lu-PSMA-617 RLT in combination with pembrolizumab in patients with mCRPC progressing on at least one prior ARPI (e.g., abiraterone, enzalutamide, apalutamide) | mCRPC | University of California, San Francisco | PHASE2 | 2031-05 |
| NCT04343885 | Effectiveness of $^{177}$Lu-PSMA-617 RLT in combination with docetaxel chemotherapy versus docetaxel chemotherapy alone in patients with newly diagnosed high-volume metastatic hormone-naive prostate cancer (mHNPC) | mHNPC | Peter MacCallum Cancer Centre, Australia | PHASE2 | 2026-03 |
| NCT05496959 | LUNAR - Efficacy of cancer control with $^{177}$Lu-PSMA-PNT2002 given before stereotactic body radiotherapy (SBRT) in oligorecurrent (1-5 metastasis) | oligometastatic PC | Jonsson Comprehensive Cancer Center | PHASE2 | 2033-09 |
| NCT06216249 | FLEX MRT - Comparison of efficacy and safety of a flexible $^{177}$Lu-PSMA-617 RLT dosing schedule (up to 12 cycles) versus fixed dosing schedule (6 x 7.4 GBq every 6 weeks) in patients with mCRPC | mCRPC | Jonsson Comprehensive Cancer Center | PHASE2 | 2028-02 |
| NCT05670106 | Efficacy, safety, tolerability, Pharmacokinetic(s) (PK) and dosimetry of $^{177}$Lu-PSMA-617 in addition to best supportive/best standard of care in Chinese patients with progressing PSMA+ mCRPC who received at least 1 novel ARPI and were previously treated with 1 to 2 taxane regimens | mCRPC | Novartis Pharmaceuticals | PHASE2 | 2025-11 |

| NCT identifier | Brief Description | Disease Setting | Sponsor | Phase | Completion Date |
|---|---|---|---|---|---|
| NCT05560659 | POPSTAR II -Assessment of progression free survival of stereotactic ablative radiotherapy alone versus combination with $^{177}$Lu-PSMA RLT in patients with oligometastatic PC | oligometastatic PC | Peter MacCallum Cancer Centre, Australia | PHASE2 | 2027-05 |
| NCT04663997 | Efficacy of $^{177}$Lu-PSMA-617 RLT versus standard docetaxel chemotherapy in mCRPC patients | Prostate Cancer | Canadian Cancer Trials Group | PHASE2 | 2026-12 |
| NCT06004661 | Safety and efficacy of $^{177}$Lu-PSMA-617 RLT in patients with moderate and severe renal impairment, effects on biodistribution, dosimetry and safety in patients with progressive PSMA+ mCRPC; comparison with normal renal function | mCRPC | Novartis Pharmaceuticals | PHASE2 | 2026-08 |
| NCT06909825 | Efficacy, safety and tolerability of FPI-2265 ($^{225}$Ac-PSMA-I&T) in combination with Olaparib in participants with mCRPC. | mCRPC | Fusion Pharmaceuticals | PHASE 2 | 2030-08 |
| NCT05849298 | PSMACare - Efficacy and safety of $^{177}$Lu-PSMA-617 alone vs combination with an ARPI in participants with PSMA+ CRPC and no evidence of metastasis in conventional imaging (i.e., CT/MRI and bone scans) | CRPC | Novartis Pharmaceuticals | PHASE 2 | 2030-05 |
| NCT06780670 | Open-label study comparing AAA817 ($^{225}$Ac-PSMA-617) versus standard of care in the treatment of previously treated PSMA+ mCRPC adults who have disease progressed on or after [$^{177}$Lu]Lu-PSMA targeted therapy (PSMAcTION) | mCRPC | Novartis Pharmaceuticals | PHASE 2/3 | 2033-07 |
| NCT06322576 | A Phase 2, Single-Arm, Multi-center Study of $^{177}$Lu-PSMA (177Lu-PNT2002) in Patients With PSMA-Positive Adenoid Cystic Carcinoma | Cystic adenocarcinoma | Sidney Kimmel Comprehensive Cancer Center at Johns Hopkins | PHASE 2 | 2035-02 |
| NCT06288113 | Re-treatment with $^{177}$Lu-PSMA-617 for the Treatment of Metastatic Castration-Resistant Prostate Cancer, RE-LuPSMA Trial | mCRPC | Jonsson Comprehensive Cancer Centre | PHASE 2 | 2027-01 |
| NCT06866938 | An open label, single arm phase IIb study of re-treatment With [$^{177}$Lu]Lu-PSMA in men with metastatic castration resistance prostate cancer | mCRPC | Hospices Civils de Lyon | PHASE 2 | 2030-04 |
| NCT07025512 | $^{177}$Lu-PSMA-617 in metastatic castration resistant prostate cancer (mCRPC) with bone marrow involvement and cytopenia | mCRPC | M.D. Anderson Cancer Center | PHASE 2 | 2029-06 |
| NCT05766371 | A phase 2 study of pembrolizumab plus $^{177}$Lu-PSMA-617 in patients with metastatic castration resistant prostate cancer | mCRPC | University of California, San Francisco | PHASE 2 | 2031-05 |
| NCT06738303 | Carboplatin and Cabazitaxel versus $^{177}$Lu-PSMA-617 in patients with aggressive, metastatic castrate-resistant prostate cancer (CATCH-177) | mCRPC | Case Comprehensive Cancer Center | PHASE 2 | 2026-12 |
| NCT07047118 | A phase II, randomized, open-label, multi-center study of JSB462 (Luxdegalutamide) in combination with Lutetium ($^{177}$Lu) Vipivotide Tetraxetan in adult male patients with PSMA-positive metastatic castration resistant prostate cancer (mCRPC) | mCRPC | Novartis Pharmaceuticals | PHASE 2 | 2028-11 |

| NCT identifier | Brief Description | Disease Setting | Sponsor | Phase | Completion Date |
|---|---|---|---|---|---|
| NCT06449781 | $^{177}$Lu-PSMA as a systemic adjuvant treatment in patients with high- and very high-risk prostate cancer after radical treatment using locoregional teleradiotherapy and hormone therapy | high-risk locoregional prostate cancer | Maria Sklodowska-Curie National Research Institute of Oncology | PHASE 2 | 2030-11 |
| NCT06798558 | Phase II study of neoadjuvant Lu-177-PSMA-617 in patients with high risk localized prostate cancer undergoing radical prostatectomy | high risk localized prostate cancer | Hackensack Meridian Health | PHASE 2 | 2029-03 |
| NCT07150715 | Alpha-emitting radionuclide or beta-emitting radionuclide combined with metastasis-directed stereotactic body radiotherapy for oligorecurrent prostate adenocarcinoma (ANDROMEDA) | oligorecurrent prostate adenocarcinoma | Jonsson Comprehensive Cancer Center | PHASE 2 | 2031-10 |
| **somatostatin receptor (sstr) targeting** | | | | | |
| NCT06016855 | Efficacy of surgical debulking prior to $^{177}$Lu-DOTATATE therapy in well differentiated sstr+ GEP-NETs with grade 1 or 2 that have spread to the liver (hepatic metastasis) | metastatic GEP-NETs G1 and G2 | Vanderbilt-Ingram Cancer Center | PHASE4 | 2028-05 |
| NCT05459844 | Comparing efficacy of $^{177}$Lu-DOTATATE injection to high dose (60 mg) octreotide LAR in patients with unresectable or metastatic, progressive, well differentiated (G1 and G2), somatostatin receptor positive GEP-NETs | NETs | Sinotau Pharmaceutical Group | PHASE 3 | 2028-12 |
| NCT04919226 | COMPOSE - Efficacy, safety and patient-reported outcomes of $^{177}$Lu-Edotreotide as 1st or 2nd line of treatment versus best standard of care in well-differentiated, aggressive, somatostatin receptor-positive (SSTR+) grade-2 and grade-3 GEP-NETs | Neuroendocrine Tumors | ITM Solucin GmbH | PHASE 3 | 2027-09 |
| NCT06784752 | A phase III multi-center, randomized, open-label study to evaluate the efficacy and safety of [$^{177}$Lu]Lu-DOTA-TATE in patients newly diagnosed with Grade 1 and Grade 2 (Ki-67 <10%) advanced GEP-NET with high disease burden (NETTER-3) | advanced GEP-NETs | Novartis Pharmaceuticals | PHASE 3 | 2034-01 |
| NCT05918302 | LEVEL - Efficacy and safety of $^{177}$Lu-edotreotide radiotherapy compared to everolimus in somatostatin receptor positive, moderately differentiated NETs of the lung and thymus | NETs of lung and thymus | Grupo Espanol de Tumores Neuroendocrinos | PHASE 3 | 2028-07 |
| NCT05884255 | Efficacy and safety of $^{177}$Lu-Oxodotreotide plus high-dose long-acting octreotide vs high-dose long-acting octreotide alone in patients with sst2-positive advanced GEP-NETs | advanced GEP-NETs | Jiangsu HengRui Medicine Co., Ltd. | PHASE 3 | 2030-10 |
| NCT05387603 | START-NET - Efficacy and safety of personalized systemic targeted adaptive peptide receptor radiotherapy (PRRT) of neuroendocrine tumors versus non-personalized PRRT (4 cycles of 7.5 GBq $^{177}$Lu-DOTATOC). Patients with positive $^{68}$Ga-DOTA-PET and negative FDG-PET will receive dosimetry-based PRRT only (dTOC); patients with $^{68}$Ga-DOTA- and $^{18}$F-FDG-PET-positive NET will receive a combination of capecitabine and dosimetry-based PRRT (CAP-dTOC). | NETs | Lund University Hospital | PHASE 3 | 2026-10 |

| NCT identifier | Brief Description | Disease Setting | Sponsor | Phase | Completion Date |
|---|---|---|---|---|---|
| NCT03049189 | COMPETE - Efficacy and Safety of $^{177}$Lu-edotreotide Peptide Receptor Radionuclide Therapy (PRRT) in GEP-NET Patients compared to targeted molecular therapy with Everolimus in patients with inoperable, progressive, somatostatin receptor-positive (SSTR+) GEP-NETs | GEP-NETs | ITM Solucin GmbH | PHASE 3 | 2029-11 |
| NCT03972488 | NETTER-2 - Efficacy and safety of $^{177}$Lu-DOTATATE in combination with long-acting octreotide in patients with high proliferation rate GEP-NETs (G2 and G3) when given as a first line treatment compared to treatment with high dose (60 mg) long-acting octreotide | GEP-NETs | Advanced Accelerator Applications | PHASE 3 | 2027-10 |
| NCT05477576 | ACTION-1 - Safety, efficacy and pharmacokinetics of RYZ101 ($^{225}$Ac-DOTATATE) compared with SOC (investigator's choice of between everolimus, sunitinib, octreotide, or lanreotide) in patients with inoperable sstr2+, well-differentiated gastroenteropancreatic neuroendocrine tumors (GEP-NETs) progressing after therapy with a $^{177}$Lu-labelled somatostatin analogue, such as $^{177}$Lu-DOTATATE or $^{177}$Lu-DOTATOC | inoperable sstr+, well-differentiated GEP-NETs | RayzeBio, Inc. | PHASE 3 | 2028-07 |
| NCT05153772 | ALPHAMEDIX02 - Targeted Alpha-emitter Therapy with $^{212}$Pb-DOTAMTATE in sstr2+ neuroendocrine tumor patients with or without prior peptide receptor radionuclide therapy | sstr2+ neuroendocrine tumors | Orano Med LLC | PHASE 2 | 2028-10 |
| NCT05568017 | Neoadjuvant peptide receptor radiotherapy with $^{90}$Y-DOTATOC in pNET patients with unresectable or borderline resectable pNET. Since surgery is the only curative option for patients with GEP-NETs, the study will evaluate the efficacy of $^{90}$Y-DOTATOC for disease regression to enable surgical resection and rate of R0 surgery. | Pancreatic Neuroendocrine Tumor | European Institute of Oncology | PHASE 2 | 2024-11 |
| NCT03457948 | Efficacy of the monoclonal antibody pembrolizumab in combination with (i) transarterial embolization, (ii) radioembolization with $^{90}$Y microspheres and (iii)with targeted peptide receptor radiotherapy with $^{177}$Lu-DOTATATE in patients with well-differentiated neuroendocrine tumors with liver metastasis. | liver metastasis, NETs | Nicholas Fidelman, MD | PHASE 2 | 2026-05 |
| NCT04339036 | This is a Phase 2 Evaluation of hepatic-progression free survival of patients with Grade 2 liver-dominant, unresectable NET metastases undergoing combination therapy with CapTem (Capecitabine-Temozolomide) and $^{90}$Y radioembolization. | NETs Grade 2 | Abramson Cancer Center at Penn Medicine | PHASE 2 | 2026-05 |
| NCT06395402 | LUMOD-ID - Evaluation of outcome of individualized dosimetry-based prescribing of $^{177}$Lu-DOTATATE versus administration of standard dose (4 x 7.4 GBq in 4 weeks distance) in patients with unresectable neuroendocrine tumors | Neuroendocrine Tumors | University of Iowa | PHASE 2 | 2029-12 |
| NCT04917484 | DOBATOC - Dosimetry based peptide receptor radiotherapy with 4 individualized doses of $^{177}$Lu-DOTATATE versus standard dose therapy with 4x7.4 GBq in NEN patients. | Neuroendocrine Neoplasm | Tine Gregersen, MD | PHASE 2 | 2026-12 |
| NCT02236910 | Open Label Registry Study of Lutetium-177 (DOTA0, TYR3) Octreotate ($^{177}$Lu-DOTATATE) treatment in patients with sstr2+ tumours. | Neuroendocrine Carcinoma | Lawson Health Research Institute | PHASE 2 | 2025-12 |

| NCT identifier | Brief Description | Disease Setting | Sponsor | Phase | Completion Date |
|---|---|---|---|---|---|
| NCT05691465 | Safety and Effectiveness of $^{177}$Lu-DOTATATE treatment of patients with mPC with neuroendocrine differentiation. | mPC with neuroendocrine differentiation | National Cancer Institute (NCI) | PHASE 2 | 2025-11 |
| NCT05987176 | NELMAS - Efficacy of adjuvant treatment with $^{177}$Lu-DOTATATE after resection of neuroendocrine liver metastases versus to best supportive care in patients after resection of neuroendocrine liver metastases | NET with liver metastasis | Imperial College London | PHASE 2 | 2029-03 |
| NCT06045260 | LUFOR - Efficacy and safety of peptide receptor radionuclide therapy with $^{177}$Lu-DOTATOC ($^{177}$Lu-edotreotide) in neuroendocrine neoplasia not originating from the digestive tract for which $^{177}$Lu-DOTATATE is currently not authorised: bronchopulmonary, ovarian, renal NETs, neuroendocrine carcinomas, pheochromocytomas, paragangliomas, meningiomas, and other SSTR+ malignancies | non GEP-NETs, sstr2+ tumours, paraganglioma, pheochromo-cytoma | Istituto Scientifico Romagnolo per lo Studio e la cura dei Tumori | PHASE 2 | 2027-01 |
| NCT05894486 | Efficacy and safety of $^{177}$Lu-DOTATATE as first-line treatment of patients with locally advanced or metastatic, sstr2+ G2 or G3 GEP-NETs | Neuroendocrine Tumors | Peking University | PHASE 2 | 2026-06 |
| NCT04711135 | Safety and dosimetry of $^{177}$Lu-DOTATATE in patients with sstr+ GEP-NETs, Pheochromocytoma and Paragangliomas (NETTER-P) | GEP-NETs, pheochromo-cytoma, paraganglioma | Advanced Accelerator Applications | PHASE 2 | 2034-05 |
| NCT03206060 | Safety and tolerability of $^{177}$Lu-DOTATATE therapy for inoperable pheochromocytoma and paraganglioma | pheochromocytoma, paraganglioma | National Cancer Institute (NCI) | PHASE 2 | 2027-01 |
| NCT05773274 | NET RETREAT - Comparing retreatment of $^{177}$Lu-DOTATATE PRRT versus everolimus in patients with metastatic unresectable midgut neuroendocrine tumors | metastatic midgut NETs | National Cancer Institute (NCI) | PHASE 2 | 2026-04 |
| NCT04954820 | ReLUTH - Efficacy of two additional cycles of $^{177}$Lu-DOTATATE versus active surveillance in patients already retreated with two cycles $^{177}$Lu-DOTATATE for a new progression of intestinal neuroendocrine tumor and who previously received the 4 cycles of treatment with a clinical benefit | progressive intestinal well differentiated NETs | Institut du Cancer de Montpellier - Val d'Aurelle | PHASE 2 | 2031-10 |
| NCT05724108 | Effectiveness of triapine added to $^{177}$Lu-DOTATATE compared to $^{177}$Lu-DOTATATE alone for progressing metastatic neuroendocrine tumors | metastatic neuroendocrine tumor | National Cancer Institute (NCI) | PHASE 2 | 2025-11 |
| NCT04525638 | Efficacy of the combination of $^{177}$Lu-DOTATATE and Nivolumab in grade 3 well-differentiated neuroendocrine tumours (NET) or poorly differentiated neuroendocrine carcinomas (NEC) | NETs, NECs | Fundación de Investigación HM | PHASE 2 | 2024-09 |
| NCT02489604 | LUNET - Comparison of two different dosage regimes (total activity of 25.9 GBq and of 18.5 GBq) for the peptide receptor radionuclide therapy (PRRT) with $^{177}$Lu-DOTATATE in patients with advanced GEP-NETs G1-G2 with progressive disease, which are sstr-positive and FDG-negative | GEP-NETs, pheochromocytoma, paraganglioma | Istituto Scientifico Romagnolo per lo Studio e la cura dei Tumori | PHASE 2 | 2024-01 |

| NCT identifier | Brief Description | Disease Setting | Sponsor | Phase | Completion Date |
|---|---|---|---|---|---|
| NCT04665739 | Efficacy of $^{177}$Lu-DOTATATE in patients with somatostatin receptor positive advanced disseminated bronchial NETs compared to the standard treatment with everolimus | Advanced, metastatic lung NETs, G1, G2 | National Cancer Institute (NCI) | PHASE 2 | 2033-01 |
| NCT04903899 | LuDO-N - Efficacy, dosimetry and toxicity of $^{177}$Lu-DOTATATE in children with primary refractory or relapsed high-risk neuroblastoma and correlation to SSTR-2 expression | neuroblastoma, recurrent neuroblastoma | Jakob Stenman | PHASE 2 | 2031-05 |
| NCT02754297 | P-PRRT - Efficacy and toxicity of personalized peptide receptor radiotherapy of NETs with $^{177}$Lu-DOTATATE to maximize absorbed radiation dose to tumor, while limiting that to healthy organs; determination of a absorbed radiation dose-response (tumour survival, toxicity) relationship | NETs | CHU de Quebec-Universite Laval | PHASE 2 | 2029-04 |
| NCT06121271 | Prospective evaluation of safety and efficacy of $^{177}$Lu-DOTATATE in unresectable or metastatic, somatostatin receptor-expressing neuroendocrine tumours (NET) in currently unlicensed indications (eg, bronchial and thymic NETs, paraganglioma, phaeochromocytoma, medullary thyroid carcinoma, and those requiring repetition of treatment with 2 further cycles of $^{177}$Lu-DOTATATE | bronchial and thymic NETs, paraganglioma, phaeochromo-cytoma, medullary thyroid carcinoma | University College, London | PHASE 2 | 2027-11 |
| NCT01876771 | Safety and efficacy of $^{177}$Lu-DOTATATE therapy in sstr+ neuroendocrine tumours and assessment of QoL and survival | NETs | AHS Cancer Control Alberta | PHASE 2 | 2042-12 |
| NCT04837885 | LUTARTERIAL - Safety and efficacy of intra-arterial hepatic (IAH) infusion versus intravenous administration of radiolabelled somatostatin analogues in GEP-NET patients with dominant liver metastases. | GEP-NETs | University Hospital, Bordeaux | PHASE 2 | 2027-03 |
| NCT05247905 | Comparing the efficacy of the combination of capecitabine and temozolomide versus $^{177}$Lu-DOTATATE in patients with advanced, unresectable, disseminated pancreatic neuroendocrine tumors | metastatic, unresectable pancreatic NETs | Alliance for Clinical Trials in Oncology | PHASE 2 | 2033-10 |
| NCT04529044 | Efficacy of $^{177}$Lu-DOTATATE in treating patients with metastatic or recurrent sstr+ breast cancer at stage IV | recurrent or metastatic breast cancer | OHSU Knight Cancer Institute | PHASE 2 | 2026-12 |
| NCT04903899 | LuDO-N - $^{177}$Lu-DOTATATE in Children With Primary Refractory or Relapsed High-risk Neuroblastoma (LuDO-N) | Neuroblastoma | Jakob Stenman | PHASE 2 | 2031-05 |
| NCT05987176 | An international multicentre randomized nnblinded phase II study comparing adjuvant treatment with $^{177}$Lu-DOTATATE (Lutathera®) to best supportive care in patients after resection of neuroendocrine liver metastases | resected neuroendocrine liver metastases | Imperial College London | PHASE 2 | 2029-03 |
| NCT05583708 | iPRRT - Efficacy of Peptide Receptor Radionuclide Therapy with $^{177}$Lu-DOTATATE in combination with pembrolizumab immunotherapy for patients with Merkel Cell Cancer | Merkel Cell Cancer | Weill Medical College of Cornell University | PHASE 2 | 2026-09 |

| NCT identifier | Brief Description | Disease Setting | Sponsor | Phase | Completion Date |
|---|---|---|---|---|---|
| NCT06326190 | LUMEN-1 - $^{177}$Lu-DOTATATE for Recurrent Meningioma - Efficacy of $^{177}$Lu-DOTATATE therapy in refractory meningioma which is sstr+ in PET-based somatostatin receptor imaging making use of the proven efficacy of SSTR-targeting radioligand therapy with $^{177}$Lu-DOTATATE in neuroendocrine tumors overexpressing sstr2 | Recurrent Meningioma | EORTC - European Organisation for Research and Treatment of Cancer | PHASE 2 | 2028-12 |
| **CAIX and PSMA targeting in clear cell Renal Cell Carcinoma** | | | | | |
| NCT05239533 | Efficacy and safety of nivolumab in combination with $^{177}$Lu-girentuximab for advanced clear cell renal cell carcinoma (ccRCC) that has the CAIX protein. | clear cell Renal Cell Carcinoma (ccRCC) | Memorial Sloan Kettering Cancer Center | PHASE 2 | 2026-03 |
| NCT06783348 | Phase II trial evaluating the efficacy of 177Lutetium-PSMA-617 treatment in patients with metastatic clear cell Renal Carcinoma Cell with progressive disease on first-line or second-line Systemic Treatment | ccRCC | EORTC - European Organisation for Research and Treatment of Cancer | PHASE 2 | 2029-01 |
| NCT06964958 | LASER - a phase 2 trial of $^{177}$Lu-PSMA-617 as systemic therapy for Renal Cell Carcinoma | RCC | Dana-Farber Cancer Institute | PHASE 2 | 2028-12 |
| NCT06959433 | Safety and Efficacy of Lu-177 PSMA Treatment in Metastatic Clear Cell Renal Carcinoma | ccRCC | Ankara University | PHASE 3 | 2028-08 |
| NCT07197580 | A Phase 3, randomized, multi-center, open-label study to compare $^{177}$Lu-TLX250 (Lutetium ($^{177}$Lu) Girentuximab Tetraxetan) with the investigator's choice of a single agent therapy in participants with carbonic anhydrase 9 (CAIX) expressing, advanced relapsed or recurrent clear cell Renal Cell Carcinoma (ccRCC) | ccRCC | Telix Pharmaceuticals (Innovations) Pty Limited | PHASE 3 | 2029-02 |
| **Antibody targeting of haematological diseases** | | | | | |
| NCT02665065 | SIERRA - Efficacy of Iomab-B ($^{131}$I-apamistamab) in conjunction with a Reduced Intensity Conditioning (RIC) regimen and protocol-specified allogeneic hematopoietic stem cell transplant (HCT) versus conventional care in older subjects with active, relapsed or refractory Acute Myeloid Leukaemia (AML). Iomab-B ($^{131}$I-apamistamab) is an anti-CD45 radioimmunoconjugate) | AML | Actinium Pharmaceuticals | PHASE 3 | 2026-12 |
| NCT00006721 | Efficacy, safety and toxicity of CHOP (cyclophosphamide, doxorubicin, vincristine, and prednisone) only, and CHOP plus rituximab versus CHOP plus $^{131}$I-labeled monoclonal anti-B1 antibody (tositumomab) for treatment of newly diagnosed follicular Non-Hodgkin's Lymphomas (NHL) | follicular NHL | SWOG Cancer Research Network | PHASE 3 | 2025-12 |
| NCT01827605 | Comparing consolidation with $^{90}$Y-labeled Ibritumomab Tiuxetan (Zevalin®) radioimmunotherapy versus Autologous Stem Cell Transplantation (ASCT) in patients with relapsed/refractory follicular lymphoma (FL) | relapsed or refractory follicular lymphoma | Fondazione Italiana Linfomi - ETS | PHASE 3 | 2024-01 |

| NCT identifier | Brief Description | Disease Setting | Sponsor | Phase | Completion Date |
|---|---|---|---|---|---|
| NCT02952508 | CLOVER-1 - Study of Iopofosine $^{131}$I (CLR 131) in Select B-Cell Malignancies (CLOVER-1) and Pivotal Expansion in Waldenstrom Macroglobulinemia (CLOVER-WaM) Evaluation of iopofosine $^{131}$I (CLR 131) in patients with select B-cell malignancies (multiple myeloma( MM), indolent chronic lymphocytic leukemia (CLL)/small lymphocytic lymphoma (SLL), lymphoplasmacytic lymphoma (LPL)/Waldenstrom Macroglobulinemia (WM), marginal zone lymphoma (MZL), mantle cell lymphoma (MCL), diffuse large B-cell lymphoma (DLBCL), and central nervous system lymphoma (CNSL) who have been previously treated with standard therapy for their underlying malignancy. Part B (CLOVER-WaM) is a pivotal efficacy study evaluating IV administration of iopofosine $^{131}$I in patients with WM that have received at least two prior lines of therapy. | WM, MM, CLL, SLL, LL, MZL, MCL, DLBCL, CNSL | Cellectar Biosciences, Inc. | PHASE 2 | 2026-12 |
| NCT04871607 | Efficacy of $^{90}$Y labelled anti-CD25 monoclonal antibody combined with BEAM chemotherapy conditioning for the Treatment of primary refractory or relapsed Hodgkin Lymphoma. "BEAM" stands for the four drugs (i) Carmustine (BCNU), (ii) Etoposide, (iii) Cytarabine (Ara-C) and (iv) Melphalan | recurrent or refractory Hodgkin Lymphoma | City of Hope Medical Center | PHASE 2 | 2027-10 |
| NCT04856215 | $^{90}$Y-labelled Anti-CD66 ab in childhood high-risk leukaemia – Bone marrow ablation with radioimmunotherapy to prepare patients for bone marrow transplantation | Leukaemia | Great Ormond Street Hospital for Children NHS Foundation Trust | PHASE 2 | 2025-09 |
| NCT00135200 | Clinical trial of consolidation treatment with $^{131}$I-tositumomab (known by the tradename Bexxar®, targeting CD20 on various types of B-cells including lymphoma cells, and some myeloma cells) for Multiple Myeloma - Approximately 20-25% of patients with multiple myeloma express CD20 on the surface of their tumor cells. Bexxar will be used after patients complete a course of chemotherapy and have residual myeloma cells left in their body | Multiple Myeloma | University of Michigan Rogel Cancer Center | PHASE 2 | 2024-09 |
| **Hepatocellular carcinoma (HCC) and liver metastasis treatment with $^{90}$Y- or $^{166}$Ho-loaded microspheres** (not strictly RLT) | | | | | |
| NCT06040099 | Efficacy of durvalumab and bevacizumab following transarterial radioembolization (TARE) using $^{90}$Y-Glass Microspheres (TheraSphere™) in unresectable hepatocellular carcinoma (HCC) amenable to locoregional therapy | HCC | AstraZeneca | PHASE 2 | 2026-10 |
| NCT06178198 | RESCUE - Efficacy and safety of $^{90}$Y ablative radioembolization (Radiation Major Hepatectomy) for unifocal large hepatocellular carcinoma as a potential curative approach in patients with large hepatocellular carcinoma (greater than 8 cm) who maintain good liver function utilizing $^{90}$Y resin microspheres | HCC | Seoul National University Hospital | PHASE 2 | 2026-11 |
| NCT06332079 | Efficacy of $^{166}$Ho-TARE followed by maintenance therapy with fluoropyrimidine and anti-EGFR or bevacizumab in liver limited unresectable colorectal cancer patients after first-line chemotherapy | liver metastasis | Gruppo Oncologico del Nord-Ovest | PHASE 2 | 2027-02 |

| NCT identifier | Brief Description | Disease Setting | Sponsor | Phase | Completion Date |
|---|---|---|---|---|---|
| **Brain tumours and tumours of the central nervous system (CNS)** | | | | | |
| NCT06955169 | MOMENTUM-1: A multicenter, randomized, open-label, phase II study of [$^{177}$Lu]LU-DOTATATE in adults with progressive intracranial grade 1-3 Meningioma | progressive intracranial grade 1-3 Meningioma | RTOG Foundation, Inc. | PHASE 2 | 2030-08 |
| NCT04903899 | A Phase II Trial of 177Lutetium-DOTATATE in children with primary refractory or relapsed high-risk neuroblastoma | High-risk Neuroblastoma | Jakob Stenman; Adv. Acc. Appl.; Novartis | PHASE 2 | 2031-05 |
| NCT03971461 | Efficacy of $^{177}$Lu-DOTATATE in patients with progressive WHO I-III or residual high-risk Ga-DOTATATE PET-MRI positive meningioma | high-risk meningioma | NYU Langone Health | PHASE 2 | 2027-01 |
| NCT04744366 | Efficacy of addition of cRIT $^{131}$I-omburtamab to irinotecan, temozolomide, and bevacizumab for patients with recurrent medulloblastoma. A feasibility cohort is included to assess the feasibility of incorporating cRIT $^{131}$I-omburtamab for patients with recurrent ependymoma (pediatric patients with recurrent tumors) | recurrent medulloblastoma | Pediatric Brain Tumor Consortium | PHASE 2 | 2030-10 |
| NCT04082520 | Safety and efficacy of $^{177}$Lu-Dotatate in patients with inoperable, progressive meningioma after External Beam Radiation Therapy EBRT); WHO Grade I and Cohort WHO II/III cohorts will be evaluated | inoperable, progressive meningioma | Mayo Clinic | PHASE 2 | 2031-01 |
| NCT06126588 | Efficacy of everolimus in combination with $^{177}$Lu-DOTATATE in patients with grades 2 and 3 refractory meningioma after surgery and external beam radiotherapy; (grade 1/non-aggressive and high risk of aggressive behaviour (grade 2/atypical and 3/anaplastic)) | high-risk meningioma | Central Hospital, Nancy, France | PHASE 2 | 2028-05 |
| **Other (solid) tumours** | | | | | |
| NCT04022213 | Combination of $^{131}$I-omburtamab radioimmunotherapy alone versus $^{131}$I-omburtamab radioimmunotherapy in combination with and Intensity Modulated external beam Radiotherapy (IMRT) for desmoplastic small round cell tumor (DSRCT) and other solid tumors in the peritoneum | solid tumors in the peritoneum | Memorial Sloan Kettering Cancer Center | PHASE 2 | 2026-07 |
| NCT04529044 | A Phase II Pilot Study of (Lutetium ($^{177}$Lu)-DOTATATE in Patients with Metastatic Breast Cancer | stage IV or recurrent sstr2+ breast cancer | OHSU Knight Cancer Institute | PHASE 2 | 2026-12 |
| NCT06880757 | $^{177}$Lu-FAP-2286 Treatment in Urethelial Neoplasms: Utility and Safety as a Novel Treatment | Urethelial Neoplasms | Ankara University | PHASE 2 | 2028-06 |
| NCT06607692 | Study in children and adolescents of $^{177}$Lu-DOTATATE (Lutathera®) combined with the PARP inhibitor olaparib for treatment of recurrent or relapsed solid tumours expressing somatostatin receptors (SSTR) (LuPARPed) | Sstr+ solid tumours in children | Fundación de investigación HM | PHASE 2 | 2029-12 |

| NCT identifier | Brief Description | Disease Setting | Sponsor | Phase | Completion Date |
|---|---|---|---|---|---|
| NCT05198479 | Safety and efficacy of $^{177}$Lu-DOTA0-Tyr3-Octreotate in metastatic nasopharyngeal cancer in patients that have failed 2 or more lines of therapy or exhausted standard therapy and are sstr2+ on $^{68}$Ga-DOTATATE PET imaging | Nasopharyngeal Cancer | National Cancer Centre, Singapore | PHASE 2 | 2025-09 |
| **Treatment of bone metastasis / bone pain palliation (only phase 4)** | | | | | |
| NCT03432949 | TRANCE - Efficacy of $^{223}RaCl_2$ combined with dexamethasone as first-line therapy in patients with m+CRPC; > 90% of patients with mCRPC have evidence of bone metastases which is a major cause of death, disability, and decreased quality of life | bone metastatic mCRPC | University Health Network, Toronto | PHASE 4 | 2027-07 |
| NCT04597125 | Investigation of $^{223}RaCl_2$ (Xofigo) versus new antihormonal therapy (NAH) in patients with bone metastatic PC; NAH drugs will be either abiraterone acetate (Zytiga) (plus prednisone/prednisolone) or enzalutamide (Xtandi) | bone metastatic mCRPC | BAYER | PHASE 4 | 2026-10 |

AML - Acute Myeloid Leukaemia; ccRCC - clear cell Renal Cell Carcinoma; CLL - Chronic Lymphocytic Leukaemia; CNSL – Central Nevous System Lymphoma; DLBCL - Diffuse Large B-Cell Lymphoma; FL - Follicular Lymphoma; GEP-NET – gastroenteropancreatic Neuroendocrine Tumour; HCC - Hepatocellular Carcinoma; HSPC – Hormone-Sensitive Prostate Cancer; LPL - Lymphoplasmacytic Lymphoma; mCRPC – metastatic Castration-Resistant Prostate Cancer; mHSPC – metastatic Hormone-Sensitive Prostate Cancer; MCL - Mantle Cell Lymphoma; MM - Multiple Myeloma; MZL - Marginal Zone Lymphoma; NEC – Neuroendocrine Cancer; NET - Neuroendocrine Tumours; PC – Prostate Cancer; SLL - Small Lymphocytic Lymphoma; sstr2 – somatostatine receptor type 2; WM - Waldenström Macroglobulinemia

**Table S2: Identified indications/potential future indications for RLT based on products authorised by the European Medicines Agency or in active phase 2 or phase 3 trials and actual/estimated year of authorisation**

| Indication (sub-indication), by cancer type | Product name(s) | Target marker | Trial phase | Authorisation / earliest estimated authorisation by EMA | Trial ID |
|---|---|---|---|---|---|
| ***Neuroendocrine tumors*** | | | | | |
| General (metastatic, 2L) | $^{212}$Pb-DOTAMTATE (AlphaMedix) | SSTR2 | Phase 2 | 2031 | NCT05153772 |
| | $^{177}$Lu-oxodotreotide (PNT2003, generic LUTATHERA) | SSTR2 | Phase 3 | 2029 | Filed no ID yet |
| GEP-NET (G1–G2, metastatic, 2L) | $^{177}$Lu-oxodotreotide (LUTATHERA) | SSTR2 | Approved | 2017 | NA |
| | $^{177}$Lu-edotreotide (ITM-11) | SSTR2 | Phase 3 | 2028 | NCT03049189 |
| | $^{225}$Ac-oxodotreotide (RYZ 101) | SSTR2 | Phase 3 | 2027 | NCT05477576 |
| | $^{177}$Lu-oxodotreotide | SSTR2 | Phase 2/3 | 2029 | NCT06398444 |
| | $^{177}$Lu-oxodotreotide | SSTR2 | Phase 3 | 2031 | NCT05884255 |
| GEP-NET (G2–G3, metastatic) | $^{177}$Lu-edotreotide (ITM-11) | SSTR2 | Phase 3 | 2027 | NCT04919226 |
| | $^{177}$Lu-oxodotreotide (LUTATHERA) | SSTR2 | Phase 3 | 2026 | NCT03972488 |
| GEP-NET and PPGL | $^{177}$Lu-oxodotreotide (LUTATHERA) | SSTR2 | Phase 2 | 2030 | NCT04711135 |
| ***Prostate Cancer*** | | | | | |
| mCRPC (3L) | $^{177}$Lu-vipivotide tetraxetan (Pluvicto)[a] | PSMA | Approved | 2022 | NA |
| mCRPC (2L) | $^{177}$Lu-vipivotide tetraxetan (Pluvicto)[a] | PSMA | Phase 3 | 2027 | NCT04689828 |
| | $^{177}$Lu-zadavotide guraxetan ($^{177}$Lu-PSMA-I&T, PNT2002) | PSMA | Phase 3 | 2029 | NCT05204927 |
| | $^{177}$Lu-zadavotide guraxetan ($^{177}$Lu-PSMA-I&T, PNT2002) | PSMA | Phase 3 | 2028 | NCT04647526 |
| | $^{225}$Ac-zadavotide guraxetan ($^{225}$Ac-PSMA-I&T) | PSMA | Phase 2 | 2030 | NCT05219500 |
| | | PSMA | Phase 2/3 | 2030 | NCT06402331 |
| | $^{225}$Ac-vipivotide tetraxetan | PSMA | Phase 2/3 | 2033 | NCT06780670 |
| | $^{225}$Ac-vipivotide tetraxetan + ARPI | PSMA | Phase 3 | 2032 | NCT06855277 |
| | $^{131}$I-MIP-1095 (I-131-1095) | PSMA | Phase 2 | 2027 | NCT03939689 |
| | $^{177}$Lu-ludotadipep | PSMA | Phase 2 | 2030 | NCT05579184 |
| | $^{177}$Lu-DOTA-rosopatamab (TLX591) | PSMA | Phase 3 | 2028 | NCT06520345 |
| | | PSMA | Phase 3 | 2028 | NCT04876651 |
| | $^{225}$Ac Rosopatamab tetraxetan | PSMA | Phase 2 | 2032 | NCT06549465 |
| mHSPC | $^{177}$Lu vipivotide tetraxetan (Pluvicto) | PSMA | Phase 3 | 2029 | NCT04720157 |

| | | | | | |
|---|---|---|---|---|---|
| OMPC | $^{177}$Lu vipivotide tetraxetan (Pluvicto) | PSMA | Phase 3 | 2030 | NCT05939414 |
| | $^{177}$Lu-DOTA-rosopatamab (TLX591) | PSMA | Phase 2 | 2031 | NCT05146973 |
| **Haematological malignancies** | | | | | |
| AML | $^{131}$I-BC8-Iomab-B (Iomab-B) | CD45 | Phase 3 | 2030 | NCT02665065 |
| LPL/WM | $^{131}$-Iopofosine (CLR 131) | NA | Phase 2 | 2030 | NCT02952508 |
| MM (6L) | $^{131}$I-Iopofosine (CLR 131) | NA | Phase 2 | 2030 | NCT02952508 |
| ***Kidney cancer*** | | | | | |
| ccRCC (metastatic, 2L) | $^{177}$Lu-DOTA-girentuximab (LX250) | CAIX | Phase 2 | 2031 | NCT05239533 |
| ***Brain and CNS tumors*** | | | | | |
| Malignant meningioma | $^{177}$Lu-oxodotreotide (LUTATHERA)<br>$^{177}$Lu-oxodotreotide (LUTATHERA) | SSTR2<br>SSTR2 | Phase 2b<br>Phase 2 | 2030<br>2030 | NCT06126588<br>NCT03971461 |
| Neuroblastoma / CNS (children) | $^{131}$I-omburtamab | B7-H3/CD276 | Phase 2/3 | 2027 | EudraCT 2017-001828-22 |

Source: NIH National Library of Medicine (https://clinicaltrials.gov/) or EU Clinical Trials Register (Clinical Trials Register; accessed June 2025)

[a] $^{177}$Lu-PSMA-617 is authorized in the EU for the treatment of adult patients with PSMA-positive mCRPC who have been treated with an ARPI and taxane-based chemotherapy and is defined as 3L for the purposes of this analysis

1L, first-line; 2L, second-line; 3L, third-line; 6L, sixth-line; $^{225}$Ac, Actinium-225; $^{131}$I, Iodine-131; $^{177}$Lu, Lutetium-177; $^{223}$Ra, Radium-223; AML, acute myeloid leukemia; ARPI, androgen receptor pathway inhibitor; CAIX, carbonic anhydrase IX; ccRCC, clear cell renal cell carcinoma; CNS, central nervous system; FL, follicular lymphoma; G, grade; GEP-NET, gastroenteropancreatic neuroendocrine tumor; LPL, lymphoplasmacytic lymphoma; PSMA, prostate specific membrane antigen; mCRPC, metastatic castration-resistant prostate cancer; mHSPC, metastatic hormone-sensitive prostate cancer; MM, multiple myeloma; NA, not applicable; OMPC, oligometastatic prostate cancer; PPGL, pheochromocytoma and paraganglioma; RLT, radioligand therapy; SSTR2, somatostatin receptor 2; WM, Waldenström macroglobulinemia.

**Table S3: Compilation of annual therapeutic procedures in nuclear medicine in 2020 in the EU and the UK taken from the country fact sheets in Appendix C of Ligtveot et al. (2021)**

| | Therapeutic nuclear medicine procedures per year in the EU member states and the UK in 2020 | | | | | | | | | | | | | | | | | |
|---|---|---|---|---|---|---|---|---|---|---|---|---|---|---|---|---|---|---|
| | radioiodine (*including MIBG) | | SIRT microspheres radioembolisation | | antibody radiotherapies | | radioligand therapies | | | bone pain palliation | | | | | radiosynoviorthesis with colloids | | | |
| | $^{131}I$ | | | | | | | | | | | | | | | | | |
| country | benign | cancer* | $^{166}Ho$ | $^{90}Y$ | $^{90}Y$ | $^{177}Lu$ | $^{177}Lu$-DOTATATE | $^{177}Lu$-PSMA | $^{225}Ac$-PSMA | $^{223}RaCl_2$ | $^{153}Sm$-EDTMP | $^{89}SrCl_2$ | $^{32}P$ | $^{188}Re$-HEDP | $^{90}Y$ | $^{169}Er$ | $^{186}Re$ | |
| Austria | 1366 | 2564 | 0 | 2 | 2 | 7 | 756 | 1232 | 0 | 237 | 21 | 0 | 0 | 0 | 360 | 1050 | 36 | |
| Belgium | | 2729 | | 251 | 11 | 0 | 100 | 50 | 0 | 1498 | 0 | 0 | 0 | 0 | 28 | 0 | 2 | |
| Bulgaria | 2000 | 0 | 0 | 0 | 0 | 0 | 0 | 0 | 0 | 30 | 0 | 0 | 0 | 0 | 0 | 0 | 0 | |
| Croatia | 4176 | 4950 | 0 | 0 | 6 | 6 | 0 | 0 | 0 | 72 | 0 | 0 | 0 | 0 | 0 | 0 | 0 | |
| Cyprus | 0 | 220 | 0 | 0 | 0 | 0 | 0 | 0 | 0 | 27 | 0 | 0 | 0 | 0 | 0 | 0 | 0 | |
| Czech Rep. | 199 | 1292 | 0 | 0 | 2 | 0 | 160 | 0 | 0 | 335 | 34 | 96 | 0 | 0 | 0 | 25 | 39 | |
| Denmark | | | | | | | | | | | | | | | | | | |
| Estonia | 660 | 476 | 0 | 0 | 0 | 0 | 3 | 45 | 0 | 108 | 6 | 6 | 3 | 0 | 11 | 0 | 2 | |
| Finland | 155 | 117 | 0 | 0 | 0 | 0 | 0 | 30 | 0 | 20 | 0 | 0 | 29 | 0 | 0 | 0 | 0 | |
| France | 3925 | 6171 | 0 | 426 | 61 | 0 | 1100 | 690 | 0 | 104 | 130 | 0 | 0 | 0 | 276 | 161 | 161 | |
| Germany | 9435 | 5610 | 0 | 1480 | 64 | 13 | 4718 | 3570 | 1658 | 230 | 163 | 0 | 0 | 25 | 995 | 1875 | 1063 | |
| Greece | 1050 | 6921 | 0 | 480 | 0 | 18 | 36 | 15 | 0 | 330 | 24 | 6 | 0 | 33 | 975 | 30 | 30 | |
| Hungary | 1366 | 702 | 75 | 4 | 2 | 0 | 0 | 0 | 0 | 237 | 36 | 0 | 0 | 0 | 500 | 0 | 100 | |
| Ireland | 336 | 244 | 0 | 75 | 0 | 2 | 0 | 0 | 0 | 310 | 2 | 0 | 2 | 0 | 55 | 2 | 2 | |
| Italy | 1646 | 4982 | 6 | 512 | 9 | 21 | 3525 | 647 | 0 | 1361 | 17 | 0 | 0 | 0 | 11 | 0 | 0 | |
| Latvia | 250 | 253 | 0 | 0 | 0 | 0 | 0 | 0 | 0 | 1 | 0 | 0 | 0 | 0 | 0 | 0 | 0 | |
| Lithuania | 500 | 1 | 0 | 0 | 0 | 0 | 0 | 0 | 0 | 38 | 0 | 0 | 0 | 0 | 0 | 0 | 0 | |
| Luxemburg | 110 | 220 | 0 | 11 | 0 | 0 | 0 | 0 | 0 | 31 | 12 | 0 | 0 | 0 | 0 | 1 | 2 | |
| Malta | 45 | 52 | 0 | 3 | 0 | 0 | 0 | 0 | 0 | 0 | 0 | 0 | 0 | 0 | 0 | 0 | 0 | |
| Netherlands | | 1394 | 0 | 25 | 0 | 0 | 900 | 0 | 0 | 1021 | 9 | 44 | 15 | 0 | 0 | 0 | 0 | |
| Poland | 19445 | 2697 | 0 | 53 | 0 | 0 | 350 | 0 | 0 | 714 | 112 | 60 | 0 | 0 | 664 | 101 | 210 | |
| Portugal | 885 | 957 | 9 | 43 | 0 | 0 | 40 | 0 | 0 | 98 | 0 | 0 | 0 | 0 | 1 | 0 | 0 | |
| Romania | 0 | 2600 | 0 | 0 | 0 | 0 | 0 | 0 | 0 | 0 | 0 | 0 | 0 | 0 | 0 | 0 | 0 | |
| Slovakia | 1133 | 5326 | 0 | 0 | 0 | 0 | 0 | 135 | 0 | 1058 | 8 | 8 | 0 | 0 | 0 | 0 | 0 | |
| Slovenia | | 700 | 0 | 2 | 0 | 0 | 17 | 0 | 0 | 31 | 0 | 0 | 0 | 0 | 0 | 0 | 3 | |
| Spain | 5484 | 8232 | 168 | 912 | 84 | 0 | 336 | 0 | 0 | 240 | 72 | 0 | 12 | 24 | 600 | 84 | 132 | |
| Sweden | 1502 | 483 | 0 | 4 | 0 | 0 | 444 | 0 | 0 | 1466 | 6 | 0 | 50 | 0 | 1 | 0 | 0 | |
| **EU-27** | **55668** | **59893** | **258** | **4283** | **241** | **67** | **12485** | **6414** | **1658** | **9597** | **652** | **220** | **111** | **82** | **4477** | **3329** | **1782** | **161217** |
| **EU-4** | **20490** | **24995** | **174** | **3330** | **218** | **34** | **9679** | **4907** | **1658** | **1935** | **382** | **0** | **12** | **49** | **1882** | **2120** | **1356** | **73221** |
| **UK** | **5623** | **5623** | **18** | **1499** | **9** | **64** | **3438** | **1189** | **0** | **7077** | **0** | **0** | **73** | **0** | **329** | **0** | **82** | **25024** |

Ligtvoet, A., Scholten, C., Davé, A., King, R., Petrosova, L. and Chiti, A., Study on sustainable and resilient supply of medical radioisotopes in the EU, Goulart De Medeiros, M. and Joerger, A. editor(s), EUR 30690 EN, Publications Office of the European Union, Luxembourg, 2021, ISBN 978-92-76-37422-0, doi:10.2760/642561, JRC124565

**Figure S1: Data flow for estimating the number of patients biologically and clinically eligible for radioligand therapies based on prevalence and incidence data.**

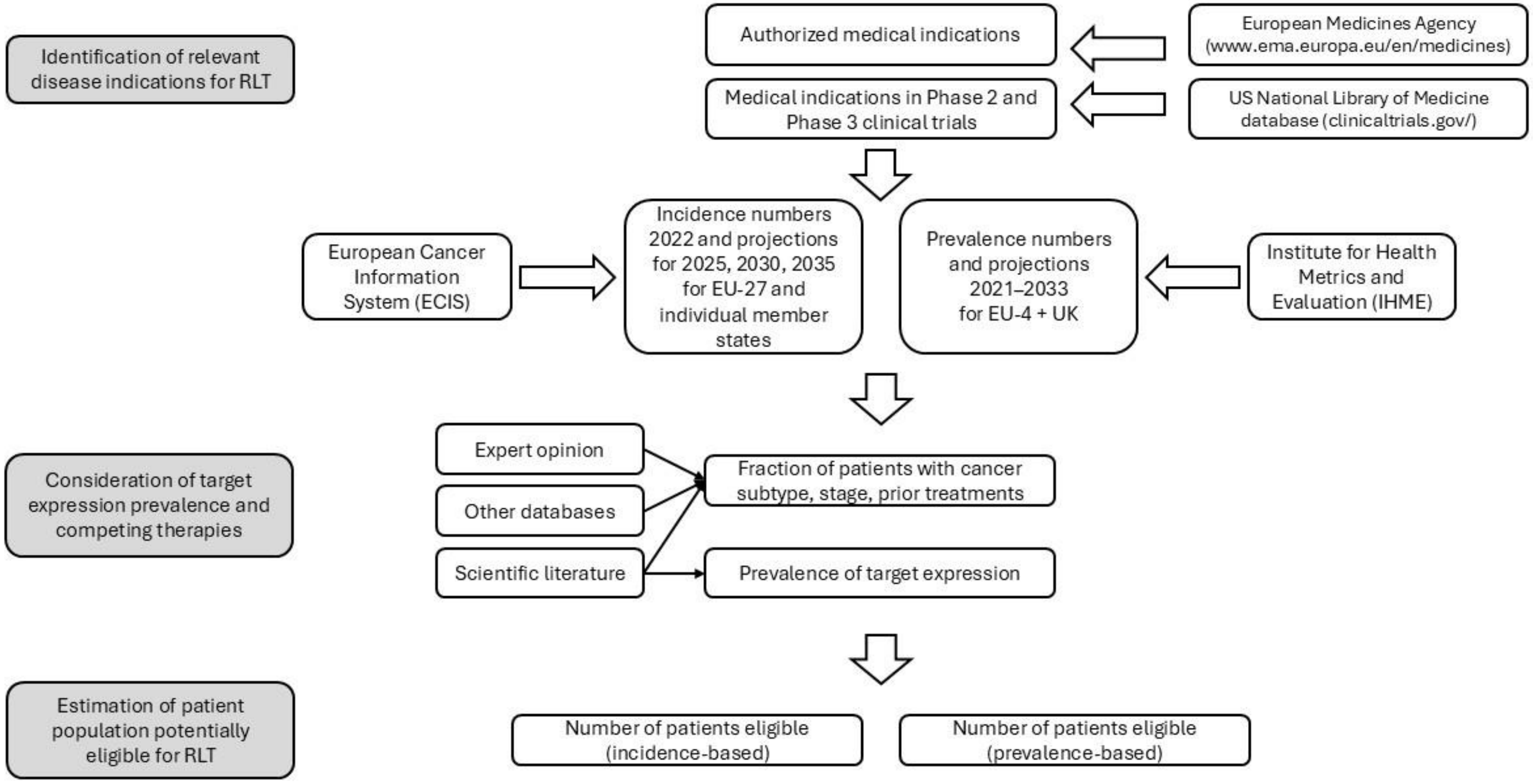

# Description of Data Sources – ECIS and IHME

### European Cancer Information System (ECIS)

The European Cancer Information System (ECIS) has been developed by the European Commission's Joint Research Centre (JRC) to collect, analyse, and disseminate data on cancer incidence, mortality, and prevalence in the European Union (EU). ECIS gathers cancer data mainly through collaborations with national cancer registries and collaborates with these to ensure data quality, completeness, and comparability across countries.[1,2] This involves standardizing data collection methods, data formats, and definitions according to international guidelines, such as those provided by the International Agency for Research on Cancer (IARC) and the European Network of Cancer Registries (ENCR). The ECIS team continuously monitors the quality of the data and performs data validation and quality checks to ensure data accuracy and reliability.[2] ECIS aims to provide timely, reliable, and comparable data on cancer to facilitate a better understanding of the disease's patterns, trends, and risk factors in support of the European Commission's efforts in cancer prevention, control, and research, as well as to inform policymaking and public health strategies in the EU. This, in turn, helps to identify areas that require targeted interventions and to monitor the progress towards the objectives of the European Union's Beating Cancer Plan, i.e., to reduce the burden of cancer on society.[1,2]

For the present purpose, the ECIS has a limitation because it categorises cancers according to the location of the primary cancers. Thus, for example, for neuroendocrine tumours which can occur in different organs, ECIS cannot provide incidence numbers and other information sources are required. Moreover, ECIS provides incidence numbers only for the main categories leukaemia, Non-Hodgkin's lymphoma and multiple myeloma grouped under the hierarchically highest ICD-10 codes. However, for the present work there is the need to quantify incidences of various specific sub-types of these cancers. A partitioning into the relevant haematological cancer subtypes was performed with the help of incidence data form the UK's Haematological Malignancies Research Network as presented in this Supplementary Material (pp. 39-42).

### The Institute for Health Metrics and Evaluation (IHME)

The Institute for Health Metrics and Evaluation (IHME) is an independent global health research center at the University of Washington. The Institute for Health Metrics and Evaluation (IHME), leads the Global Burden of Disease (GBD) study, advertised by The Lancet as the most comprehensive worldwide observational epidemiological study to date. – Data are hosted by the Lancet Global Burden of Disease (GBD) Resource Centre

### Comment on the source of prevalence data

The International Agency for Research on Cancer (IARC) is an organisation of the World Health Organisation (WHO) and runs the Global Cancer Observatory (GCO) (https://gco.iarc.who.int) hosting the GLOBOCAN database.The 2022 GLOBOCAN data were computed from incidence data using sex-, site-, and age-specific ratios of incidence to 1-, 3-, and 5-year prevalence from Nordic countries for the period 2006–2015. The incidence data for 2022 were provided by ECIS in a JRC–IARC collaboration. Except for Cyprus where GLOBOCAN as an UN data base counts also the cancer incidences in the Turkish speaking part of the island (not part of the EU), incidence data are identical for EU-26.

For the present study prevalence data from IHME have been used and incidence data from ECIS, as far as they were available. Since ECIS and GLOBOCAN share the same incidence data sets as input for GLOBOCAN's prevalences, we consider the use of IHME an asset for the present study. In this way two different institutions are providing the basic input data and a hypothetical quality issue with ECIS data (and, hence, the modelling of GLOBOCAN's prevalences) or vice versa in the IHME data would not affect both approaches simultaneously. Nevertheless, both data sources have a high reputation and are committed to highest quality standards.

### References


1 Giusti F, Martos C, Trama A, Bettio M, Sanvisens A, Audisio R, Arndt V, Francisci S, Dochez C, Ribes J, Fernández LP, Gavin A, Gatta G, Marcos-Gragera R, Lievens Y, Allemani C, De Angelis R, Visser O, Van Eycken L; ENCR Working Group on Treatment Data Harmonisation. Cancer treatment data available in European cancer registries: Where are we and where are we going? *Front Oncol.* 2023;**13**:1109978. doi: 10.3389/fonc.2023.1109978

2 Giusti F, Martos C, Adriani S, Flego M, Carvalho RN, Bettio M, Ben E. The Joint Research Centre-European Network of Cancer Registries Quality Check Software (JRC-ENCR QCS). *Front Oncol.* 2023; **13**:1250195. doi: 10.3389/fonc.2023.1250195

3 Bray F, Laversanne M, Sung H, Ferlay J, Siegel RL, Soerjomataram I, Jemal A. Global cancer statistics 2022: GLOBOCAN estimates of incidence and mortality worldwide for 36 cancers in 185 countries. *CA Cancer J Clin.* 2024; **74**(3):229-263. doi: 10.3322/caac.21834

**Table S4: Input parameters for the calculation of the patient pool eligible for RLT based on cancer prevalence in the EU-4 + UK and molecular target expression levels**

| Indication (sub-indication), by cancer type | Parameter | Value | Source |
|---|---|---|---|
| ***Neuroendocrine tumors*** | | | |
| NET (metastatic, 2L) | Prevalence NET | 0·00054 | Chauhan et al. (2020)[1] |
| | Share metastatic | 0·43 | McDonnell et al. (2022)[2] |
| | Share progression to ≥ 2L | 0·63 | Rinke et al. (2009)[2] |
| | Prevalence target marker SSTR2 | 0·87 | Childs et al. (2016)[4] |
| GEP-NET (G1–G2, metastatic, 2L) | Prevalence GEP-NET | 0·000376 | Chauhan et al. (2020)[1] |
| | Share G1 and G2 | 0·85 | Mitjavila et al. (2023)[5] |
| | Share metastatic | 0·43 | McDonnell et al. (2022)[2] |
| | Share progression to ≥ 2L | 0·63 | Rinke et al. (2009)[3] |
| | Prevalence target marker SSTR2 | 0·87 | Childs et al. (2016)[4] |
| GEP-NET (G2–G3, metastatic) | Prevalence GEP-NET | 0·000376 | Chauhan et al. (2020)[1] |
| | Share G2 and G3 | 0·71 | Mitjavila et al. (2023)[5] |
| | Share metastatic | 0·43 | McDonnell et al. (2022)[25] |
| | Prevalence target marker SSTR2 | 0·87 | Childs et al. (2016)[4] |
| ***Prostate cancer*** | | | |
| mCRPC (2L–3L) | Prevalence prostate cancer (2023–2033)[a] | 0·00656 (FR)<br>0·00848 (GER)<br>0·00726 (IT)<br>0·00608 (ESP)<br>0·00656 (UK) | IHME[6] |
| | Share of mCRPC in prostate cancer | 0·0185 | Shore et al. (2020)[7] |
| | Share progression to ≥2L | 0·3702 | Leith et al. (2022)[8] |
| | Prevalence target marker PSMA | 0·87 | Calais & Czernin (2021)[9] |
| mHSPC | Prevalence prostate cancer (2029–2033)[a] | 0·00670 (FR)<br>0·00885 (GER)<br>0·00785 (IT)<br>0·00675 (ESP)<br>0·00683 (UK) | IHME[6] |
| | Share of mHSPC in prostate cancer | 0·0176 | Spandonaro et al. (2021)[10] |
| | Prevalence target marker PSMA | 0·87 | Calais & Czernin (2021)[9] |
| OMPC | Prevalence prostate cancer (2029–2033)[a] | 0·00670 (FR)<br>0·00885 (GER)<br>0·00785 (IT)<br>0·00675 (ESP)<br>0·00683 (UK) | IHME[6] |
| | Share of mHSPC in prostate cancer | 0·0176 | Spandonaro et al. (2021)[10] |
| | Share of OMPC in mHSPC | 0·2 | Gong et al. (2024)[11] |
| | Prevalence target marker PSMA | 0·87 | Calais & Czernin (2021)[9] |
| ***Haematological malignancies*** | | | |
| AML | Prevalence AML (2025–2033)[a] | 0·00005 (FR)<br>0·00041 (GER)<br>0·00007 (IT)<br>0·00005 (ESP)<br>0·00005 (UK) | IHME[6] |
| | Prevalence target marker CD45 | 0·972 | Khalidi et al. (1998)[12] |
| LPL/WM | Prevalence LPL/WM | 0·0000103 | Orphanet[13] |
| MM | Prevalence MM (2029–2033)[a] | 0·00028 (FR)<br>0·00037 (GER)<br>0·00050 (IT)<br>0·00033 (ESP)<br>0·00043 (UK) | |
| | share progression to ≥6L | 0·005 | Kanas et al. (2021)[14] |
| ***Kidney cancer*** | | | |
| ccRCC (metastatic, 2L) | Prevalence kidney cancer (2031–2033)[a] | 0·00088 (FR) | IHME[6] |

| | | | |
|---|---|---|---|
| | | 0·00121 (GER)<br>0·00121 (IT)<br>0·00124 (ESP)<br>0·00089 (UK) | |
| | Share of RCC in kidney cancer | 0·925 | Sachdeva & Bagi (2023)[15] |
| | Share of ccRCC in RCC | 0·8 | Tostain et al. (2010)[16] |
| | Share metastatic | 0·106 | Monda et al. (2023)[17] |
| | Share progression to ≥2L | 0·513 | Parosanu et al. (2023)[18] |
| | Myelotoxicity limitation | 0.15 | Muselaers et al. (2016)[19] |
| | Prevalence target marker CA9 | 0·912 | Soyupak et al. (2005)[20] |
| ***Brain and CNS tumors*** | | | |
| Neuroblastoma / CNS (children) | Share of population below 15 years | 0·15 | World Bank (2024)[21] |
| | Prevalence neuroblastoma and CNS (children) | 0·0003 | Orphanet[13] |
| | Prevalence target marker B7-H3 | 0·8 | Kontos et al. (2021)[22] |

[a]Average of the reported years.
2L: second line of treatment; 3L, third line of treatment; AML: acute myeloid leukemia; ccRCC: clear cell renal cell carcinoma; CNS: central nervous system; DLBCL-CNS: diffuse large B-cell lymphoma of the central nervous system; G1: Grade 1 tumor; GEP-NET: gastroenteropancreatic neuroendocrine tumor; LPL, lymphoplasmacytic lymphoma; mCRPC: metastatic castration resistant prostate cancer; mHSPC: metastatic hormone sensitive prostate cancer; MM: multiple myeloma; NET: neuroendocrine tumor; OMPC: oligometastatic prostate cancer; PSMA: prostate-specific membrane antigen; RCC: renal cell carcinoma; RLT, radioligand therapy; SSTR: somatostatin receptor; WM, Waldenström macroglobulinemia.
FR: France; GER: Germany; IT: Italy; ESP: Spain; UK: United Kingdom

## References


1. Chauhan A, Kohn E, Del Rivero J. Neuroendocrine Tumors-Less Well Known, Often Misunderstood, and Rapidly Growing in Incidence. *JAMA Oncol* 2020; 6(1): 21-2.
2. McDonnell M, Bouvier C, Pavel ME, et al. Survey of challenges in access to diagnostics and treatment for neuroendocrine tumor patients (SCAN): The diagnostic process of GEP-NETs in Australia, Canada, China, France, Germany, the United Kingdom, and the United States of America. *Journal of Clinical Oncology* 2022; **40**(4_suppl): 502.
3. Rinke A, Müller HH, Schade-Brittinger C, et al. Placebo-controlled, double-blind, prospective, randomized study on the effect of octreotide LAR in the control of tumor growth in patients with metastatic neuroendocrine midgut tumors: a report from the PROMID Study Group. *J Clin Oncol* 2009; **27**(28): 4656-63.
4. Childs A, Vesely C, Ensell L, et al. Expression of somatostatin receptors 2 and 5 in circulating tumour cells from patients with neuroendocrine tumours. *Br J Cancer* 2016; **115**(12): 1540-7.
5. Mitjavila M, Jimenez-Fonseca P, Bello P, et al. Efficacy of [(177)Lu]Lu-DOTATATE in metastatic neuroendocrine neoplasms of different locations: data from the SEPTRALU study. *Eur J Nucl Med Mol Imaging* 2023; **50**(8): 2486-500.
6. Institute for Health Metrics and Evaluation. IHME. 2026. https://www.healthdata.org/ (accessed 31 January 2026).
7. Shore ND, Oliver L, Shui I, et al. Review of the real-world prevalence of mHSPC, nmCRPC, mCRPC, and gene alterations associated with HRR in prostate cancer (PC). *Journal of Clinical Oncology* 2020; **38**(6).
8. Leith A, Kim J, Ribbands A, Clayton E, Yang L, Ghate SR. Real-World Treatment Patterns in Metastatic Castration-Resistant Prostate Cancer Across Europe (France, Germany, Italy, Spain, and the United Kingdom) and Japan. *Adv Ther* 2022; **39**(5): 2236-55.
9. Calais J, Czernin J. PSMA Expression Assessed by PET Imaging Is a Required Biomarker for Selecting Patients for Any PSMA-Targeted Therapy. *J Nucl Med* 2021; **62**(11): 1489-91.
10. Spandonaro F, D'Angela D, Polistena B, et al. Prevalence of Prostate Cancer at Different Clinical Stages in Italy: Estimated Burden of Disease Based on a Modelling Study. *Biology* (Basel) 2021; **10**(3).
11. Gong J, Janes JL, Trustram Eve C, et al. Epidemiology, treatment patterns, and clinical outcomes in de novo oligometastatic hormone-sensitive prostate cancer. *Cancer* 2024; **130**(22): 3815-25.
12. Khalidi HS, Medeiros LJ, Chang KL, Brynes RK, Slovak ML, Arber DA. The immunophenotype of adult acute myeloid leukemia: high frequency of lymphoid antigen expression and comparison of immunophenotype, French-American-British classification, and karyotypic abnormalities. *Am J Clin Pathol* 1998; **109**(2): 211-20.
13. Orphanet. Rare diseases. https://www.orpha.net/ (accessed 31 January 2026).
14. Kanas G, Clark O, Keeven K, Nersesyan K, Sansbury L, Hogea C. Estimate of multiple myeloma patients by line of therapy in the USA: population-level projections 2020-2025. *Future Oncol* 2021; **17**(8): 921-30.
15. Sachdeva KJBRPC, B. Renal Cell Carcinoma. 2023. https://emedicine.medscape.com/article/281340-overview.
16. Tostain J, Li G, Gentil-Perret A, Gigante M. Carbonic anhydrase 9 in clear cell renal cell carcinoma: a marker for diagnosis, prognosis and treatment. *Eur J Cancer* 2010; **46**(18): 3141-8.

17. Monda SM, Lui HT, Pratsinis MA, Chandrasekar T, Evans CP, Dall'Era MA. The Metastatic Risk of Renal Cell Carcinoma by Primary Tumor Size and Subtype. *Eur Urol Open Sci* 2023; **52**: 137-44.

18. Parosanu AI, Baston C, Stanciu IM, Parlog CF, Nitipir C. Second-Line Treatment of Metastatic Renal Cell Carcinoma in the Era of Predictive Biomarkers. *Diagnostics* (Basel) 2023; **13**(14).

19. Muselaers CH, Boers-Sonderen MJ, van Oostenbrugge TJ, et al. Phase 2 Study of Lutetium 177-Labeled Anti-Carbonic Anhydrase IX Monoclonal Antibody Girentuximab in Patients with Advanced Renal Cell Carcinoma. *Eur Urol* 2016; **69**(5): 767-70.

20. Soyupak B, Erdoğan S, Ergin M, Seydaoğlu G, Kuzgunbay B, Tansuğ Z. CA9 expression as a prognostic factor in renal clear cell carcinoma. *Urol Int* 2005; **74**(1): 68-73.

21. World Bank Group. https://data.worldbank.org/indicator/SP.POP.TOTL (accessed 31 January 2026).

22. Kontos F, Michelakos T, Kurokawa T, et al. B7-H3: An Attractive Target for Antibody-based Immunotherapy. *Clin Cancer Res* 2021; **27**(5): 1227-35.

**Table S5: Evolution of patient pools eligible for radioligand therapies in France by medical indication and sub-indication from 2021 to 2033**

| | 2021 | 2022 | 2023 | 2024 | 2025 | 2026 | 2027 | 2028 | 2029 | 2030 | 2031 | 2032 | 2033 |
|---|---|---|---|---|---|---|---|---|---|---|---|---|---|
| **Hematological Malignancies** | **0** | **0** | **0** | **0** | **0** | **0** | **0** | **0** | **0** | **4,431** | **4,512** | **4,594** | **4,674** |
| AML | 0 | 0 | 0 | 0 | 0 | 0 | 0 | 0 | 0 | 3,656 | 3,733 | 3,813 | 3,890 |
| LPL / WM | 0 | 0 | 0 | 0 | 0 | 0 | 0 | 0 | 0 | 681 | 682 | 683 | 684 |
| MM (6L) | 0 | 0 | 0 | 0 | 0 | 0 | 0 | 0 | 0 | 95 | 97 | 98 | 100 |
| **Kindey Cancer** | **0** | **0** | **0** | **0** | **0** | **0** | **0** | **0** | **0** | **0** | **638** | **649** | **660** |
| ccRCC (metastatic, 2L) | 0 | 0 | 0 | 0 | 0 | 0 | 0 | 0 | 0 | 0 | 638 | 649 | 660 |
| **NET** | **5,102** | **5,126** | **5,148** | **5,164** | **5,178** | **6,882** | **6,896** | **6,909** | **8,773** | **8,790** | **8,807** | **8,823** | **8,839** |
| GEP-NET | 5,102 | 5,126 | 5,148 | 5,164 | 5,178 | 6,882 | 6,896 | 6,909 | 6,923 | 6,936 | 6,949 | 6,962 | 6,975 |
| NETTER-1 (G1-G2, metastatic, 2L) | 5,102 | 5,126 | 5,148 | 5,164 | 5,178 | 5,190 | 5,201 | 5,211 | 5,221 | 5,231 | 5,241 | 5,251 | 5,261 |
| NETTER-2 (G2-G3, metastatic) / Non-NETTER-1 | 0 | 0 | 0 | 0 | 0 | 1,691 | 1,695 | 1,698 | 1,701 | 1,705 | 1,708 | 1,711 | 1,714 |
| NET (metastatic, 2L) / Non-GEP-NET | 0 | 0 | 0 | 0 | 0 | 0 | 0 | 0 | 1,850 | 1,854 | 1,857 | 1,861 | 1,864 |
| **Prostate Cancer** | **0** | **319** | **327** | **336** | **345** | **354** | **2,592** | **2,658** | **9,735** | **9,970** | **10,207** | **10,432** | **10,661** |
| mCRPC (2L-3L) | 0 | 319 | 327 | 336 | 345 | 354 | 2,592 | 2,658 | 2,724 | 2,790 | 2,856 | 2,919 | 2,983 |
| OMPC | 0 | 0 | 0 | 0 | 0 | 0 | 0 | 0 | 0 | 1,436 | 1,470 | 1,503 | 1,536 |
| mHSPC / non-OMPC | | | | | | | | | 7,011 | 5,744 | 5,881 | 6,010 | 6,143 |
| **Other** | **0** | **0** | **0** | **0** | **0** | **0** | **2,486** | **2,491** | **2,495** | **2,500** | **2,505** | **2,510** | **2,514** |
| Neuroblastoma / CNS (children) | 0 | 0 | 0 | 0 | 0 | 0 | 2,486 | 2,491 | 2,495 | 2,500 | 2,505 | 2,510 | 2,514 |
| **Total** | **5,102** | **5,445** | **5,475** | **5,500** | **5,523** | **7,235** | **11,973** | **12,058** | **21,004** | **25,691** | **26,669** | **27,008** | **27,349** |

2L: second line of treatment; AML: acute myeloid leukemia; ccRCC: clear cell renal cell carcinoma; CNS: central nervous system; G1: Grade 1; GEP-NET: gastroenteropancreatic neuroendocrine tumor; mCRPC: metastatic castration resistant prostate cancer; mHSPC: metastatic hormone sensitive prostate cancer; MM: multiple myeloma; NET: neuroendocrine tumor; OMPC: oligometastatic prostate cancer

**Table S6: Evolution of patient pools eligible for radioligand therapies in Germany by medical indication and sub-indication from 2021 to 2033**

| | 2021 | 2022 | 2023 | 2024 | 2025 | 2026 | 2027 | 2028 | 2029 | 2030 | 2031 | 2032 | 2033 |
|---|---|---|---|---|---|---|---|---|---|---|---|---|---|
| **Hematological Malignancies** | **0** | **0** | **0** | **0** | **0** | **0** | **0** | **0** | **0** | **35,278** | **35,626** | **36,001** | **36,371** |
| AML | 0 | 0 | 0 | 0 | 0 | 0 | 0 | 0 | 0 | 34,284 | 34,630 | 35,003 | 35,372 |
| LPL / WM | 0 | 0 | 0 | 0 | 0 | 0 | 0 | 0 | 0 | 836 | 835 | 835 | 835 |
| MM (6L) | 0 | 0 | 0 | 0 | 0 | 0 | 0 | 0 | 0 | 158 | 160 | 162 | 164 |
| **Kindey Cancer** | **0** | **0** | **0** | **0** | **0** | **0** | **0** | **0** | **0** | **0** | **1,118** | **1,129** | **1,140** |
| ccRCC (metastatic, 2L) | 0 | 0 | 0 | 0 | 0 | 0 | 0 | 0 | 0 | 0 | 1,118 | 1,129 | 1,140 |
| **NET** | **6,267** | **6,319** | **6,387** | **6,412** | **6,422** | **8,522** | **8,523** | **8,520** | **10,795** | **10,792** | **10,790** | **10,787** | **10,784** |
| GEP-NET | 6,267 | 6,319 | 6,387 | 6,412 | 6,422 | 8,522 | 8,523 | 8,520 | 8,518 | 8,516 | 8,514 | 8,512 | 8,510 |
| NETTER-1 (G1-G2, metastatic, 2L) | 6,267 | 6,319 | 6,387 | 6,412 | 6,422 | 6,427 | 6,428 | 6,426 | 6,425 | 6,423 | 6,422 | 6,420 | 6,418 |
| NETTER-2 (G2-G3, metastatic) / Non-NETTER-1 | 0 | 0 | 0 | 0 | 0 | 2,094 | 2,095 | 2,094 | 2,094 | 2,093 | 2,093 | 2,092 | 2,092 |
| NET (metastatic, 2L) / Non-GEP-NET | 0 | 0 | 0 | 0 | 0 | 0 | 0 | 0 | 2,277 | 2,276 | 2,276 | 2,275 | 2,274 |
| **Prostate Cancer** | **0** | **549** | **561** | **572** | **582** | **593** | **4,310** | **4,382** | **15,903** | **16,137** | **16,373** | **16,590** | **16,787** |
| mCRPC (2L-3L) | 0 | 549 | 561 | 572 | 582 | 593 | 4,310 | 4,382 | 4,450 | 4,515 | 4,581 | 4,642 | 4,697 |
| OMPC | 0 | 0 | 0 | 0 | 0 | 0 | 0 | 0 | 0 | 2,324 | 2,358 | 2,390 | 2,418 |
| mHSPC / non-OMPC | | | | | | | | | 11,453 | 9,297 | 9,433 | 9,558 | 9,672 |
| **Other** | **0** | **0** | **0** | **0** | **0** | **0** | **3,072** | **3,071** | **3,071** | **3,070** | **3,069** | **3,068** | **3,068** |
| Neuroblastoma / CNS (children) | 0 | 0 | 0 | 0 | 0 | 0 | 3,072 | 3,071 | 3,071 | 3,070 | 3,069 | 3,068 | 3,068 |
| **Total** | **6,267** | **6,868** | **6,948** | **6,984** | **7,004** | **9,115** | **15,905** | **15,974** | **29,769** | **65,277** | **66,975** | **67,576** | **68,151** |

2L: second line of treatment; AML: acute myeloid leukemia; ccRCC: clear cell renal cell carcinoma; CNS: central nervous system; G1: Grade 1; GEP-NET: gastroenteropancreatic neuroendocrine tumor; mCRPC: metastatic castration resistant prostate cancer; mHSPC: metastatic hormone sensitive prostate cancer; MM: multiple myeloma; NET: neuroendocrine tumor; OMPC: oligometastatic prostate cancer

**Table S7: Evolution of patient pools eligible for radioligand therapies in Italy by medical indication and sub-indication from 2021 to 2033**

| | 2021 | 2022 | 2023 | 2024 | 2025 | 2026 | 2027 | 2028 | 2029 | 2030 | 2031 | 2032 | 2033 |
|---|---|---|---|---|---|---|---|---|---|---|---|---|---|
| **Hematological Malignancies** | **0** | **0** | **0** | **0** | **0** | **0** | **0** | **0** | **0** | **4,554** | **4,617** | **4,683** | **4,748** |
| AML | 0 | 0 | 0 | 0 | 0 | 0 | 0 | 0 | 0 | 3,836 | 3,897 | 3,961 | 4,023 |
| LPL / WM | 0 | 0 | 0 | 0 | 0 | 0 | 0 | 0 | 0 | 576 | 576 | 575 | 575 |
| MM (6L) | 0 | 0 | 0 | 0 | 0 | 0 | 0 | 0 | 0 | 143 | 145 | 147 | 150 |
| **Kindey Cancer** | **0** | **0** | **0** | **0** | **0** | **0** | **0** | **0** | **0** | **0** | **951** | **964** | **976** |
| ccRCC (metastatic, 2L) | 0 | 0 | 0 | 0 | 0 | 0 | 0 | 0 | 0 | 0 | 951 | 964 | 976 |
| **NET** | **4,454** | **4,447** | **4,447** | **4,443** | **4,438** | **5,880** | **5,877** | **5,874** | **7,440** | **7,437** | **7,434** | **7,431** | **7,427** |
| GEP-NET | 4,454 | 4,447 | 4,447 | 4,443 | 4,438 | 5,880 | 5,877 | 5,874 | 5,871 | 5,868 | 5,866 | 5,864 | 5,861 |
| NETTER-1 (G1-G2, metastatic, 2L) | 4,454 | 4,447 | 4,447 | 4,443 | 4,438 | 4,435 | 4,432 | 4,430 | 4,428 | 4,426 | 4,424 | 4,422 | 4,421 |
| NETTER-2 (G2-G3, metastatic) / Non-NETTER-1 | 0 | 0 | 0 | 0 | 0 | 1,445 | 1,444 | 1,444 | 1,443 | 1,442 | 1,442 | 1,441 | 1,440 |
| NET (metastatic, 2L) / Non-GEP-NET | 0 | 0 | 0 | 0 | 0 | 0 | 0 | 0 | 1,569 | 1,568 | 1,568 | 1,567 | 1,566 |
| **Prostate Cancer** | **0** | **310** | **317** | **324** | **331** | **339** | **2,477** | **2,537** | **9,292** | **9,523** | **9,758** | **9,994** | **10,220** |
| mCRPC (2L-3L) | 0 | 310 | 317 | 324 | 331 | 339 | 2,477 | 2,537 | 2,600 | 2,665 | 2,730 | 2,796 | 2,860 |
| OMPC | 0 | 0 | 0 | 0 | 0 | 0 | 0 | 0 | 0 | 1,372 | 1,406 | 1,440 | 1,472 |
| mHSPC / non-OMPC | | | | | | | | | 6,692 | 5,487 | 5,622 | 5,758 | 5,888 |
| **Other** | **0** | **0** | **0** | **0** | **0** | **0** | **2,118** | **2,117** | **2,116** | **2,115** | **2,115** | **2,114** | **2,113** |
| Neuroblastoma / CNS (children) | 0 | 0 | 0 | 0 | 0 | 0 | 2,118 | 2,117 | 2,116 | 2,115 | 2,115 | 2,114 | 2,113 |
| **Total** | **4,454** | **4,758** | **4,764** | **4,767** | **4,770** | **6,219** | **10,472** | **10,528** | **18,848** | **23,629** | **24,875** | **25,186** | **25,485** |

2L: second line of treatment; AML: acute myeloid leukemia; ccRCC: clear cell renal cell carcinoma; CNS: central nervous system; G1: Grade 1; GEP-NET: gastroenteropancreatic neuroendocrine tumor; mCRPC: metastatic castration resistant prostate cancer; mHSPC: metastatic hormone sensitive prostate cancer; MM: multiple myeloma; NET: neuroendocrine tumor; OMPC: oligometastatic prostate cancer

**Table S8: Evolution of patient pools eligible for radioligand therapies in Spain by medical indication and sub-indication from 2021 to 2033**

| | 2021 | 2022 | 2023 | 2024 | 2025 | 2026 | 2027 | 2028 | 2029 | 2030 | 2031 | 2032 | 2033 |
|---|---|---|---|---|---|---|---|---|---|---|---|---|---|
| **Hematological Malignancies** | **0** | **0** | **0** | **0** | **0** | **0** | **0** | **0** | **0** | **2,617** | **2,659** | **2,702** | **2,744** |
| AML | 0 | 0 | 0 | 0 | 0 | 0 | 0 | 0 | 0 | 2,059 | 2,097 | 2,136 | 2,176 |
| LPL / WM | 0 | 0 | 0 | 0 | 0 | 0 | 0 | 0 | 0 | 485 | 487 | 489 | 491 |
| MM (6L) | 0 | 0 | 0 | 0 | 0 | 0 | 0 | 0 | 0 | 73 | 75 | 76 | 78 |
| **Kindey Cancer** | **0** | **0** | **0** | **0** | **0** | **0** | **0** | **0** | **0** | **0** | **471** | **479** | **487** |
| ccRCC (metastatic, 2L) | 0 | 0 | 0 | 0 | 0 | 0 | 0 | 0 | 0 | 0 | 471 | 479 | 487 |
| **NET** | **3,565** | **3,566** | **3,589** | **3,612** | **3,634** | **4,846** | **4,873** | **4,899** | **6,238** | **6,266** | **6,291** | **6,314** | **6,335** |
| GEP-NET | 3,565 | 3,566 | 3,589 | 3,612 | 3,634 | 4,846 | 4,873 | 4,899 | 4,922 | 4,944 | 4,964 | 4,982 | 4,999 |
| NETTER-1 (G1-G2, metastatic, 2L) | 3,565 | 3,566 | 3,589 | 3,612 | 3,634 | 3,655 | 3,676 | 3,695 | 3,713 | 3,729 | 3,744 | 3,758 | 3,770 |
| NETTER-2 (G2-G3, metastatic) / Non-NETTER-1 | 0 | 0 | 0 | 0 | 0 | 1,191 | 1,198 | 1,204 | 1,210 | 1,215 | 1,220 | 1,225 | 1,229 |
| NET (metastatic, 2L) / Non-GEP-NET | 0 | 0 | 0 | 0 | 0 | 0 | 0 | 0 | 1,316 | 1,321 | 1,327 | 1,332 | 1,336 |
| **Prostate Cancer** | **0** | **188** | **194** | **200** | **207** | **214** | **1,581** | **1,639** | **6,069** | **6,296** | **6,532** | **6,772** | **7,010** |
| mCRPC (2L-3L) | 0 | 188 | 194 | 200 | 207 | 214 | 1,581 | 1,639 | 1,698 | 1,762 | 1,828 | 1,895 | 1,961 |
| OMPC | 0 | 0 | 0 | 0 | 0 | 0 | 0 | 0 | 0 | 907 | 941 | 975 | 1,010 |
| mHSPC / non-OMPC | | | | | | | | | 4,371 | 3,627 | 3,763 | 3,902 | 4,039 |
| **Other** | **0** | **0** | **0** | **0** | **0** | **0** | **1,757** | **1,766** | **1,774** | **1,782** | **1,789** | **1,796** | **1,802** |
| Neuroblastoma / CNS (children) | 0 | 0 | 0 | 0 | 0 | 0 | 1,757 | 1,766 | 1,774 | 1,782 | 1,789 | 1,796 | 1,802 |
| **Total** | **3,565** | **3,754** | **3,783** | **3,812** | **3,841** | **5,060** | **8,211** | **8,303** | **14,081** | **16,961** | **17,742** | **18,062** | **18,377** |

2L: second line of treatment; AML: acute myeloid leukemia; ccRCC: clear cell renal cell carcinoma; CNS: central nervous system; G1: Grade 1; GEP-NET: gastroenteropancreatic neuroendocrine tumor; mCRPC: metastatic castration resistant prostate cancer; mHSPC: metastatic hormone sensitive prostate cancer; MM: multiple myeloma; NET: neuroendocrine tumor; OMPC: oligometastatic prostate cancer

**Table S9: Evolution of patient pools eligible for radioligand therapies in the UK by medical indication and sub-indication from 2021 to 2033**

| | 2021 | 2022 | 2023 | 2024 | 2025 | 2026 | 2027 | 2028 | 2029 | 2030 | 2031 | 2032 | 2033 |
|---|---|---|---|---|---|---|---|---|---|---|---|---|---|
| **Hematological Malignancies** | **0** | **0** | **0** | **0** | **0** | **0** | **0** | **0** | **0** | **4,188** | **4,236** | **4,284** | **4,331** |
| AML | 0 | 0 | 0 | 0 | 0 | 0 | 0 | 0 | 0 | 3,360 | 3,404 | 3,449 | 3,492 |
| LPL / WM | 0 | 0 | 0 | 0 | 0 | 0 | 0 | 0 | 0 | 678 | 680 | 681 | 682 |
| MM (6L) | 0 | 0 | 0 | 0 | 0 | 0 | 0 | 0 | 0 | 149 | 152 | 155 | 157 |
| **Kindey Cancer** | **0** | **0** | **0** | **0** | **0** | **0** | **0** | **0** | **0** | **0** | **673** | **683** | **692** |
| ccRCC (metastatic, 2L) | 0 | 0 | 0 | 0 | 0 | 0 | 0 | 0 | 0 | 0 | 673 | 683 | 692 |
| **NET** | **5,049** | **5,092** | **5,110** | **5,128** | **5,145** | **6,842** | **6,861** | **6,879** | **8,739** | **8,759** | **8,778** | **8,795** | **8,812** |
| GEP-NET | 5,049 | 5,092 | 5,110 | 5,128 | 5,145 | 6,842 | 6,861 | 6,879 | 6,896 | 6,912 | 6,926 | 6,940 | 6,953 |
| NETTER-1 (G1-G2, metastatic, 2L) | 5,049 | 5,092 | 5,110 | 5,128 | 5,145 | 5,161 | 5,175 | 5,188 | 5,201 | 5,213 | 5,224 | 5,235 | 5,244 |
| NETTER-2 (G2-G3, metastatic) / Non-NETTER-1 | 0 | 0 | 0 | 0 | 0 | 1,682 | 1,686 | 1,691 | 1,695 | 1,699 | 1,702 | 1,706 | 1,709 |
| NET (metastatic, 2L) / Non-GEP-NET | 0 | 0 | 0 | 0 | 0 | 0 | 0 | 0 | 1,843 | 1,847 | 1,851 | 1,855 | 1,858 |
| **Prostate Cancer** | **0** | **346** | **351** | **358** | **364** | **371** | **2,694** | **2,744** | **9,995** | **10,184** | **10,367** | **10,555** | **10,744** |
| mCRPC (2L-3L) | 0 | 346 | 351 | 358 | 364 | 371 | 2,694 | 2,744 | 2,797 | 2,850 | 2,901 | 2,953 | 3,006 |
| OMPC | 0 | 0 | 0 | 0 | 0 | 0 | 0 | 0 | 0 | 1,467 | 1,493 | 1,520 | 1,548 |
| mHSPC / non-OMPC | | | | | | | | | 7,198 | 5,868 | 5,973 | 6,081 | 6,190 |
| **Other** | **0** | **0** | **0** | **0** | **0** | **0** | **2,473** | **2,480** | **2,486** | **2,491** | **2,497** | **2,502** | **2,506** |
| Neuroblastoma / CNS (children) | 0 | 0 | 0 | 0 | 0 | 0 | 2,473 | 2,480 | 2,486 | 2,491 | 2,497 | 2,502 | 2,506 |
| **Total** | **5,049** | **5,438** | **5,462** | **5,486** | **5,509** | **7,213** | **12,028** | **12,103** | **21,219** | **25,623** | **26,551** | **26,819** | **27,086** |

2L: second line of treatment; AML: acute myeloid leukemia; ccRCC: clear cell renal cell carcinoma; CNS: central nervous system; G1: Grade 1; GEP-NET: gastroenteropancreatic neuroendocrine tumor; mCRPC: metastatic castration resistant prostate cancer; mHSPC: metastatic hormone sensitive prostate cancer; MM: multiple myeloma; NET: neuroendocrine tumor; OMPC: oligometastatic prostate cancer

**Table S10: Evolution of patient pools eligible for radioligand therapies in the EU-4 countries (France, Germany, Italy, Spain) and the UK by medical indication and sub-indication from 2021 to 2033**

| | 2021 | 2022 | 2023 | 2024 | 2025 | 2026 | 2027 | 2028 | 2029 | 2030 | 2031 | 2032 | 2033 |
|---|---|---|---|---|---|---|---|---|---|---|---|---|---|
| **Hematological Malignancies** | **0** | **0** | **0** | **0** | **0** | **0** | **0** | **0** | **0** | **51,068** | **51,651** | **52,264** | **52,868** |
| AML | 0 | 0 | 0 | 0 | 0 | 0 | 0 | 0 | 0 | 47,194 | 47,762 | 48,361 | 48,952 |
| LPL / WM | 0 | 0 | 0 | 0 | 0 | 0 | 0 | 0 | 0 | 3,256 | 3,260 | 3,264 | 3,267 |
| MM (6L) | 0 | 0 | 0 | 0 | 0 | 0 | 0 | 0 | 0 | 619 | 629 | 639 | 649 |
| **Kindey Cancer** | **0** | **0** | **0** | **0** | **0** | **0** | **0** | **0** | **0** | **0** | **3,851** | **3,904** | **3,956** |
| ccRCC (metastatic, 2L) | 0 | 0 | 0 | 0 | 0 | 0 | 0 | 0 | 0 | 0 | 3,851 | 3,904 | 3,956 |
| **NET** | **24,437** | **24,550** | **24,681** | **24,759** | **24,817** | **32,972** | **33,030** | **33,081** | **41,985** | **42,044** | **42,099** | **42,150** | **42,197** |
| GEP-NET | 24,437 | 24,550 | 24,681 | 24,759 | 24,817 | 32,972 | 33,030 | 33,081 | 33,130 | 33,177 | 33,220 | 33,261 | 33,298 |
| NETTER-1 (G1-G2, metastatic, 2L) | 24,437 | 24,550 | 24,681 | 24,759 | 24,817 | 24,868 | 24,912 | 24,951 | 24,988 | 25,023 | 25,056 | 25,086 | 25,114 |
| NETTER-2 (G2-G3, metastatic) / Non-NETTER-1 | 0 | 0 | 0 | 0 | 0 | 8,104 | 8,118 | 8,130 | 8,143 | 8,154 | 8,165 | 8,175 | 8,184 |
| NET (metastatic, 2L) / Non-GEP-NET | 0 | 0 | 0 | 0 | 0 | 0 | 0 | 0 | 8,855 | 8,867 | 8,879 | 8,889 | 8,899 |
| **Prostate Cancer** | **0** | **1,712** | **1,750** | **1,789** | **1,829** | **1,871** | **13,654** | **13,960** | **50,993** | **52,109** | **53,237** | **54,343** | **55,423** |
| mCRPC (2L-3L) | 0 | 1,712 | 1,750 | 1,789 | 1,829 | 1,871 | 13,654 | 13,960 | 14,268 | 14,580 | 14,896 | 15,205 | 15,508 |
| OMPC | 0 | 0 | 0 | 0 | 0 | 0 | 0 | 0 | 0 | 7,506 | 7,668 | 7,827 | 7,983 |
| mHSPC / non-OMPC | 0 | 0 | 0 | 0 | 0 | 0 | 0 | 0 | 36,725 | 30,023 | 30,673 | 31,310 | 31,932 |
| **Other** | **0** | **0** | **0** | **0** | **0** | **0** | **11,906** | **11,925** | **11,942** | **11,959** | **11,975** | **11,989** | **12,003** |
| Neuroblastoma / CNS (children) | 0 | 0 | 0 | 0 | 0 | 0 | 11,906 | 11,925 | 11,942 | 11,959 | 11,975 | 11,989 | 12,003 |
| **Total** | **24,437** | **26,263** | **26,431** | **26,548** | **26,646** | **34,844** | **58,590** | **58,965** | **104,921** | **157,181** | **162,813** | **164,651** | **166,447** |

2L: second line of treatment; AML: acute myeloid leukemia; ccRCC: clear cell renal cell carcinoma; CNS: central nervous system; G1: Grade 1; GEP-NET: gastroenteropancreatic neuroendocrine tumor; mCRPC: metastatic castration resistant prostate cancer; mHSPC: metastatic hormone sensitive prostate cancer; MM: multiple myeloma; NET: neuroendocrine tumor; OMPC: oligometastatic prostate cancer

**Figure S2: Graphical presentation of the overall evolution of patient pools eligible for radioligand therapies from 2021 to 2033 based on the medical indications compiled in Tables S5 – S10 for Germany, France, Italy, Spain (EU-4), the UK and all together (EU-4 & UK).**

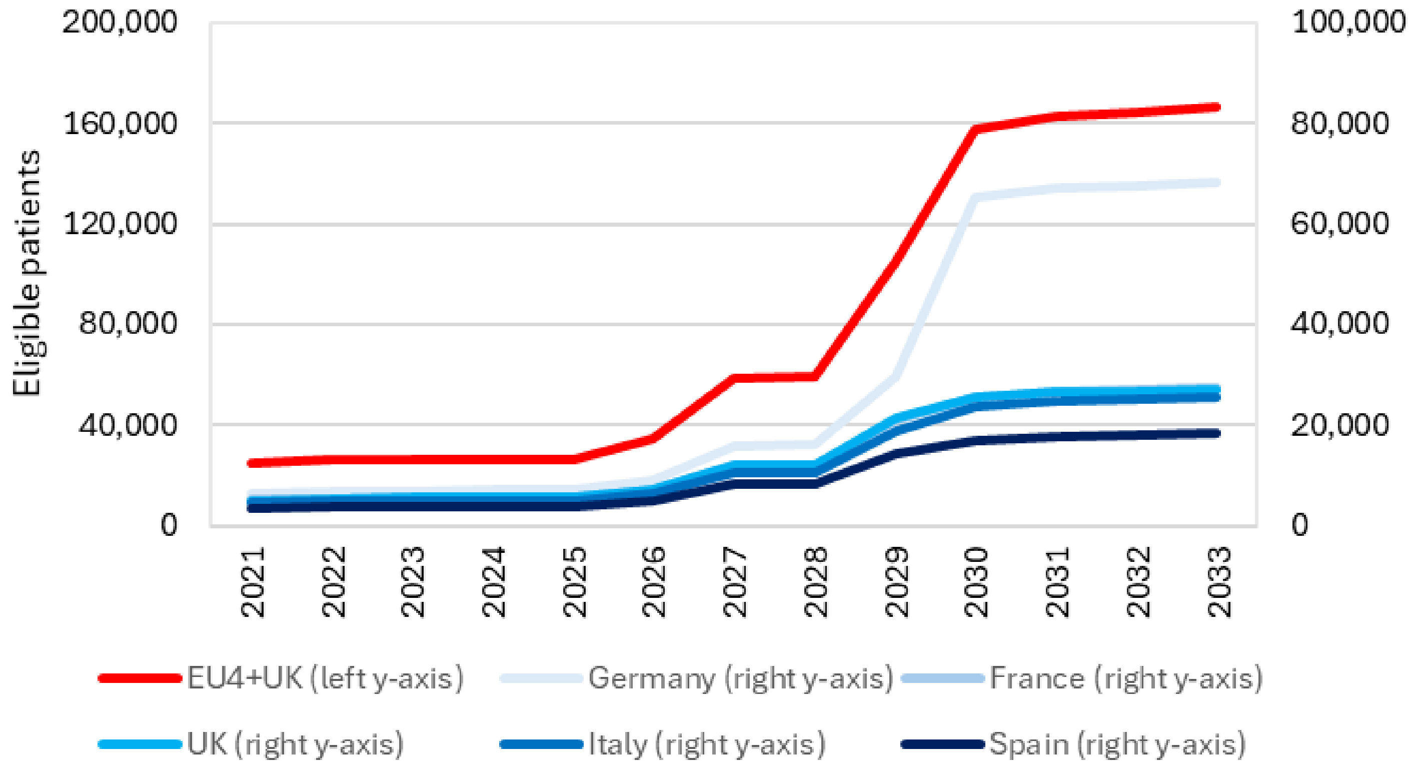

## Methodology – Estimating the number of eligible patients from incidence figures

In the present work the JRC derived the number of eligible patients per year for the EU-27 as a whole and for each individual member state from the incidence numbers provided by the European Cancer Information System (ECIS) that are available for the years 2022, and as projections for 2025, 2030 and 2035. The evaluations yield the fraction of the patients that develop during their course of disease the medical conditions that qualifies them to receive a given radioligand therapy based on existing or anticipated marketing authorisations. It is obvious that only a fraction of these will become eligible for therapy in the same year the incidence numbers are available. This fraction can even be zero in case that the marketing authorisation requires different lines of treatment before RLT. Nevertheless, this number is a valid approximation for the number of patients to be treated in the year for which the incidence numbers are available, because we can assume that the patients that become eligible for the treatment in the years to come are recovered from the patients that have been diagnosed with the cancer in the years before. In physics this would be described as a "steady state". The following tables use fictitious numbers to illustrate this concept.

For the illustration in Table S11a let us assume that we estimated from the incidence data in the reference year (year 0) that 200 patients will become eligible for the considered therapy. Let us assume that 15 of these are eligible immediately, while 20, 50, 70, 30 and 15 qualify for the therapy in 1, 2, 3, 4 and 5 years after diagnosis, respectively (see first row, year 0). The entries for the columns for the years 0 up to 5 in the row year 0 sum up in the last column to 200, which is the number of eligible patients we have obtained. In the same way, one year ago 15 patents were eligible, 20 become eligible now (in the year 0 of interest) and 50, 70, 30 and 15 in the successive years. Also, these entries in the row year -1 sum up to 200 (last column). In the present case the only assumptions that are required is that the progression of disease over time that defines the sequence 15 – 20 – 50 – 70 – 30 – 15 and the incidence numbers per year are (approximately) constant yielding 200 eligible patients also in the years before. In this way the sum in each row and each column yields always a number of 200 eligible patients. This reflects a steady state determined by stable incidence and fixed progression of disease.

**Table S11a: Illustration of a steady state with constant incidence numbers over time, resulting in 200 eligible patients per year, and a disease progressing the same way over 6 years.**

| | past | | | | | | | | | | reference year | future | | | | | number of eligible patients per year |
|---|---|---|---|---|---|---|---|---|---|---|---|---|---|---|---|---|---|
| **Year** | **-10** | **-9** | **-8** | **-7** | **-6** | **-5** | **-4** | **-3** | **-2** | **-1** | **0** | **1** | **2** | **3** | **4** | **5** | |
| **0** | | | | | | | | | | | 15 | 20 | 50 | 70 | 30 | 15 | 200 |
| **-1** | | | | | | | | | | 15 | 20 | 50 | 70 | 30 | 15 | | 200 |
| **-2** | | | | | | | | | 15 | 20 | 50 | 70 | 30 | 15 | | | 200 |
| **-3** | | | | | | | | 15 | 20 | 50 | 70 | 30 | 15 | | | | 200 |
| **-4** | | | | | | | 15 | 20 | 50 | 70 | 30 | 15 | | | | | 200 |
| **-5** | | | | | | 15 | 20 | 50 | 70 | 30 | 15 | | | | | | 200 |
| **-6** | | | | | 15 | 20 | 50 | 70 | 30 | 15 | | | | | | | 200 |
| **-7** | | | | 15 | 20 | 50 | 70 | 30 | 15 | | | | | | | | 200 |
| **-8** | | | 15 | 20 | 50 | 70 | 30 | 15 | | | | | | | | | 200 |
| **-9** | | 15 | 20 | 50 | 70 | 30 | 15 | | | | | | | | | | 200 |
| **-10** | 15 | 20 | 50 | 70 | 30 | 15 | | | | | | | | | | | 200 |
| | ... | ... | ... | ... | ... | 200 | 200 | 200 | 200 | 200 | 200 | | | | | | |
| | | | | | | steady state (constant number) | | | | | | | | | | | |

Thus, this method gives reliable patient number estimates if the incidence is approximately stable or does not change abruptly and that the progression rate of the clinical picture of the disease (, i.e., the sequence 15 – 20 – 50 – 70 – 30 – 15) does not undergo significant abrupt changes. Such changes could be due to recently introduced screening programmes resulting in more patients in earlier stages of disease at first diagnosis or new therapies that affect the time of progression to more advanced stages of disease.

The effect of a change of incidence will be illustrated in the following Table S11b assuming an increasing incidence over time, when it was 20% lower 10 years ago. This is reflected in an evenly increase of the patients from 160 (year -10) to the currently estimated 200 eligible patients (year 0) in the last column. – This is a reasonable scenario as for example the incidence of prostate cancer in the EU-27 is supposed to increase from about 344,000 to 397,000 between 2025 and 2035 (+ 15.4%). – Again, the numbers in Table S11b are only for illustration purposes.

The main result is that an increasing incidence will lead to an overestimate of the eligible patients in the reference year 0 because there were less new patients in the past that could compensate for those 'lost to the future' in the reference year 0. The sum in column year 0 is only 190 instead of the estimated 200.

**Table S11b: Effect of increasing incidence over time. The deviation from a steady state will lead to a slight overestimate (200 instead of 190) of the eligible patients in the reference year 0.**

| | past | | | | | | | | | | reference year | future | | | | | number of eligible patients per year |
|---|---|---|---|---|---|---|---|---|---|---|---|---|---|---|---|---|---|
| **Year** | **-10** | **-9** | **-8** | **-7** | **-6** | **-5** | **-4** | **-3** | **-2** | **-1** | **0** | **1** | **2** | **3** | **4** | **5** | |
| **0** | | | | | | | | | | | 15 | 20 | 50 | 70 | 30 | 15 | 200 |
| **-1** | | | | | | | | | | 14 | 19 | 50 | 70 | 29 | 14 | | 196 |
| **-2** | | | | | | | | | 13 | 19 | 49 | 69 | 29 | 13 | | | 192 |
| **-3** | | | | | | | | 13 | 18 | 48 | 68 | 28 | 13 | | | | 188 |
| **-4** | | | | | | | 12 | 17 | 48 | 68 | 27 | 12 | | | | | 184 |
| **-5** | | | | | | 12 | 16 | 47 | 67 | 26 | 12 | | | | | | 180 |
| **-6** | | | | | 12 | 15 | 46 | 66 | 26 | 11 | | | | | | | 176 |
| **-7** | | | | 11 | 15 | 45 | 65 | 25 | 11 | | | | | | | | 172 |
| **-8** | | | 10 | 14 | 45 | 65 | 25 | 10 | | | | | | | | | 169 |
| **-9** | | 10 | 13 | 43 | 64 | 24 | 10 | | | | | | | | | | 164 |
| **-10** | 9 | 12 | 42 | 63 | 24 | 10 | | | | | | | | | | | 160 |
| | ... | ... | ... | ... | ... | 171 | 174 | 178 | 183 | 186 | 190 | | | | | | |
| | | | | | | no steady state (inreasing number) | | | | | | | | | | | |

In the present work the effect of overestimating due to increasing incidence is mitigated as the increase of the number of eligible patients over time is mainly driven by the extension of marketing authorisations to treat patients earlier in the course of disease and by marketing authorisations for new products treating different diseases. Considering the uncertainties of the parameters taken from literature which are reflected in the upper and lower bound estimates (range in the patient numbers), the uncertainties of the derived patient numbers due to deviations from steady state assumption can be considered comparably small.

The methodology outlined above could be called a “delayed incidence method” as the result for the number of patients eligible for treatment each year contains patients that were incident in the years before.

## Methodology – Calculation schemes and sensitivity analysis for incidence data

Literature data frequently exhibit a large scatter from one source to the other and sometimes even single sources present a crude range of values for example for the progression rate to a certain metastatic stage of cancer. To estimate the size of the patient pools eligible for a certain radioligand therapy in a specific medical condition the scatter of data was considered in a sensitivity analysis combining the lowest values for the lower bound estimate and the highest values for the upper bound estimate. Mathematically, this is a simple multiplication of percentages. The challenge is to find literature data that can be combined in a meaningful way, especially concerning the fraction of patients that undergo a certain line of treatment. Due to the scarcity of data, sometimes data must be combined stemming from different geographical regions and different healthcare systems where national treatment guidelines, habits and reimbursement schemes may affect the results. This is a limitation of the present study.

The following figures give examples for the calculations schemes to derive the patient pool estimates. All calculations can be found in Tables S12-16 and S18-S23 in this Supplementary Material.

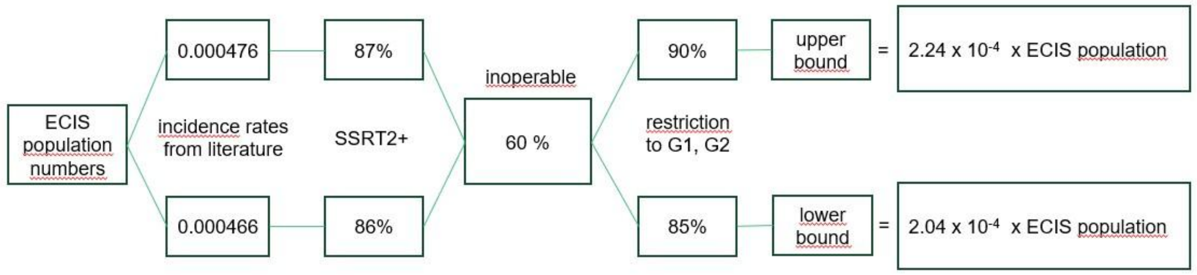


**Figure S3: Calculation scheme applied in Table S12 for GEP-NETs (valid for 2022 and 2025).**

Figure S3 gives an example calculation scheme for GEP-NETs. As they are distributed over various organs, ECIS does not provide adequate incidence numbers. These must be taken from literature and are given with an upper and lower estimate. The number of patients is obtained by multiplying the ECIS population numbers with the incidence-rates, assuming that they are valid for all EU countries. The rate of sstr+ patients is well-known. 60% of the patients exhibit inoperable (including metastatic) cancer and 85%-90% have the histology grading G1 and G2 (this restriction may be lifted for 2030 and 2035). Multiplying the lower bound numbers yields the lower bound estimate; analogously one proceeds for the upper bound estimate. Numeric results and data sources are presented in Table S12.

Figures S4a and S4b outline the calculation scheme for the patient pool eligible for various treatment lines of mCRPC and for mHSPC, respectivey. The data sources and the numerical results are presented in Table S16. ECIS provides the number of incident patients. Only those which are PSMA+ will have a chance to respond to therapy. The initial marketing authorisation restricts $^{177}$Lu-PSMA therapy to 3$^{rd}$ line treatment (3L) of metastatic castration resistant prostate cancer (mCRPC). It is expected that this will stepwise be extended over time for 2$^{nd}$ line (2L) and first line (1L). Literature data used for 2$^{nd}$ and 3$^{rd}$ line treatment are averaged over several countries, but no similar literature sources have been found. The calculation is depicted in Figure S4a, the numerical results (colour coded) in Table S16.

Figure S4b depicts the calculations for the maximum pool size of patients treated with metastatic hormone sensitive prostate cancer (mHSPC). The progression rate of prostate cancer patients to the mHSPC state is given in literature with 30-40%. These patients will eventually all transition to the mCRPC state and the intention to treat these with $^{177}$Lu-PSMA is to delay or ideally avoid this transition, while avoiding also the cardiovascular and other undesired side effects of Androgen Deprivation Therapy. The transition from the mHSPC state to the mCRPC can be considered a linear development of disease Thus, this calculation with 30-40% of patients developing mHSPC covers already the 10-20% of patients which may become mCRPC at a later timepoint or not if $^{177}$Lu-PSMA therapy avoids mCRPC before the patients die due to age-related mortality.

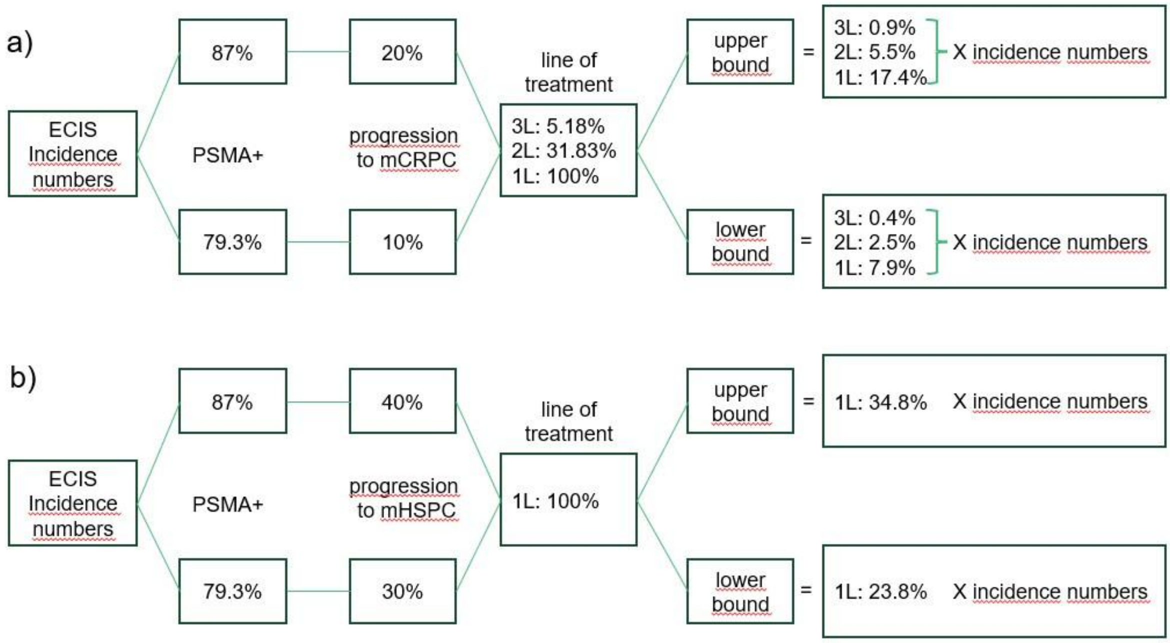


**Figure S4: Calculation scheme applied in Table S16 for the sensitivity analysis to estimate the pool size of prostate cancer patients in the mCRPC state (a) and the mHSPC state (b). Data sources are given in Table S16.**

Figure S5 shows the calculation scheme for the sensitivity analysis for the clear cell Renal Cell Carcinoma (ccRCC) patient pool. The data sources are reported in Table S21. ECIS provides the number of kidney cancer incidences per year, which has to be reduced to the subtype of ccRCC. For the envisaged therapy only patients which are CAIX (CA-9) positive have a chance benefit from therapy. 13% of the ccRCC patients have metastatic cancer at first diagnosis. First line therapy is usually total or partial nephrectomy, and 50% of the patients are at risk to develop metastatic disease later. Thus, a total of 56.5% of the patient may exhibit metastatic disease during the course of their cancer. However, the envisaged therapy uses an antibody (girentuximab) as vector that has a long residence time in blood with a high risk of developing myelotoxicity. Thus, only a small fraction of the mccRCC patients may benefit from therapy. Finally, only 3.1% to 8% of the kidney cancer patients might benefit from this type of radioligand therapy.

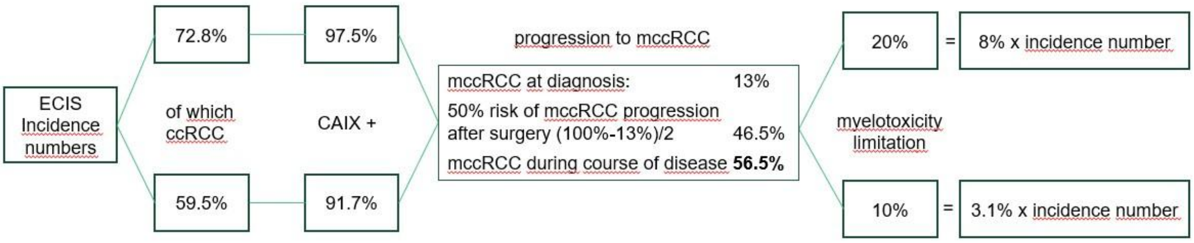


**Figure S5: Calculation scheme applied in Table S21 for the sensitivity analysis to estimate the maximum pools size of patients with clear cell Renal Cell Carcinoma (ccRCC) that might benefit from $^{177}$Lu-girentuximab radioligand therapy.**

## Table S12: Estimate of the pool of sstr2+ GEP-NET patients eligible for radioligand therapy by authorisation criteria in the EU-27 based on ECIS population data

| Parameters considered in the estimate | range | | | |
|---|---|---|---|---|
| Incidence rate of GEP-NETS (1,2) | 0.0000466 | 0.0000476 | | |
| prevalence of somatostatin receptor expression (3,4) | 0.86 | 0.87 | for lower range estimate | 2.40456E-05 |
| fraction of patients with inoperable GEP-NETs (5) | 0.6 | 0.6 | for upper range estimate | 2.48472E-05 |
| restriction to G1 and G2 ONLY for 2022 and 2025 (6) | 0.85 | 0.9 | | |


1) White et al. (2022) Incidence and survival of neuroendocrine neoplasia in England 1995-2018: A retrospective, population-based study. Lancet Reg Health Eur. 2022 Sep 23;23:100510. doi: 10.1016/j.lanepe.2022.100510

2) Grundmann et al. (2023) Site-specific trends in gastroenteropancreatic neuroendocrine neoplasms in Bavaria, Germany. Cancer Med. 2023 Oct;12(19):19949-19958. doi: 10.1002/cam4.6510

3) Oberg et al. (2004) Consensus report on the use of somatostatin analogs for the management of neuroendocrine tumors of the gastroenteropancreatic system. Ann Oncol. 2004 Jun;15(6):966-73. doi: 10.1093/annonc/mdh216

4) Childs et al. (2016) Expression of somatostatin receptors 2 and 5 in circulating tumour cells from patients with neuroendocrine tumours. Br J Cancer. 2016 Dec 6;115(12):1540-1547. doi: 10.1038/bjc.2016.377

5) Capelli et al. (2012) Pathology - grading and staging of GEP-NETs. Best Pract Res Clin Gastroenterol. 2012 Dec;26(6):705-17. doi: 10.1016/j.bpg.2013.01.003

6) Mitjavila et al. (2023) Efficacy of [$^{177}$Lu]Lu-DOTATATE in metastatic neuroendocrine neoplasms of different locations: data from the SEPTRALU study. Eur J Nucl Med Mol Imaging. 2023 Jul;50(8):2486-2500. doi: 10.1007/s00259-023-06166-8


| | Population 2022 and projections | | | | lower range estimate | | | | upper range estimate | | | |
|---|---|---|---|---|---|---|---|---|---|---|---|---|
| Country | 2022 | 2025 | 2030 | 2035 | 2022 | 2025 | 2030 | 2035 | 2022 | 2025 | 2030 | 2035 |
| Austria | 8,978,929 | 9,111,243 | 9,214,690 | 9,319,086 | 184 | 186 | 222 | 224 | 201 | 204 | 229 | 232 |
| Belgium | 11,617,623 | 11,829,411 | 12,009,045 | 12,179,830 | 237 | 242 | 289 | 293 | 260 | 265 | 298 | 303 |
| Bulgaria | 6,838,937 | 6,860,349 | 6,574,153 | 6,333,689 | 140 | 140 | 158 | 152 | 153 | 153 | 163 | 157 |
| Croatia | 3,862,305 | 3,810,628 | 3,693,206 | 3,593,292 | 79 | 78 | 89 | 86 | 86 | 85 | 92 | 89 |
| Cyprus | 904,705 | 941,765 | 957,744 | 967,207 | 18 | 19 | 23 | 23 | 20 | 21 | 24 | 24 |
| Czechia | 10,516,707 | 11,017,341 | 10,851,301 | 10,728,942 | 215 | 225 | 261 | 258 | 235 | 246 | 270 | 267 |
| Denmark | 5,873,420 | 5,979,924 | 6,059,699 | 6,112,281 | 120 | 122 | 146 | 147 | 131 | 134 | 151 | 152 |
| Estonia | 1,331,796 | 1,377,519 | 1,358,611 | 1,344,440 | 27 | 28 | 33 | 32 | 30 | 31 | 34 | 33 |
| EU-27 | 446,735,291 | 453,168,040 | 452,700,101 | 451,991,345 | 9131 | 9262 | 10885 | 10868 | 9990 | 10134 | 11248 | 11231 |
| Finland | 5,548,241 | 5,640,423 | 5,631,487 | 5,601,455 | 113 | 115 | 135 | 135 | 124 | 126 | 140 | 139 |
| France | 67,871,925 | 68,658,223 | 69,386,211 | 70,026,306 | 1387 | 1403 | 1668 | 1684 | 1518 | 1535 | 1724 | 1740 |
| Germany | 83,237,124 | 85,207,514 | 85,284,256 | 85,216,229 | 1701 | 1742 | 2051 | 2049 | 1861 | 1905 | 2119 | 2117 |
| Greece | 10,459,782 | 10,320,364 | 10,032,545 | 9,758,893 | 214 | 211 | 241 | 235 | 234 | 231 | 249 | 242 |
| Hungary | 9,689,010 | 9,644,847 | 9,526,758 | 9,422,235 | 198 | 197 | 229 | 227 | 217 | 216 | 237 | 234 |
| Ireland | 5,060,004 | 5,257,383 | 5,416,927 | 5,579,300 | 103 | 107 | 130 | 134 | 113 | 118 | 135 | 139 |
| Italy | 59,030,133 | 58,951,070 | 58,773,783 | 58,655,761 | 1207 | 1205 | 1413 | 1410 | 1320 | 1318 | 1460 | 1457 |
| Latvia | 1,875,757 | 1,863,089 | 1,756,334 | 1,660,761 | 38 | 38 | 42 | 40 | 42 | 42 | 44 | 41 |
| Lithuania | 2,805,998 | 2,860,472 | 2,741,927 | 2,622,099 | 57 | 58 | 66 | 63 | 63 | 64 | 68 | 65 |
| Luxembourg | 645,397 | 687,081 | 740,420 | 788,408 | 13 | 14 | 18 | 19 | 14 | 15 | 18 | 20 |
| Malta | 520,971 | 553,623 | 604,727 | 649,012 | 11 | 11 | 15 | 16 | 12 | 12 | 15 | 16 |
| Netherlands | 17,590,672 | 18,048,588 | 18,341,701 | 18,564,556 | 360 | 369 | 441 | 446 | 393 | 404 | 456 | 461 |
| Poland | 37,654,247 | 38,381,332 | 37,420,524 | 36,517,358 | 770 | 784 | 900 | 878 | 842 | 858 | 930 | 907 |
| Portugal | 10,352,042 | 10,372,141 | 10,249,138 | 10,120,798 | 212 | 212 | 246 | 243 | 231 | 232 | 255 | 251 |
| Romania | 19,042,455 | 18,831,698 | 18,218,553 | 17,683,694 | 389 | 385 | 438 | 425 | 426 | 421 | 453 | 439 |
| Slovakia | 5,434,712 | 5,521,368 | 5,450,183 | 5,368,574 | 111 | 113 | 131 | 129 | 122 | 123 | 135 | 133 |
| Slovenia | 2,107,180 | 2,120,770 | 2,118,806 | 2,113,672 | 43 | 43 | 51 | 51 | 47 | 47 | 53 | 53 |
| Spain | 47,432,893 | 48,614,060 | 49,266,930 | 49,760,920 | 969 | 994 | 1185 | 1197 | 1061 | 1087 | 1224 | 1236 |
| Sweden | 10,452,326 | 10,705,814 | 11,020,442 | 11,302,547 | 214 | 219 | 265 | 272 | 234 | 239 | 274 | 281 |

Source: ECIS - European Cancer Information System
From https://ecis.jrc.ec.europa.eu/
(C) European union

| | Population 2022 and projections | | | | lower range estimate | | | | upper range estimate | | | |
|---|---|---|---|---|---|---|---|---|---|---|---|---|
| Country | 2022 | 2025 | 2030 | 2035 | 2022 | 2025 | 2030 | 2025 | 2022 | 2025 | 2030 | 2025 |
| EU-27 | 446,735,291 | 453,168,040 | 452,700,101 | 451,991,345 | 10742 | 10897 | 10885 | 10868 | 11100 | 11260 | 11248 | 11231 |
| France | 67,871,925 | 68,658,223 | 69,386,211 | 70,026,306 | 1632 | 1651 | 1668 | 1684 | 1686 | 1706 | 1724 | 1740 |
| Germany | 83,237,124 | 85,207,514 | 85,284,256 | 85,216,229 | 2001 | 2049 | 2051 | 2049 | 2068 | 2117 | 2119 | 2117 |
| Italy | 59,030,133 | 58,951,070 | 58,773,783 | 58,655,761 | 1419 | 1418 | 1413 | 1410 | 1467 | 1465 | 1460 | 1457 |
| Spain | 47,432,893 | 48,614,060 | 49,266,930 | 49,760,920 | 1141 | 1169 | 1185 | 1197 | 1179 | 1208 | 1224 | 1236 |
| EU-4 | 257572075 | 261430867 | 262711180 | 263659216 | 6193 | 6286 | 6317 | 6340 | 6400 | 6496 | 6528 | 6551 |

## Table S13: Estimate of the pool of sstr+ lung-NET patients eligible for radioligand therapy by conjectured authorisation criteria in the EU-27 based on ECIS population data

| Parameters considered in the estimate | range | | | |
|---|---|---|---|---|
| Incidence rate of Lung-NETS (1) | 0.0000147 | 0.0000147 | | |
| prevalence of somatostatin receptor expression (2) | 0.75 | 0.75 | for lower range estimate | 0.000011025 |
| fraction of patients with inoperable Lung-NETs | 1 | 1 | for upper range estimate | 0.000011025 |


1) White et al. (2022) Incidence and survival of neuroendocrine neoplasia in England 1995-2018: A retrospective, population-based study. Lancet Reg Health Eur. 2022 Sep 23;23:100510. doi: 10.1016/j.lanepe.2022.100510

2) Remes et al- (2019) Immunohistochemical Expression of Somatostatin Receptor Subtypes in a Panel of Neuroendocrine Neoplasias. J Histochem Cytochem. 2019 Oct;67(10):735-743. doi: 10.1369/0022155419856900


| | Population 2022 and projections | | | | lower range estimate | | | | upper range estimate | | | |
|---|---|---|---|---|---|---|---|---|---|---|---|---|
| Country | 2022 | 2025 | 2030 | 2035 | 2022 | 2025 | 2030 | 2035 | 2022 | 2025 | 2030 | 2035 |
| Austria | 8,978,929 | 9,111,243 | 9,214,690 | 9,319,086 | 99 | 100 | 102 | 103 | 99 | 100 | 102 | 103 |
| Belgium | 11,617,623 | 11,829,411 | 12,009,045 | 12,179,830 | 128 | 130 | 132 | 134 | 128 | 130 | 132 | 134 |
| Bulgaria | 6,838,937 | 6,860,349 | 6,574,153 | 6,333,689 | 75 | 76 | 72 | 70 | 75 | 76 | 72 | 70 |
| Croatia | 3,862,305 | 3,810,628 | 3,693,206 | 3,593,292 | 43 | 42 | 41 | 40 | 43 | 42 | 41 | 40 |
| Cyprus | 904,705 | 941,765 | 957,744 | 967,207 | 10 | 10 | 11 | 11 | 10 | 10 | 11 | 11 |
| Czechia | 10,516,707 | 11,017,341 | 10,851,301 | 10,728,942 | 116 | 121 | 120 | 118 | 116 | 121 | 120 | 118 |
| Denmark | 5,873,420 | 5,979,924 | 6,059,699 | 6,112,281 | 65 | 66 | 67 | 67 | 65 | 66 | 67 | 67 |
| Estonia | 1,331,796 | 1,377,519 | 1,358,611 | 1,344,440 | 15 | 15 | 15 | 15 | 15 | 15 | 15 | 15 |
| EU-27 | 446,735,291 | 453,168,040 | 452,700,101 | 451,991,345 | 4925 | 4996 | 4991 | 4983 | 4925 | 4996 | 4991 | 4983 |
| Finland | 5,548,241 | 5,640,423 | 5,631,487 | 5,601,455 | 61 | 62 | 62 | 62 | 61 | 62 | 62 | 62 |
| France | 67,871,925 | 68,658,223 | 69,386,211 | 70,026,306 | 748 | 757 | 765 | 772 | 748 | 757 | 765 | 772 |
| Germany | 83,237,124 | 85,207,514 | 85,284,256 | 85,216,229 | 918 | 939 | 940 | 940 | 918 | 939 | 940 | 940 |
| Greece | 10,459,782 | 10,320,364 | 10,032,545 | 9,758,893 | 115 | 114 | 111 | 108 | 115 | 114 | 111 | 108 |
| Hungary | 9,689,010 | 9,644,847 | 9,526,758 | 9,422,235 | 107 | 106 | 105 | 104 | 107 | 106 | 105 | 104 |
| Ireland | 5,060,004 | 5,257,383 | 5,416,927 | 5,579,300 | 56 | 58 | 60 | 62 | 56 | 58 | 60 | 62 |
| Italy | 59,030,133 | 58,951,070 | 58,773,783 | 58,655,761 | 651 | 650 | 648 | 647 | 651 | 650 | 648 | 647 |
| Latvia | 1,875,757 | 1,863,089 | 1,756,334 | 1,660,761 | 21 | 21 | 19 | 18 | 21 | 21 | 19 | 18 |
| Lithuania | 2,805,998 | 2,860,472 | 2,741,927 | 2,622,099 | 31 | 32 | 30 | 29 | 31 | 32 | 30 | 29 |
| Luxembourg | 645,397 | 687,081 | 740,420 | 788,408 | 7 | 8 | 8 | 9 | 7 | 8 | 8 | 9 |
| Malta | 520,971 | 553,623 | 604,727 | 649,012 | 6 | 6 | 7 | 7 | 6 | 6 | 7 | 7 |
| Netherlands | 17,590,672 | 18,048,588 | 18,341,701 | 18,564,556 | 194 | 199 | 202 | 205 | 194 | 199 | 202 | 205 |
| Poland | 37,654,247 | 38,381,332 | 37,420,524 | 36,517,358 | 415 | 423 | 413 | 403 | 415 | 423 | 413 | 403 |
| Portugal | 10,352,042 | 10,372,141 | 10,249,138 | 10,120,798 | 114 | 114 | 113 | 112 | 114 | 114 | 113 | 112 |
| Romania | 19,042,455 | 18,831,698 | 18,218,553 | 17,683,694 | 210 | 208 | 201 | 195 | 210 | 208 | 201 | 195 |
| Slovakia | 5,434,712 | 5,521,368 | 5,450,183 | 5,368,574 | 60 | 61 | 60 | 59 | 60 | 61 | 60 | 59 |
| Slovenia | 2,107,180 | 2,120,770 | 2,118,806 | 2,113,672 | 23 | 23 | 23 | 23 | 23 | 23 | 23 | 23 |
| Spain | 47,432,893 | 48,614,060 | 49,266,930 | 49,760,920 | 523 | 536 | 543 | 549 | 523 | 536 | 543 | 549 |
| Sweden | 10,452,326 | 10,705,814 | 11,020,442 | 11,302,547 | 115 | 118 | 122 | 125 | 115 | 118 | 122 | 125 |

Source: ECIS - European Cancer Information System
From https://ecis.jrc.ec.europa.eu/
(C) European union

| | Population 2022 and projections | | | | lower range estimate | | | | upper range estimate | | | |
|---|---|---|---|---|---|---|---|---|---|---|---|---|
| Country | 2022 | 2025 | 2030 | 2035 | 2022 | 2025 | 2030 | 2025 | 2022 | 2025 | 2030 | 2025 |
| EU-27 | 446,735,291 | 453,168,040 | 452,700,101 | 451,991,345 | 4925 | 4996 | 4991 | 4983 | 4925 | 4996 | 4991 | 4983 |
| France | 67,871,925 | 68,658,223 | 69,386,211 | 70,026,306 | 748 | 757 | 765 | 772 | 748 | 757 | 765 | 772 |
| Germany | 83,237,124 | 85,207,514 | 85,284,256 | 85,216,229 | 918 | 939 | 940 | 940 | 918 | 939 | 940 | 940 |
| Italy | 59,030,133 | 58,951,070 | 58,773,783 | 58,655,761 | 651 | 650 | 648 | 647 | 651 | 650 | 648 | 647 |
| Spain | 47,432,893 | 48,614,060 | 49,266,930 | 49,760,920 | 523 | 536 | 543 | 549 | 523 | 536 | 543 | 549 |
| EU-big 4 | 257,572,075 | 261,430,867 | 262,711,180 | 263,659,216 | | | 2896 | 2907 | | | 2896 | 2907 |

**Table S14: Patient pool estimate of sstr+ pheochromocytoma and paraganglioma patients eligible for RLT by conjectured authorisation criteria (based on clinical trials) in the EU-27 based on ECIS population data**

| Parameters considered in the estimate | range | | | |
|---|---|---|---|---|
| Incidence rate for Pheochromocytoma and Paraganlioma (1) | 0.00000417 | 0.00000661 | | |
| prevalence of somatostatin receptor expression (2) | 0.748 | 0.748 | for lower range estimate | 3.11916E-06 |
| fraction of patients with inoperable tumours | 1 | 1 | for upper range estimate | 4.94428E-06 |

1) Al Subhi et al. (2022) Systematic Review: Incidence of Pheochromocytoma and Paraganglioma Over 70 Years. J Endocr Soc. 2022 Jul 3;6(9):bvac105. doi: 10.1210/jendso/bvac105

2) Leijon et al. (2019) Variable somatostatin receptor subtype expression in 151 primary pheochromocytomas and paragangliomas. Hum Pathol. 2019 Apr;86:66-75. doi: 10.1016/j.humpath.2018.11.020

| | Population 2022 and projections | | | | lower range estimate | | | | upper range estimate | | | |
|---|---|---|---|---|---|---|---|---|---|---|---|---|
| Country | 2022 | 2025 | 2030 | 2035 | 2022 | 2025 | 2030 | 2035 | 2022 | 2025 | 2030 | 2035 |
| Austria | 8,978,929 | 9,111,243 | 9,214,690 | 9,319,086 | 28 | 28 | 29 | 29 | 44 | 45 | 46 | 46 |
| Belgium | 11,617,623 | 11,829,411 | 12,009,045 | 12,179,830 | 36 | 37 | 37 | 38 | 57 | 58 | 59 | 60 |
| Bulgaria | 6,838,937 | 6,860,349 | 6,574,153 | 6,333,689 | 21 | 21 | 21 | 20 | 34 | 34 | 33 | 31 |
| Croatia | 3,862,305 | 3,810,628 | 3,693,206 | 3,593,292 | 12 | 12 | 12 | 11 | 19 | 19 | 18 | 18 |
| Cyprus | 904,705 | 941,765 | 957,744 | 967,207 | 3 | 3 | 3 | 3 | 4 | 5 | 5 | 5 |
| Czechia | 10,516,707 | 11,017,341 | 10,851,301 | 10,728,942 | 33 | 34 | 34 | 33 | 52 | 54 | 54 | 53 |
| Denmark | 5,873,420 | 5,979,924 | 6,059,699 | 6,112,281 | 18 | 19 | 19 | 19 | 29 | 30 | 30 | 30 |
| Estonia | 1,331,796 | 1,377,519 | 1,358,611 | 1,344,440 | 4 | 4 | 4 | 4 | 7 | 7 | 7 | 7 |
| EU-27 | 446,735,291 | 453,168,040 | 452,700,101 | 451,991,345 | 1393 | 1414 | 1412 | 1410 | 2209 | 2241 | 2238 | 2235 |
| Finland | 5,548,241 | 5,640,423 | 5,631,487 | 5,601,455 | 17 | 18 | 18 | 17 | 27 | 28 | 28 | 28 |
| France | 67,871,925 | 68,658,223 | 69,386,211 | 70,026,306 | 212 | 214 | 216 | 218 | 336 | 339 | 343 | 346 |
| Germany | 83,237,124 | 85,207,514 | 85,284,256 | 85,216,229 | 260 | 266 | 266 | 266 | 412 | 421 | 422 | 421 |
| Greece | 10,459,782 | 10,320,364 | 10,032,545 | 9,758,893 | 33 | 32 | 31 | 30 | 52 | 51 | 50 | 48 |
| Hungary | 9,689,010 | 9,644,847 | 9,526,758 | 9,422,235 | 30 | 30 | 30 | 29 | 48 | 48 | 47 | 47 |
| Ireland | 5,060,004 | 5,257,383 | 5,416,927 | 5,579,300 | 16 | 16 | 17 | 17 | 25 | 26 | 27 | 28 |
| Italy | 59,030,133 | 58,951,070 | 58,773,783 | 58,655,761 | 184 | 184 | 183 | 183 | 292 | 291 | 291 | 290 |
| Latvia | 1,875,757 | 1,863,089 | 1,756,334 | 1,660,761 | 6 | 6 | 5 | 5 | 9 | 9 | 9 | 8 |
| Lithuania | 2,805,998 | 2,860,472 | 2,741,927 | 2,622,099 | 9 | 9 | 9 | 8 | 14 | 14 | 14 | 13 |
| Luxembourg | 645,397 | 687,081 | 740,420 | 788,408 | 2 | 2 | 2 | 2 | 3 | 3 | 4 | 4 |
| Malta | 520,971 | 553,623 | 604,727 | 649,012 | 2 | 2 | 2 | 2 | 3 | 3 | 3 | 3 |
| Netherlands | 17,590,672 | 18,048,588 | 18,341,701 | 18,564,556 | 55 | 56 | 57 | 58 | 87 | 89 | 91 | 92 |
| Poland | 37,654,247 | 38,381,332 | 37,420,524 | 36,517,358 | 117 | 120 | 117 | 114 | 186 | 190 | 185 | 181 |
| Portugal | 10,352,042 | 10,372,141 | 10,249,138 | 10,120,798 | 32 | 32 | 32 | 32 | 51 | 51 | 51 | 50 |
| Romania | 19,042,455 | 18,831,698 | 18,218,553 | 17,683,694 | 59 | 59 | 57 | 55 | 94 | 93 | 90 | 87 |
| Slovakia | 5,434,712 | 5,521,368 | 5,450,183 | 5,368,574 | 17 | 17 | 17 | 17 | 27 | 27 | 27 | 27 |
| Slovenia | 2,107,180 | 2,120,770 | 2,118,806 | 2,113,672 | 7 | 7 | 7 | 7 | 10 | 10 | 10 | 10 |
| Spain | 47,432,893 | 48,614,060 | 49,266,930 | 49,760,920 | 148 | 152 | 154 | 155 | 235 | 240 | 244 | 246 |
| Sweden | 10,452,326 | 10,705,814 | 11,020,442 | 11,302,547 | 33 | 33 | 34 | 35 | 52 | 53 | 54 | 56 |

Source: ECIS - European Cancer Information System
From https://ecis.jrc.ec.europa.eu/
(C) European union

| | Population 2022 and projections | | | | lower range estimate | | | | upper range estimate | | | |
|---|---|---|---|---|---|---|---|---|---|---|---|---|
| Country | 2022 | 2025 | 2030 | 2035 | 2022 | 2025 | 2030 | 2025 | 2022 | 2025 | 2030 | 2025 |
| EU-27 | 446,735,291 | 453,168,040 | 452,700,101 | 451,991,345 | 1393 | 1414 | 1412 | 1410 | 2209 | 2241 | 2238 | 2235 |
| France | 67,871,925 | 68,658,223 | 69,386,211 | 70,026,306 | 212 | 214 | 216 | 218 | 336 | 339 | 343 | 346 |
| Germany | 83,237,124 | 85,207,514 | 85,284,256 | 85,216,229 | 260 | 266 | 266 | 266 | 412 | 421 | 422 | 421 |
| Italy | 59,030,133 | 58,951,070 | 58,773,783 | 58,655,761 | 184 | 184 | 183 | 183 | 292 | 291 | 291 | 290 |
| Spain | 47,432,893 | 48,614,060 | 49,266,930 | 49,760,920 | 148 | 152 | 154 | 155 | 235 | 240 | 244 | 246 |
| EU-4 | | | | | | | 819 | 822 | | | 1299 | 1304 |

**Table S15: Estimated overall evolution of the patient pool for patients with sstr2+ GEP-NETs, lung NETS, pheochromocytoma and paraganglioma (PPGL) eligible for radioligand therapies according to current and conjectured future authorisation criteria in the EU-27 based on ECIS population data**

| | GEP-NETs authorised in EU from 2017 on | | | | | | | | lung NETs - off label use might become authorised in 2025 | | | | | | | | PPGL- NETs - off-label use might be authorised by 2030 | | | | | | | | GEP-NET & lung & PPGL | | | | GEP-NET & lung & PPGL | | | | |
|---|---|---|---|---|---|---|---|---|---|---|---|---|---|---|---|---|---|---|---|---|---|---|---|---|---|---|---|---|---|---|---|---|---|
| | lower range estimate | | | | upper range estimate | | | | lower range estimate | | | | upper range estimate | | | | lower range estimate | | | | upper range estimate | | | | lower ALL NET | | | | upper ALL NET | | | | |
| Country | 2022 | 2025 | 2030 | 2035 | 2022 | 2025 | 2030 | 2035 | 2022 | 2025 | 2030 | 2035 | 2022 | 2025 | 2030 | 2035 | 2022 | 2025 | 2030 | 2035 | 2022 | 2025 | 2030 | 2035 | 2022 | 2025 | 2030 | 2035 | 2022 | 2025 | 2030 | 2035 | Country |
| Austria | 184 | 186 | 222 | 224 | 201 | 204 | 229 | 232 | | | 102 | 103 | | | 102 | 103 | | | 29 | 29 | | | 46 | 46 | 184 | 186 | 352 | 356 | 201 | 204 | 376 | 380 | Austria |
| Belgium | 237 | 242 | 289 | 293 | 260 | 265 | 298 | 303 | | | 132 | 134 | | | 132 | 134 | | | 37 | 38 | | | 59 | 60 | 237 | 242 | 459 | 465 | 260 | 265 | 490 | 497 | Belgium |
| Bulgaria | 140 | 140 | 158 | 152 | 153 | 153 | 163 | 157 | | | 72 | 70 | | | 72 | 70 | | | 21 | 20 | | | 33 | 31 | 140 | 140 | 251 | 242 | 153 | 153 | 268 | 259 | Bulgaria |
| Croatia | 79 | 78 | 89 | 86 | 86 | 85 | 92 | 89 | | | 41 | 40 | | | 41 | 40 | | | 12 | 11 | | | 18 | 18 | 79 | 78 | 141 | 137 | 86 | 85 | 151 | 147 | Croatia |
| Cyprus | 18 | 19 | 23 | 23 | 20 | 21 | 24 | 24 | | | 11 | 11 | | | 11 | 11 | | | 3 | 3 | | | 5 | 5 | 18 | 19 | 37 | 37 | 20 | 21 | 39 | 39 | Cyprus |
| Czechia | 215 | 225 | 261 | 258 | 235 | 246 | 270 | 267 | | | 120 | 118 | | | 120 | 118 | | | 34 | 33 | | | 54 | 53 | 215 | 225 | 414 | 410 | 235 | 246 | 443 | 438 | Czechia |
| Denmark | 120 | 122 | 146 | 147 | 131 | 134 | 151 | 152 | | | 67 | 67 | | | 67 | 67 | | | 19 | 19 | | | 30 | 30 | 120 | 122 | 231 | 233 | 131 | 134 | 247 | 249 | Denmark |
| Estonia | 27 | 28 | 33 | 32 | 30 | 31 | 34 | 33 | | | 15 | 15 | | | 15 | 15 | | | 4 | 4 | | | 7 | 7 | 27 | 28 | 52 | 51 | 30 | 31 | 55 | 55 | Estonia |
| EU-27 | 9131 | 9262 | 10885 | 10868 | 9990 | 10134 | 11248 | 11231 | | | 4991 | 4983 | | | 4991 | 4983 | | | 1412 | 1410 | | | 2238 | 2235 | 9131 | 9262 | 17289 | 17261 | 9990 | 10134 | 18478 | 18449 | EU-27 |
| Finland | 113 | 115 | 135 | 135 | 124 | 126 | 140 | 139 | | | 62 | 62 | | | 62 | 62 | | | 18 | 17 | | | 28 | 28 | 113 | 115 | 215 | 214 | 124 | 126 | 230 | 229 | Finland |
| France | 1387 | 1403 | 1668 | 1684 | 1518 | 1535 | 1724 | 1740 | | | 765 | 772 | | | 765 | 772 | | | 216 | 218 | | | 343 | 346 | 1387 | 1403 | 2650 | 2674 | 1518 | 1535 | 2832 | 2858 | France |
| Germany | 1701 | 1742 | 2051 | 2049 | 1861 | 1905 | 2119 | 2117 | | | 940 | 940 | | | 940 | 940 | | | 266 | 266 | | | 422 | 421 | 1701 | 1742 | 3257 | 3254 | 1861 | 1905 | 3481 | 3478 | Germany |
| Greece | 214 | 211 | 241 | 235 | 234 | 231 | 249 | 242 | | | 111 | 108 | | | 111 | 108 | | | 31 | 30 | | | 50 | 48 | 214 | 211 | 383 | 373 | 234 | 231 | 409 | 398 | Greece |
| Hungary | 198 | 197 | 229 | 227 | 217 | 216 | 237 | 234 | | | 105 | 104 | | | 105 | 104 | | | 30 | 29 | | | 47 | 47 | 198 | 197 | 364 | 360 | 217 | 216 | 389 | 385 | Hungary |
| Ireland | 103 | 107 | 130 | 134 | 113 | 118 | 135 | 139 | | | 60 | 62 | | | 60 | 62 | | | 17 | 17 | | | 27 | 28 | 103 | 107 | 207 | 213 | 113 | 118 | 221 | 228 | Ireland |
| Italy | 1207 | 1205 | 1413 | 1410 | 1320 | 1318 | 1460 | 1457 | | | 648 | 647 | | | 648 | 647 | | | 183 | 183 | | | 291 | 290 | 1207 | 1205 | 2245 | 2240 | 1320 | 1318 | 2399 | 2394 | Italy |
| Latvia | 38 | 38 | 42 | 40 | 42 | 42 | 44 | 41 | | | 19 | 18 | | | 19 | 18 | | | 5 | 5 | | | 9 | 8 | 38 | 38 | 67 | 63 | 42 | 42 | 72 | 68 | Latvia |
| Lithuania | 57 | 58 | 66 | 63 | 63 | 64 | 68 | 65 | | | 30 | 29 | | | 30 | 29 | | | 9 | 8 | | | 14 | 13 | 57 | 58 | 105 | 100 | 63 | 64 | 112 | 107 | Lithuania |
| Luxembourg | 13 | 14 | 18 | 19 | 14 | 15 | 18 | 20 | | | 8 | 9 | | | 8 | 9 | | | 2 | 2 | | | 4 | 4 | 13 | 14 | 28 | 30 | 14 | 15 | 30 | 32 | Luxembourg |
| Malta | 11 | 11 | 15 | 16 | 12 | 12 | 15 | 16 | | | 7 | 7 | | | 7 | 7 | | | 2 | 2 | | | 3 | 3 | 11 | 11 | 23 | 25 | 12 | 12 | 25 | 26 | Malta |
| Netherlands | 360 | 369 | 441 | 446 | 393 | 404 | 456 | 461 | | | 202 | 205 | | | 202 | 205 | | | 57 | 58 | | | 91 | 92 | 360 | 369 | 700 | 709 | 393 | 404 | 749 | 758 | Netherlands |
| Poland | 770 | 784 | 900 | 878 | 842 | 858 | 930 | 907 | | | 413 | 403 | | | 413 | 403 | | | 117 | 114 | | | 185 | 181 | 770 | 784 | 1429 | 1395 | 842 | 858 | 1527 | 1491 | Poland |
| Portugal | 212 | 212 | 246 | 243 | 231 | 232 | 255 | 251 | | | 113 | 112 | | | 113 | 112 | | | 32 | 32 | | | 51 | 50 | 212 | 212 | 391 | 387 | 231 | 232 | 418 | 413 | Portugal |
| Romania | 389 | 385 | 438 | 425 | 426 | 421 | 453 | 439 | | | 201 | 195 | | | 201 | 195 | | | 57 | 55 | | | 90 | 87 | 389 | 385 | 696 | 675 | 426 | 421 | 744 | 722 | Romania |
| Slovakia | 111 | 113 | 131 | 129 | 122 | 123 | 135 | 133 | | | 60 | 59 | | | 60 | 59 | | | 17 | 17 | | | 27 | 27 | 111 | 113 | 208 | 205 | 122 | 123 | 222 | 219 | Slovakia |
| Slovenia | 43 | 43 | 51 | 51 | 47 | 47 | 53 | 53 | | | 23 | 23 | | | 23 | 23 | | | 7 | 7 | | | 10 | 10 | 43 | 43 | 81 | 81 | 47 | 47 | 86 | 86 | Slovenia |
| Spain | 969 | 994 | 1185 | 1197 | 1061 | 1087 | 1224 | 1236 | | | 543 | 549 | | | 543 | 549 | | | 154 | 155 | | | 244 | 246 | 969 | 994 | 1881 | 1900 | 1061 | 1087 | 2011 | 2031 | Spain |
| Sweden | 214 | 219 | 265 | 272 | 234 | 239 | 274 | 281 | | | 122 | 125 | | | 122 | 125 | | | 34 | 35 | | | 54 | 56 | 214 | 219 | 421 | 432 | 234 | 239 | 450 | 461 | Sweden |

Source: ECIS - European Cancer Information System

From https://ecis.jrc.ec.europa.eu/

(C) European union

| | | | | | | | | | | | | | | | | | | | | | | | | lower bound | | | | upper bound | | | | |
|---|---|---|---|---|---|---|---|---|---|---|---|---|---|---|---|---|---|---|---|---|---|---|---|---|---|---|---|---|---|---|---|---|
| Country | 2022 | 2025 | 2030 | 2035 | 2022 | 2025 | 2030 | 2035 | 2022 | 2025 | 2030 | 2035 | 2022 | 2025 | 2030 | 2035 | 2022 | 2025 | 2030 | 2035 | 2022 | 2025 | 2030 | 2035 | 2022 | 2025 | 2030 | 2035 | 2022 | 2025 | 2030 | 2035 | Country |
| EU-27 | 9131 | 9262 | 10885 | 10868 | 9990 | 10134 | 11248 | 11231 | 0 | 0 | 4991 | 4983 | 0 | 0 | 4991 | 4983 | 0 | 0 | 1412 | 1410 | 0 | 0 | 2238 | 2235 | 9131 | 9262 | 17289 | 17261 | 9990 | 10134 | 18478 | 18449 | **EU-27** |
| France | 1387 | 1403 | 1668 | 1684 | 1518 | 1535 | 1724 | 1740 | 0 | 0 | 765 | 772 | 0 | 0 | 765 | 772 | 0 | 0 | 216 | 218 | 0 | 0 | 343 | 346 | 1387 | 1403 | 2650 | 2674 | 1518 | 1535 | 2832 | 2858 | France |
| Germany | 1701 | 1742 | 2051 | 2049 | 1861 | 1905 | 2119 | 2117 | 0 | 0 | 940 | 940 | 0 | 0 | 940 | 940 | 0 | 0 | 266 | 266 | 0 | 0 | 422 | 421 | 1701 | 1742 | 3257 | 3254 | 1861 | 1905 | 3481 | 3478 | Germany |
| Italy | 1207 | 1205 | 1413 | 1410 | 1320 | 1318 | 1460 | 1457 | 0 | 0 | 648 | 647 | 0 | 0 | 648 | 647 | 0 | 0 | 183 | 183 | 0 | 0 | 291 | 290 | 1207 | 1205 | 2245 | 2240 | 1320 | 1318 | 2399 | 2394 | Italy |
| Spain | 969 | 994 | 1185 | 1197 | 1061 | 1087 | 1224 | 1236 | 0 | 0 | 543 | 549 | 0 | 0 | 543 | 549 | 0 | 0 | 154 | 155 | 0 | 0 | 244 | 246 | 969 | 994 | 1881 | 1900 | 1061 | 1087 | 2011 | 2031 | Spain |
| EU big 4 | | 5343 | 6317 | 6340 | | 5846 | 6528 | 6551 | | | 2896 | 2907 | | | 2896 | 2907 | | | 819 | 822 | | | 1299 | 1304 | 5264 | 5343 | 10033 | 10069 | 5760 | 5846 | 10723 | 10762 | **EU-4** |

# Prostate Cancer – Cross checks

## Cross check with mortality data

Nearly all prostate cancer patients die of their cancer in the metastatic castration-resistant stage,[1-3] which is considered the end stage when hormone therapies do no longer work and patients become also resistant to therapies with taxanes or can no longer be treated due to side effects.[4] ECIS specifies the mortality for prostate cancer in 2022 with 76,772 deaths.[5] This implies that roughly 77,000 patients die of end-stage mCRPC. The 80% of PSMA-positive account for 61,400 patients. If of these 5.19% were receiving a third line treatment this would translate into about 3,200 patients eligible for PSMA-radioligand therapy. – This agrees well with the upper limit derived for eligible 3rd line therapy patients starting from incidence data (1,400 – 3,000 patients).

## Cross check with 2020 ECIS prevalence data

Performing the assessment based on ECIS's prevalence data, which gives for 2020 (only 2020 data available) a number of 3,730,824 patients living with the diagnosis "prostate cancer", yields about 3,000,000 PSMA-positive patients. Shore et al. derived for the prevalence of mCRPC in the overall prostate cancer population a percentage between 1.2% and 2.1%,[6] which yields about 36,000 to 63,000 patients. This translates into about 1,900 to 3,300 patients if 5.19% of this mCRPC population receives RLT 3rd line treatment.[7] Extending the authorisation to 2nd line treatment (31.83%)[7] would add another 11,500 to 20,000 eligible patients. Applying an estimate from the US that 3.5% of the prevalent prostate cancer population has metastatic disease would yield more than 100,000 theoretically eligible PSMA-positive prostate cancer patients if the treatment could initiate already in the mHSPC stage.[8] In summary, for prostate cancer, the incidence and prevalence based estimates and the plausibility checks are in agreement.

## Comparison with Institut für Qualität und Wirtschaftlichkeit im Gesundheitswesen (IQWiG, 2023)

The Institut für Qualität und Wirtschaftlichkeit im Gesundheitswesen[9] performed a utility analysis for $^{177}$Lu-PSMA-617 in 2023 and specified for the 35,040,561 men insured in Germany in statutory health insurances an eligible patient population of 1,536 to 2,424 patients for 3rd line treatment with $^{177}$Lu-PSMA-617. This study was based on reimbursement claims to assess the stage of metastatic disease and therapies required prior to $^{177}$Lu-PSMA-617. This value is 4 to 5 times higher than the present incidence- and prevalence-based estimates for Germany in 2025. This is a strong indication that our approach provides a conservative estimate for mCRPC in 3rd line therapy.

## References


1 Svensson J, Franck Lissbrant I, Gauffin O, Hjälm-Eriksson M, Kilany S, Fagerlund K and Stattin P (2021) Time spent in hormone-sensitive and castration-resistant disease states in men with advanced prostate cancer, and its health economic impact: registry-based study in Sweden, *Scandinavian Journal of Urology*, **55**:1, 1-8, DOI: 10.1080/21681805.2020.1851762

2 Scher HI, Solo K, Valant J, Todd MB, Mehra M. Prevalence of Prostate Cancer Clinical States and Mortality in the United States: Estimates Using a Dynamic Progression Model. *PLoS One*. 2015 **13**;10(10):e0139440. doi: 10.1371/journal.pone.0139440

3 Scher HI, Heller G. Clinical states in prostate cancer: toward a dynamic model of disease progression. *Urology*. 2000; **55**(3):323-7. doi: 10.1016/s0090-4295(99)00471-9

4 Teoh JY, Ng CF. Cardiovascular risk after androgen deprivation therapy for prostate cancer: an Asian perspective. *Int Urol Nephrol*. 2016; **48**(9):1429-35. doi: 10.1007/s11255-016-1337-5

5 European Cancer Information System (2025) https://ecis.jrc.ec.europa.eu/ (Accessed 30 June 2025)

6 Shore N, Oliver L, Shui I, Gayle A, Wong OY, Kim J, Payne S, Amin S, Ghate S. Systematic Literature Review of the Epidemiology of Advanced Prostate Cancer and Associated Homologous Recombination Repair Gene Alterations. *J Urol*. 2021; **205**(4):977-986. doi: 10.1097/JU.0000000000001570

7 Leith A, Kim J, Ribbands A, Clayton E, Yang L, Ghate SR. Real-World Treatment Patterns in Metastatic Castration-Resistant Prostate Cancer Across Europe (France, Germany, Italy, Spain, and the United Kingdom) and Japan. *Adv Ther.* 2022; **39**(5):2236-2255. doi: 10.1007/s12325-022-02073-w

8 Devasia TP, Mariotto AB, Nyame YA, Etzioni R. Estimating the Number of Men Living with Metastatic Prostate Cancer in the United States. *Cancer Epidemiol Biomarkers Prev*. 2023; **32**(5):659-665. doi: 10.1158/1055-9965.EPI-22-1038

9 IQWiG (2023), Institut für Qualität und Wirtschaftlichkeit im Gesundheitswesen. (($^{177}$Lu) Lutetiumvipivotidtetraxetan (Prostatakarzinom) — Nutzenbewertung Gemäß §§35a SGB V; IQWiG35a SGB V; IQWiG--Berichte Berichte —Nr. 1538. Nr. 1538. https://www.iqwig.de/download/a23-01_177lu-lutetiumvipivotidtetraxetan_nutzenbewertung-35a-sgb-v_v1-0.pdf (accessed 13 November 2024)

# Table S16: Estimate of the evolution of the pool of PSMA+ metastatic prostate cancer patients eligible for radioligand therapies as currently authorised by EMA (2022, 2025) and for the expected extensions of future authorisations to earlier lines of treatment for the EU-27 based on ECIS incidence data.

| Parameters | lower | upper | clinical practice from literature (5) | |
|---|---|---|---|---|
| PSMA expression (1,2) | 79.3% | 87.0% | authorisation 3rd line (last option) | 5.18% |
| progression to metastatic disease (mHSPC & mCRPC) (3) | 30.0% | 40.0% | authosisation 2nd line (before chemo) | 31.83% |
| progression to mCRPC (4) | 10.0% | 20.0% | | |

| Line of treatment scenarios | lower | upper |
|---|---|---|
| 3rd line treatment only in mCRPC | 0.41% | 0.90% |
| 2nd line treatment only in mCRPC but before chemotherapy | 2.52% | 5.54% |
| 1st line tratment in all mCRPC | 7.93% | 17.40% |
| 1st line tratment in all mCRPC & mHSPC | 23.79% | 34.80% |

1) Sweat et al. (1998) Prostate-specific membrane antigen expression is greatest in prostate adenocarcinoma and lymph node metastases. Urology. 1998 Oct;52(4):637-40. doi: 10.1016/s0090-4295(98)00278-7
2) Hupe et al. (2018) Expression of Prostate-Specific Membrane Antigen (PSMA) on Biopsies Is an Independent Risk Stratifier of Prostate Cancer Patients at Time of Initial Diagnosis. Front Oncol. 2018 Dec 20;8:623. doi: 10.3389/fonc.2018.00623
3) Verry et al. (2022) Pattern of Clinical Progression Until Metastatic Castration-Resistant Prostate Cancer: An Epidemiological Study from the European Prostate Cancer Registry. Target Oncol. 2022 Jul;17(4):441-451. doi: 10.1007/s11523-022-00899-6
4) Kirby et al. (2011) Characterising the castration-resistant prostate cancer population: a systematic review. International Journal of Clinical Practice, 65: 1180-1192. https://doi.org/10.1111/j.1742-1241.2011.02799.x
5) Leith et al. (2022) Real-World Treatment Patterns in Metastatic Castration-Resistant Prostate Cancer Across Europe (France, Germany, Italy, Spain, and the United Kingdom) and Japan. Adv Ther. 2022 May;39(5):2236-2255. doi: 10.1007/s12325-022-02073-w

| ECIS Incidence, Male, Prostate, All ages, 2022 | | | ECIS projection | | | lower range | | | | upper range | | | | lower range | | | | upper range | | | | lower range | | | | upper range | | | | lower range | | | | upper range | | | |
|---|---|---|---|---|---|---|---|---|---|---|---|---|---|---|---|---|---|---|---|---|---|---|---|---|---|---|---|---|---|---|---|---|---|---|---|---|---|---|
| Country | Cases | Crude rate | Cases 2025 | Cases 2030 | Cases 2035 | 2022 | 2025 | 2030 | 2035 | 2022 | 2025 | 2030 | 2035 | 2022 | 2025 | 2030 | 2035 | 2022 | 2025 | 2030 | 2035 | 2022 | 2025 | 2030 | 2035 | 2022 | 2025 | 2030 | 2035 | 2022 | 2025 | 2030 | 2035 | 2022 | 2025 | 2030 | 2035 |
| Austria | 5,934 | 134.1 | 6,254 | 6,777 | 7,222 | 24 | 26 | 28 | 30 | 53 | 56 | 61 | 65 | 150 | 158 | 171 | 182 | 329 | 346 | 375 | 400 | 471 | 496 | 537 | 573 | 1033 | 1088 | 1179 | 1257 | 1412 | 1488 | 1612 | 1718 | 2065 | 2176 | 2358 | 2513 |
| Belgium | 10,523 | 183.5 | 11,016 | 11,872 | 12,574 | 43 | 45 | 49 | 52 | 95 | 99 | 107 | 113 | 266 | 278 | 300 | 317 | 583 | 610 | 658 | 696 | 834 | 874 | 941 | 997 | 1831 | 1917 | 2066 | 2188 | 2503 | 2621 | 2824 | 2991 | 3662 | 3834 | 4131 | 4376 |
| Bulgaria | 3,122 | 94.3 | 3,060 | 3,160 | 3,298 | 13 | 13 | 13 | 14 | 28 | 28 | 28 | 30 | 79 | 77 | 80 | 83 | 173 | 169 | 175 | 183 | 248 | 243 | 251 | 262 | 543 | 532 | 550 | 574 | 743 | 728 | 752 | 785 | 1086 | 1065 | 1100 | 1148 |
| Croatia | 3,247 | 174.4 | 3,288 | 3,411 | 3,488 | 13 | 14 | 14 | 14 | 29 | 30 | 31 | 31 | 82 | 83 | 86 | 88 | 180 | 182 | 189 | 193 | 257 | 261 | 270 | 277 | 565 | 572 | 594 | 607 | 772 | 782 | 811 | 830 | 1130 | 1144 | 1187 | 1214 |
| Cyprus | 564 | 127.9 | 595 | 654 | 703 | 2 | 2 | 3 | 3 | 5 | 5 | 6 | 6 | 14 | 15 | 17 | 18 | 31 | 33 | 36 | 39 | 45 | 47 | 52 | 56 | 98 | 104 | 114 | 122 | 134 | 142 | 156 | 167 | 196 | 207 | 228 | 245 |
| Czechia | 7,956 | 153.5 | 8,125 | 8,513 | 8,958 | 33 | 33 | 35 | 37 | 72 | 73 | 77 | 81 | 201 | 205 | 215 | 226 | 441 | 450 | 471 | 496 | 631 | 644 | 675 | 710 | 1384 | 1414 | 1481 | 1559 | 1893 | 1933 | 2025 | 2131 | 2769 | 2828 | 2963 | 3117 |
| Denmark | 5,250 | 179.6 | 5,481 | 5,802 | 6,038 | 22 | 23 | 24 | 25 | 47 | 49 | 52 | 54 | 133 | 138 | 146 | 152 | 291 | 304 | 321 | 334 | 416 | 435 | 460 | 479 | 914 | 954 | 1010 | 1051 | 1249 | 1304 | 1380 | 1436 | 1827 | 1907 | 2019 | 2101 |
| EU-27 | 330,492 | 151.4 | 343,426 | 368,578 | 390,858 | 1358 | 1411 | 1514 | 1606 | 2979 | 3095 | 3322 | 3523 | 8342 | 8668 | 9303 | 9866 | 18304 | 19020 | 20413 | 21647 | 26208 | 27234 | 29228 | 30995 | 57506 | 59756 | 64133 | 68009 | 78624 | 81701 | 87685 | 92985 | 115011 | 119512 | 128265 | 136019 |
| Estonia | 1,174 | 185.3 | 1,223 | 1,309 | 1,399 | 5 | 5 | 5 | 6 | 11 | 11 | 12 | 13 | 30 | 31 | 33 | 35 | 65 | 68 | 72 | 77 | 93 | 97 | 104 | 111 | 204 | 213 | 228 | 243 | 279 | 291 | 311 | 333 | 409 | 426 | 456 | 487 |
| Finland | 5,930 | 216.2 | 6,213 | 6,536 | 6,729 | 24 | 26 | 27 | 28 | 53 | 56 | 59 | 61 | 150 | 157 | 165 | 170 | 328 | 344 | 362 | 373 | 470 | 493 | 518 | 534 | 1032 | 1081 | 1137 | 1171 | 1411 | 1478 | 1555 | 1601 | 2064 | 2162 | 2275 | 2342 |
| France | 57,357 | 174.7 | 59,958 | 63,607 | 65,961 | 236 | 246 | 261 | 271 | 517 | 540 | 573 | 595 | 1448 | 1513 | 1606 | 1665 | 3177 | 3321 | 3523 | 3653 | 4548 | 4755 | 5044 | 5231 | 9980 | 10433 | 11068 | 11477 | 13645 | 14264 | 15132 | 15692 | 19960 | 20865 | 22135 | 22954 |
| Germany | 65,269 | 158.9 | 67,237 | 72,273 | 77,919 | 268 | 276 | 297 | 320 | 588 | 606 | 651 | 702 | 1647 | 1697 | 1824 | 1967 | 3615 | 3724 | 4003 | 4315 | 5176 | 5332 | 5731 | 6179 | 11357 | 11699 | 12576 | 13558 | 15527 | 15996 | 17194 | 18537 | 22714 | 23398 | 25151 | 27116 |
| Greece | 7,036 | 137.6 | 7,104 | 7,566 | 8,030 | 29 | 29 | 31 | 33 | 63 | 64 | 68 | 72 | 178 | 179 | 191 | 203 | 390 | 393 | 419 | 445 | 558 | 563 | 600 | 637 | 1224 | 1236 | 1316 | 1397 | 1674 | 1690 | 1800 | 1910 | 2449 | 2472 | 2633 | 2794 |
| Hungary | 6,660 | 143.4 | 6,623 | 6,797 | 7,188 | 27 | 27 | 28 | 30 | 60 | 60 | 61 | 65 | 168 | 167 | 172 | 181 | 369 | 367 | 376 | 398 | 528 | 525 | 539 | 570 | 1159 | 1152 | 1183 | 1251 | 1584 | 1576 | 1617 | 1710 | 2318 | 2305 | 2365 | 2501 |
| Ireland | 4,216 | 168.4 | 4,528 | 5,039 | 5,555 | 17 | 19 | 21 | 23 | 38 | 41 | 45 | 50 | 106 | 114 | 127 | 140 | 233 | 251 | 279 | 308 | 334 | 359 | 400 | 441 | 734 | 788 | 877 | 967 | 1003 | 1077 | 1199 | 1322 | 1467 | 1576 | 1754 | 1933 |
| Italy | 38,180 | 132.5 | 39,357 | 42,575 | 45,593 | 157 | 162 | 175 | 187 | 344 | 355 | 384 | 411 | 964 | 993 | 1075 | 1151 | 2115 | 2180 | 2358 | 2525 | 3028 | 3121 | 3376 | 3616 | 6643 | 6848 | 7408 | 7933 | 9083 | 9363 | 10129 | 10847 | 13287 | 13696 | 14816 | 15866 |
| Latvia | 1,662 | 191.2 | 1,685 | 1,745 | 1,804 | 7 | 7 | 7 | 7 | 15 | 15 | 16 | 16 | 42 | 43 | 44 | 46 | 92 | 93 | 97 | 100 | 132 | 134 | 138 | 143 | 289 | 293 | 304 | 314 | 395 | 401 | 415 | 429 | 578 | 586 | 607 | 628 |
| Lithuania | 3,208 | 245.5 | 3,416 | 3,558 | 3,677 | 13 | 14 | 15 | 15 | 29 | 31 | 32 | 33 | 81 | 86 | 90 | 93 | 178 | 189 | 197 | 204 | 254 | 271 | 282 | 292 | 558 | 594 | 619 | 640 | 763 | 813 | 846 | 875 | 1116 | 1189 | 1238 | 1280 |
| Luxembourg | 443 | 136.3 | 481 | 560 | 644 | 2 | 2 | 2 | 3 | 4 | 4 | 5 | 6 | 11 | 12 | 14 | 16 | 25 | 27 | 31 | 36 | 35 | 38 | 44 | 51 | 77 | 84 | 97 | 112 | 105 | 114 | 133 | 153 | 154 | 167 | 195 | 224 |
| Malta | 284 | 105.2 | 299 | 332 | 367 | 1 | 1 | 1 | 2 | 3 | 3 | 3 | 3 | 7 | 8 | 8 | 9 | 16 | 17 | 18 | 20 | 23 | 24 | 26 | 29 | 49 | 52 | 58 | 64 | 68 | 71 | 79 | 87 | 99 | 104 | 116 | 128 |
| Netherlands | 11,956 | 136.7 | 12,593 | 13,513 | 14,100 | 49 | 52 | 56 | 58 | 108 | 114 | 122 | 127 | 302 | 318 | 341 | 356 | 662 | 697 | 748 | 781 | 948 | 999 | 1072 | 1118 | 2080 | 2191 | 2351 | 2453 | 2844 | 2996 | 3215 | 3354 | 4161 | 4382 | 4703 | 4907 |
| Poland | 22,480 | 123.4 | 23,261 | 24,812 | 26,002 | 92 | 96 | 102 | 107 | 203 | 210 | 224 | 234 | 567 | 587 | 626 | 656 | 1245 | 1288 | 1374 | 1440 | 1783 | 1845 | 1968 | 2062 | 3912 | 4047 | 4317 | 4524 | 5348 | 5534 | 5903 | 6186 | 7823 | 8095 | 8635 | 9049 |
| Portugal | 7,529 | 152.9 | 8,292 | 8,869 | 9,366 | 31 | 34 | 36 | 38 | 68 | 75 | 80 | 84 | 190 | 209 | 224 | 236 | 417 | 459 | 491 | 519 | 597 | 658 | 703 | 743 | 1310 | 1443 | 1543 | 1630 | 1791 | 1973 | 2110 | 2228 | 2620 | 2886 | 3086 | 3259 |
| Romania | 10,442 | 113 | 10,490 | 10,964 | 11,625 | 43 | 43 | 45 | 48 | 94 | 95 | 99 | 105 | 264 | 265 | 277 | 293 | 578 | 581 | 607 | 644 | 828 | 832 | 869 | 922 | 1817 | 1825 | 1908 | 2023 | 2484 | 2496 | 2608 | 2766 | 3634 | 3651 | 3815 | 4046 |
| Slovakia | 3,606 | 135.7 | 3,794 | 4,119 | 4,414 | 15 | 16 | 17 | 18 | 33 | 34 | 37 | 40 | 91 | 96 | 104 | 111 | 200 | 210 | 228 | 244 | 286 | 301 | 327 | 350 | 627 | 660 | 717 | 768 | 858 | 903 | 980 | 1050 | 1255 | 1320 | 1433 | 1536 |
| Slovenia | 1,765 | 166.9 | 1,861 | 2,007 | 2,128 | 7 | 8 | 8 | 9 | 16 | 17 | 18 | 19 | 45 | 47 | 51 | 54 | 98 | 103 | 111 | 118 | 140 | 148 | 159 | 169 | 307 | 324 | 349 | 370 | 420 | 443 | 477 | 506 | 614 | 648 | 698 | 741 |
| Spain | 32,967 | 141.9 | 35,187 | 39,198 | 42,998 | 135 | 145 | 161 | 177 | 297 | 317 | 353 | 388 | 832 | 888 | 989 | 1085 | 1826 | 1949 | 2171 | 2381 | 2614 | 2790 | 3108 | 3410 | 5736 | 6123 | 6820 | 7482 | 7843 | 8371 | 9325 | 10229 | 11473 | 12245 | 13641 | 14963 |
| Sweden | 11,732 | 223 | 12,112 | 12,701 | 13,239 | 48 | 50 | 52 | 54 | 106 | 109 | 114 | 119 | 296 | 306 | 321 | 334 | 650 | 671 | 703 | 733 | 930 | 960 | 1007 | 1050 | 2041 | 2107 | 2210 | 2304 | 2791 | 2881 | 3022 | 3150 | 4083 | 4215 | 4420 | 4607 |

Source: ECIS - European Cancer Information System
From https://ecis.jrc.ec.europa.eu/
(C) European union

| ECIS Incidence, Male, Prostate, All ages, 2022 | | | ECIS projection | | | lower range | | | | upper range | | | | lower range | | | | upper range | | | | lower range | | | | upper range | | | | lower range | | | | upper range | | | |
|---|---|---|---|---|---|---|---|---|---|---|---|---|---|---|---|---|---|---|---|---|---|---|---|---|---|---|---|---|---|---|---|---|---|---|---|---|---|---|
| Country | Cases | Crude rate | Cases 2025 | Cases 2030 | Cases 2035 | 2022 | 2025 | 2030 | 2035 | 2022 | 2025 | 2030 | 2035 | 2022 | 2025 | 2030 | 2035 | 2022 | 2025 | 2030 | 2035 | 2022 | 2025 | 2030 | 2035 | 2022 | 2025 | 2030 | 2035 | 2022 | 2025 | 2030 | 2035 | 2022 | 2025 | 2030 | 2035 |
| EU-27 | 330,492 | 151.4 | 343,426 | 368,578 | 390,858 | 1358 | 1411 | 1514 | 1606 | 2979 | 3095 | 3322 | 3523 | 8342 | 8668 | 9303 | 9866 | 18304 | 19020 | 20413 | 21647 | 26208 | 27234 | 29228 | 30995 | 57506 | 59756 | 64133 | 68009 | 78624 | 81701 | 87685 | 92985 | 115011 | 119512 | 128265 | 136019 |
| France | 57,357 | 174.7 | 59,958 | 63,607 | 65,961 | 236 | 246 | 261 | 271 | 517 | 540 | 573 | 595 | 1448 | 1513 | 1606 | 1665 | 3177 | 3321 | 3523 | 3653 | 4548 | 4755 | 5044 | 5231 | 9980 | 10433 | 11068 | 11477 | 13645 | 14264 | 15132 | 15692 | 19960 | 20865 | 22135 | 22954 |
| Germany | 65,269 | 158.9 | 67,237 | 72,273 | 77,919 | 268 | 276 | 297 | 320 | 588 | 606 | 651 | 702 | 1647 | 1697 | 1824 | 1967 | 3615 | 3724 | 4003 | 4315 | 5176 | 5332 | 5731 | 6179 | 11357 | 11699 | 12576 | 13558 | 15527 | 15996 | 17194 | 18537 | 22714 | 23398 | 25151 | 27116 |
| Italy | 38,180 | 132.5 | 39,357 | 42,575 | 45,593 | 157 | 162 | 175 | 187 | 344 | 355 | 384 | 411 | 964 | 993 | 1075 | 1151 | 2115 | 2180 | 2358 | 2525 | 3028 | 3121 | 3376 | 3616 | 6643 | 6848 | 7408 | 7933 | 9083 | 9363 | 10129 | 10847 | 13287 | 13696 | 14816 | 15866 |
| Spain | 32,967 | 141.9 | 35,187 | 39,198 | 42,998 | 135 | 145 | 161 | 177 | 297 | 317 | 353 | 388 | 832 | 888 | 989 | 1085 | 1826 | 1949 | 2171 | 2381 | 2614 | 2790 | 3108 | 3410 | 5736 | 6123 | 6820 | 7482 | 7843 | 8371 | 9325 | 10229 | 11473 | 12245 | 13641 | 14963 |
| EU big 4 | | | | | | 796 | 829 | 894 | 955 | 1747 | 1818 | 1961.8 | 2095.3 | | 5092 | | | | 11173 | | | | | 17260 | | | | 37872 | | | | | 55305 | | | | 80900 |

## Partitioning ECIS incidence numbers for haematological cancer sub-indications

In Table S17a the disease sub-types under clinical investigation are assigned to the ECIS main categories leukaemia, Non-Hodgkin's Lymphoma (NHL) and multiple myeloma (MM) based on matching ICD-10 codes.[1]

**Table S17a: Distribution of ECIS incidence data for leukaemia, non-Hodgkin's lymphoma and multiple myeloma data on the disease subtypes as derived from the data base of the** UK's Haematological Malignancy Research Network (HMRN).[2]

| ECIS cancer name | ICD-10 code (2010 version) | Indications investigated in clinical trials | | ICD-10 code | Percentages to be assigned to specific disease subtypes | |
|---|---|---|---|---|---|---|
| Leukaemia | C91-95 | Acute myeloid leukaemia | AML | C92 | 27.85% | percentages of leuklaemia data |
| | | Chronic lymphocytik leukaemia | CLL | C91.1 | 36.36-40.91% | |
| | | Small lymphocytik lymphoma | SLL | C83.0 | 4.55-9.10% | |
| Non-Hodgkin lymphoma (NHL) | C82-86+C88, B21.1-31 | Follicular lymphoma | FL | C82 | 18.19% | percentages of NHL data |
| | | Large B-cell lymphomas | DLBCL | C83.3 | 41.93% | |
| | | Mantle cell lymphpma | MCL | C83.5 | 4.53% | |
| | | Marginal zone lymphoma | MZL | C85.1 | 20.75% | |
| | | Lymphoplasmacytic lymphoma | LPL | C83.0 | n.a. | |
| | | Waldenström's Macroglobulinemia | WM | C88.0 | n.a. | |
| Multiple myeloma | C90 | Multiple Myeloma | MM | C90 | 93.50% | percentage of MM data |

1 n.a. – data not available

2 Based on its ICD-10 code small lymphocytic lymphoma (SLL) belongs to the Non-Hodgkin's Lymphoma, but it is considered a special variant of chronic lymphatic leukaemia (CLL). 10 to 20% of the CLL cases qualify as SLL. Therefore, the calculation is based on the leukaemia incidences also for SLL.

3 By their ICD-10 codes lymphoplasmacytic lymphoma (LPL) and Waldenström's Macroglobulinemia (WM) belong to Non-Hodgkin's Lymphoma, but they are rare and not listed in the UK's Haematological Malignancy Research Network (HMRN) database. The number of patients was derived from published incidence data.

Since ECIS incidence data are available only globally for these main categories the contribution of the subtypes of interest for the clinical trials to each of the three main categories was determined in proportion to incidence rates for about 30 haematological diseases (subtypes) retrieved from the data base of the Haematological Malignancy Research Network (HMRN) in the UK.[1,2] The HMRN incidence rates are compiled in Table S17b and assigned to the three ECIS main categories based on matching ICD-10 codes.

The fraction of the ECIS incidence data that correspond to the relevant haematological cancer subtypes was then determined as a fraction of the incidence rate of each subtype in proportion to the sum of all HMRN incidence rates assigned to the corresponding ECIS main category as presented in Table S17b.

The partitioning based on the ICD-10 codes and the incidence rates provided by the HMRN compiled in Table 17b shows that 73.3% of the leukaemia cases provided by ECIS need to be considered. These are sub-distributed between AML and CLL as 38% and 62%, respectively. The subtypes of Non-Hodgkin's Lymphoma (NHL) mentioned in the clinical trials account for 85.4% of all NHL cases given by ECIS. Specifically, 49.1% of the total ECIS incidence number for NHL is attributed to DLBCL, followed by MZL with 20.7%, FL with 18.2% and MCL with 4.5%. The estimate for multiple myeloma will be based on 93.5% of all MM cases reported in ECIS.

The HMRN does not provide data for WM, SLL and LPL. In these cases, incidence rates and information from literature were used. SLL is considered a subtype of CLL without leukemic manifestation that accounts for 10-20% of the CLL/SLL cases (see also https://seer.cancer.gov/statfacts/html/cllsll.html ).[3,4] Thus, they are both considered as sub-types of the same disease. Therefore, derived from ECIS, following the partitioning based on incidence data provided by the HMRN, the numbers for CLL are attributed to 80-90% to CLL, in the narrower interpretation of the disease, and 10-20% are assigned to SLL. Similarly, Waldenström's Macroglobulinemia (WM) is considered the most common sub-type of lymphoplasmacytic lymphoma (LPL), which are both rare types of Non-Hodgkin's Lymphoma (NHL).[3,5] The HMRN provides incidence data neither for WM nor for LPL. In literature the incidence for WM is given as 0.36/100,000 to 0.55/100,000 in the US and Europe, which agrees with 0.36/100,000 given for WM in the US (in the years 2000 to 2019) and 0.27/100,000 for other LPL in the US in the same period.[5,6]

**Table S17b: Linking the incidence rates compiled by the Haematological Malignancies Research Network to the sub-types of leukaemia, Non-Hodgkins Lymphoma and multiple myeloma, which need to be considered for the present work.** - *https://hmrn.org/statistics/incidence* (accessed 24 July 2024)

| Incidence rates source: Haematological Malignancy Research Network | | | | Partitioning of ECIS categories in sub-types in clinical trials | | | | | | | | | | | |
|---|---|---|---|---|---|---|---|---|---|---|---|---|---|---|---|
| | | | | Leukaemia | | | | Non-Hodgkins Lymphoma | | | | Multiple Myeloma | | | |
| | | | | incidence rates | | | | incidence rates | | | | incidence rates | | | |
| **Disease tabulated**<br>**source: Haematological Malignancy Research Network accessed 04 April 2024** | **Abbreviation** | **ICD-10 code (2010 version)** | **incidence rate per 100.000** | all leukaemia | relevant for trials | % of Leukaemias | % of relevant sub-types | all Non-Hodgkins Lymphoma | relevant for trials | % of Non-Hodgkins LymphomaHL | % of relevant sub-types | multiple myeloma | relevant for trials | % of multiple myeloma | % of relevant sub-types |
| **Myeloproliferative neoplasms** | | | 8.000 | | | | | | | | | | | | |
| Chronic myeloid leukaemia | | C92.1 | 1.100 | 1.100 | | | | | | | | | | | |
| Myelofibrosis | | D47.1 | 0.600 | | | | | | | | | | | | |
| Polycythaemia vera | | D45 | 1.800 | | | | | | | | | | | | |
| Essential thrombocythaemia | | D47.3 | 4.200 | | | | | | | | | | | | |
| Myeloproliferative neoplasm, unclassifiable | | D47.1 | 0.400 | | | | | | | | | | | | |
| Myelodysplastic / Myeloproliferative neoplasms | | | 1.300 | | | | | | | | | | | | |
| Chronic myelomonocytic leukaemia | | C93.1 | 1.000 | 1.000 | | | | | | | | | | | |
| Atypical chronic myeloid leukaemia | | C92.2 | 0.100 | 0.100 | | | | | | | | | | | |
| Myelodysplastic / myeloproliferative neoplasm unclassified | | D47.1 | 0.200 | | | | | | | | | | | | |
| MDS/MPN with ring sideroblasts and thrombocytosis | | D47.03 | 0.100 | | | | | | | | | | | | |
| Myelodysplastic syndromes | | D47 | 3.300 | | | | | | | | | | | | |
| Acute myeloid leukaemias | **AML** | | 4.400 | | | | | | | | | | | | |
| Acute myeloid leukaemia | | C92 | 4.200 | 4.200 | 4.200 | 26.1% | 38% | | | | | | | | |
| Acute promyelocytic leukaemia | | C92.4 | 0.300 | 0.300 | 0.300 | 1.9% | | | | | | | | | |
| Acute lymphoblastic leukaemia | | | 1.200 | | | | | | | | | | | | |
| B-lymphoblastic leukaemia | | C91.4 | 1.000 | 1.000 | | | | | | | | | | | |
| T-lymphoblastic leukaemia | | C91.7 | 0.200 | 0.200 | | | | | | | | | | | |
| Mature B-cell neoplasms | | | 34.900 | | | | | | | | | | | | |
| Chronic lymphocytic leukaemia | **CLL** | C91.1 | 7.300 | 7.300 | 7.300 | 45.3% | 62% | | | | | | | | |
| Hairy cell leukaemia | | C91.4 | 0.400 | 0.400 | | | | | | | | | | | |
| Lymphoproliferative disorders, NOS | | D47.9 | 2.400 | | | | | | | | | | | | |
| Marginal zone lymphoma | **MZL** | C85.1 | 4.100 | | | | | 4.100 | 4.100 | 20.7% | 24.3% | | | | |
| Plasmacytoma | | C90 | 0.500 | | | | | | | | | 0.500 | | | |
| Myeloma | **MM** | C90 | 7.200 | | | | | | | | | 7.200 | 7.200 | 93.5% | 100.0% |
| Follicular lymphoma | **FL** | C82 | 3.600 | | | | | 3.600 | 3.600 | 18.2% | 21.3% | | | | |
| Mantle cell lymphoma | **MCL** | C83.5 | 0.900 | | | | | 0.900 | 0.900 | 4.5% | 5.3% | | | | |
| Large B-cell lymphomas | **DLBCL** | C83.3 | 8.300 | | | | | 8.300 | 8.300 | 41.9% | 49.1% | | | | |
| Burkitt lymphoma | | C83.7 | 0.400 | | | | | 0.400 | | | | | | | |
| Mature T- and NK-cell neoplasms | | C84 | 1.500 | | | | | 1.500 | | | | | | | |
| Peripheral T-cell lymphomas | | C84.4 | 0.800 | | | | | 0.800 | | | | | | | |
| Cutaneous T-cell lymphomas | | C84.0 | 0.200 | | | | | 0.200 | | | | | | | |
| T-cell prolymphocytic leukaemia | | C91.6 | 0.100 | 0.100 | | | | | | | | | | | |
| T-cell large granular lymphocytic leukaemia | | C91.5 | 0.400 | 0.400 | | | | | | | | | | | |
| Hodgkin lymphoma | | | 2.800 | | | | | | | | | | | | |
| Classical Hodgkin lymphoma | | C81 | 2.500 | | | | | | | | | | | | |
| Nodular lymphocyte predominant Hodgkin lymphoma | | C81.4 | 0.300 | | | | | | | | | | | | |
| | | | Sum | 16.100 | 11.800 | 73.3% | 100% | 19.800 | 16.900 | 85.4% | 100.0% | 7.700 | 7.200 | 93.5% | 100.0% |

After having determined the number of new cancer cases per year for the relevant indications, the number of eligible patients can be derived. This needs to take into account the prevalence of the targets for the radioligand therapies, the medical indications for which the therapy is currently authorised or subject to clinical trials and the number of patients at the appropriate stage of disease. The workflow of the data evaluation is depicted in Figure S6.

The more uncertain part is predicting which radioligand therapies currently in clinical trials may take the next steps towards marketing authorisation and further to reimbursement and integration into clinical routine. Since for many indications (CLL, SLL, DLBCL, MZL, FL, MCL) the clinical phase 2 trials were labelled as closed, no published results could be identified and there was no evidence for any type of phase 2 or 3 follow-up clinical trial, these indications were no longer considered for the present patient estimate. Therefore, **the present work was restricted to AML, MM and LPL/WM.**

**Figure S6: Partitioning scheme of haematological ECIS incidence data into disease sub-types. - The derivation of the numbers of patients for specific haematological indications (cancer types and sub-types) is based on the data given by ECIS for three major cancer categories. The partitioning is based on incidence-rate data provided by the UK's Haematological Malignancies Research Network (HMRN) and for sub-types not explicitly listed in the HMRN, on ECIS population data applying literature values for incidence rates. Estimating the number of eligible patients for specific haematological indications requires additional information from literature on the prevalence of target expression and the stage of disease (in which the treatment is investigated or is authorised) and other conditions, which affect whether a patients can be treated or not.**

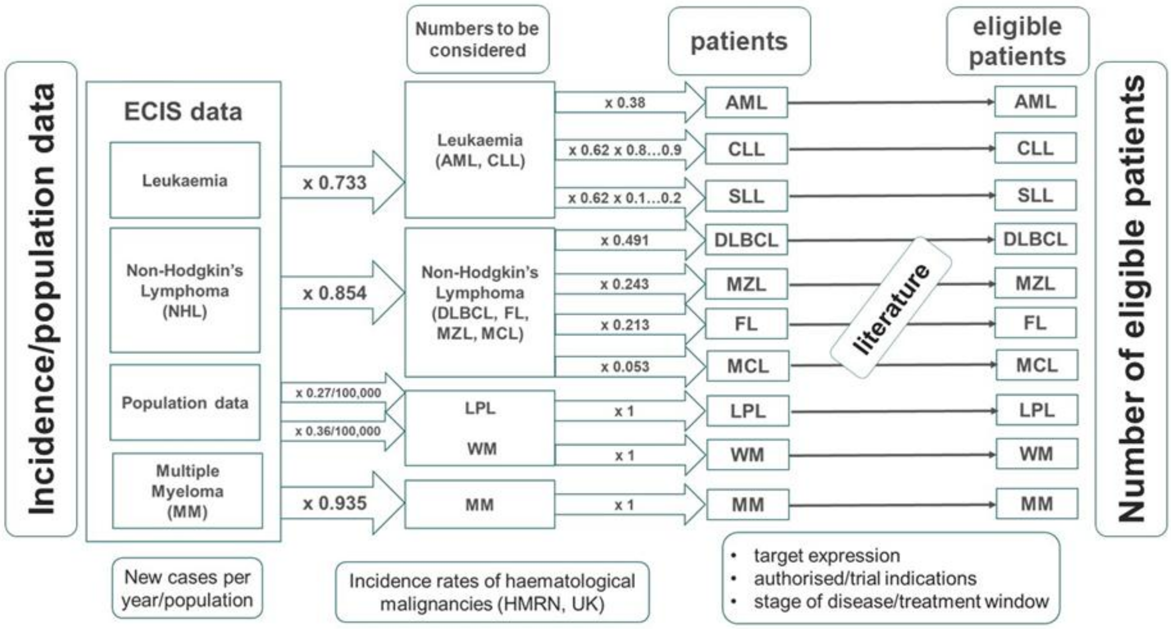



1. https://ecis.jrc.ec.europa.eu/ (Accessed 30 June 2024)
2. Haematological Malignancies Research Network https://hmrn.org/statistics/incidence (accessed 24 July 2024)
3. Alaggio R, Amador C, Anagnostopoulos I, et al. The 5th edition of the World Health Organization Classification of Haematolymphoid Tumours: Lymphoid Neoplasms. *Leukemia.* 2022; **36**(7):1720-1748. doi: 10.1038/s41375-022-01620-2. Erratum in: *Leukemia.* 2023; **37**(9):1944-1951. doi: 10.1038/s41375-023-01962-5
4. Andres M, Feller A, Arndt V, The Nicer Working Group. Trends of incidence, mortality and survival for chronic lymphocytic leukaemia / small lymphocytic lymphoma in Switzerland between 1997 and 2016: a population-based study. *Swiss Med Wkly.* 2021; **151**:w20463. doi: 10.4414/smw.2021.20463
5. McMaster ML. The epidemiology of Waldenström macroglobulinemia. *Semin Hematol*. 2023; **60**(2):65-72. doi: 10.1053/j.seminhematol.2023.03.008
6. García-Sanz R, Tedeschi A. The Management of Relapsed or Refractory Waldenström's Macroglobulinemia. *Hematol Oncol Clin North Am.* 2023; **37**(4):727-749. doi: 10.1016/j.hoc.2023.04.006

# Table S17c: Distribution of ECIS data for Leukaemia, Myeloma and Non-Hodgkin Lymphoma on disease subtypes relevant or the present study

**Source:** incidence data and long-term incidence projections of European Cancer Information System (ECIS) https://ecis.jrc.ec.europa.eu/index.php (**see below**) and incidence statistics of the Haematological Malignancies Research Network in the UK ( https://hmrn.org/statistics/incidence ) (**see sheet "(1) HMRN - downloaded data"**)

**Comment 1:** ECIS provides only global incidence data for Leukaemia, Multiple myeloma and Non-Hodgkin lymphoma. Therefore the total numbers need to be decomposed into the number of patients with subtypes of those malignancies for which therapies are in clinical trials. In order to do this decomposition of the ECIS data in subtypes, the incidence rate data of the HMRN were used (**see worksheet "HMRN-ECIS connection"**).

**Comment 2:** In order to attribute the subtypes of malignancies to the proper ECIS category for each malignancy reported by the HMRN the proper ICD-10 code was researched since the atribution of cancers to the ECIS database is based on ICD-10 codes (**see worksheet "(2) ECIS cancer classification"**).

**Comment 3:** The HMRN incidence rates of all subtypes were summed up which belonged to the ICD-10 codes used by ECIS for leukaemia, multiple myeloma and Non-Hodgkin lymphoma, respectively. Then the HMRN incidence rates given by the HMRN for the malignancies which need to be considered for the present work were summed up. The ratio of both values gives the fraction of the ECIS incidence (number of new patients per year) which are relevant for the present estimate (**for the summary see worksheet "(5) ECIS Fractions", for the calculations see "(4) HMRN-ECIS connection"**).

**Comment 4:** The same ratio of the HMRN incidence rates was then applied to derive the number of patients belonging to the relevant subtypes of leukaemia, myeloma and Non-Hodgkin lymphoma (see below), 73.3%, 93.5% and 84.5%, respectively.

**Comment 5:** HMRN does not provide data for Waldenstrom macroglobulinemia (WM) and lymphoplasmacytic lymphoma (LPL). Both malignancies belong to the ICD-10 code C88 and are covered by ECIS. Both together are rare malignancies with incidence rates (in the US) of 0.27/100.000 (LPL) and 0.36/100.000 (WM) (ML McMaster 2023 (https://doi.org/10.1053/j.seminhematol.2023.03.008). With the present method, the error of neglecting this malignancy is about 3% for the total number of lymphoma patients.

**Comment 6:** Since SLL is considered a subtype of CLL which accounts for 10%-20% if the CLL cases, which are in lireature both considered Leukaemia while the ICD-10 coding for SLL (C83) classifies it as NHL in ECIS. Assigning it to Leukaemia this may lead to to an overestimate of MZL, MCL, FL and DLBCL cases by 3.5%-7.5%. In view of much larger uncertainties caused by other effects this has not be considered further.

| | ECIS data | | | | | Fraction AML & CLL of all leukemia | | | 0.733 |
|---|---|---|---|---|---|---|---|---|---|
| | ALL Leukemia | | | | | AML and CLL only | | | |
| Country | Cases 2022 | Crude rate | Cases 2025 | Cases 2030 | Cases 2035 | 2022 | 2025 | 2030 | 2035 |
| Austria | 1,218 | 13.6 | 1,272 | 1,355 | 1,437 | 893 | 932 | 993 | 1053 |
| Belgium | 2,222 | 19.1 | 2,301 | 2,430 | 2,562 | 1629 | 1687 | 1781 | 1878 |
| Bulgaria | 639 | 9.3 | 634 | 638 | 642 | 468 | 465 | 468 | 471 |
| Croatia | 690 | 17.9 | 685 | 703 | 730 | 506 | 502 | 515 | 535 |
| Cyprus | 182 | 20.1 | 192 | 214 | 236 | 133 | 141 | 157 | 173 |
| Czechia | 1,663 | 15.8 | 1,726 | 1,829 | 1,919 | 1219 | 1265 | 1341 | 1407 |
| Denmark | 1,166 | 19.9 | 1,223 | 1,306 | 1,365 | 855 | 896 | 957 | 1001 |
| EU-27 | 73,589 | 16.5 | 75,821 | 80,240 | 84,873 | 53941 | 55577 | 58816 | 62212 |
| Estonia | 232 | 17.4 | 239 | 250 | 261 | 170 | 175 | 183 | 191 |
| Finland | 816 | 14.7 | 851 | 900 | 943 | 598 | 624 | 660 | 691 |
| France | 13,525 | 19.9 | 14,066 | 15,025 | 15,979 | 9914 | 10310 | 11013 | 11713 |
| Germany | 15,108 | 18.2 | 15,557 | 16,124 | 16,737 | 11074 | 11403 | 11819 | 12268 |
| Greece | 1,938 | 18.5 | 1,923 | 1,994 | 2,087 | 1421 | 1410 | 1462 | 1530 |
| Hungary | 1,387 | 14.3 | 1,395 | 1,437 | 1,483 | 1017 | 1023 | 1053 | 1087 |
| Ireland | 568 | 11.2 | 609 | 674 | 746 | 416 | 446 | 494 | 547 |
| Italy | 10,799 | 18.3 | 10,944 | 11,546 | 12,204 | 7916 | 8022 | 8463 | 8946 |
| Latvia | 292 | 15.6 | 294 | 293 | 294 | 214 | 216 | 215 | 216 |
| Lithuania | 473 | 16.9 | 484 | 501 | 519 | 347 | 355 | 367 | 380 |
| Luxembourg | 78 | 12.1 | 83 | 94 | 109 | 57 | 61 | 69 | 80 |
| Malta | 51 | 9.8 | 54 | 60 | 67 | 37 | 40 | 44 | 49 |
| Netherlands | 3,136 | 17.8 | 3,284 | 3,503 | 3,682 | 2299 | 2407 | 2568 | 2699 |
| Poland | 5,152 | 13.7 | 5,327 | 5,733 | 6,156 | 3776 | 3905 | 4202 | 4512 |
| Portugal | 1,565 | 15.1 | 1,683 | 1,781 | 1,881 | 1147 | 1234 | 1305 | 1379 |
| Romania | 1,975 | 10.4 | 1,994 | 2,039 | 2,094 | 1448 | 1462 | 1495 | 1535 |
| Slovakia | 699 | 12.9 | 726 | 784 | 837 | 512 | 532 | 575 | 614 |
| Slovenia | 403 | 19.1 | 425 | 463 | 501 | 295 | 312 | 339 | 367 |
| Spain | 5,976 | 12.6 | 6,218 | 6,780 | 7,402 | 4380 | 4558 | 4970 | 5426 |
| Sweden | 1,636 | 15.7 | 1,695 | 1,802 | 1,886 | 1199 | 1242 | 1321 | 1382 |
| | Source: ECIS - European Cancer Information System | | | | | | | | |
| | From https://ecis.jrc.ec.europa.eu/ | | | | | | | | |
| | (C) European union | | | | | | | | |

| | ECIS data | | | | | Fraction of multiple myeloma | | | 0.935 |
|---|---|---|---|---|---|---|---|---|---|
| | ALL Myeloma | | | | | Multiple myeloma only | | | |
| Country | Cases 2022 | Crude rate | Cases 2025 | Cases 2030 | Cases 2035 | 2022 | 2025 | 2030 | 2035 |
| Austria | 538 | 6 | 563 | 609 | 654 | 503 | 526 | 569 | 611 |
| Belgium | 1,013 | 8.7 | 1052 | 1,125 | 1,198 | 947 | 984 | 1052 | 1120 |
| Bulgaria | 208 | 3 | 209 | 209 | 212 | 194 | 195 | 195 | 198 |
| Croatia | 400 | 10.4 | 400 | 415 | 423 | 374 | 374 | 388 | 396 |
| Cyprus | 78 | 8.6 | 81 | 91 | 103 | 73 | 76 | 85 | 96 |
| Czechia | 624 | 5.9 | 647 | 671 | 695 | 583 | 605 | 627 | 650 |
| Denmark | 661 | 11.3 | 691 | 733 | 766 | 618 | 646 | 685 | 716 |
| EU-27 | 35,333 | 7.9 | 36556 | 38,938 | 41,264 | 33036 | 34180 | 36407 | 38582 |
| Estonia | 112 | 8.4 | 113 | 122 | 126 | 105 | 106 | 114 | 118 |
| Finland | 466 | 8.4 | 492 | 521 | 545 | 436 | 460 | 487 | 510 |
| France | 5,425 | 8 | 5695 | 6,112 | 6,441 | 5072 | 5325 | 5715 | 6022 |
| Germany | 6,932 | 8.3 | 7108 | 7,408 | 7,764 | 6481 | 6646 | 6926 | 7259 |
| Greece | 763 | 7.3 | 766 | 805 | 844 | 713 | 716 | 753 | 789 |
| Hungary | 404 | 4.2 | 407 | 416 | 433 | 378 | 381 | 389 | 405 |
| Ireland | 438 | 8.7 | 476 | 538 | 606 | 410 | 445 | 503 | 567 |
| Italy | 6,298 | 10.7 | 6443 | 6,873 | 7,294 | 5889 | 6024 | 6426 | 6820 |
| Latvia | 119 | 6.3 | 120 | 122 | 122 | 111 | 112 | 114 | 114 |
| Lithuania | 179 | 6.4 | 186 | 191 | 199 | 167 | 174 | 179 | 186 |
| Luxembourg | 38 | 5.9 | 38 | 47 | 53 | 36 | 36 | 44 | 50 |
| Malta | 32 | 6.1 | 33 | 39 | 45 | 30 | 31 | 36 | 42 |
| Netherlands | 1,607 | 9.1 | 1691 | 1,817 | 1,916 | 1503 | 1581 | 1699 | 1791 |
| Poland | 2,433 | 6.5 | 2507 | 2,672 | 2,844 | 2275 | 2344 | 2498 | 2659 |
| Portugal | 994 | 9.6 | 1080 | 1,149 | 1,217 | 929 | 1010 | 1074 | 1138 |
| Romania | 725 | 3.8 | 738 | 757 | 771 | 678 | 690 | 708 | 721 |
| Slovakia | 337 | 6.2 | 351 | 383 | 415 | 315 | 328 | 358 | 388 |
| Slovenia | 171 | 8.1 | 180 | 193 | 210 | 160 | 168 | 180 | 196 |
| Spain | 3,434 | 7.2 | 3616 | 4,015 | 4,437 | 3211 | 3381 | 3754 | 4149 |
| Sweden | 904 | 8.6 | 938 | 990 | 1,039 | 845 | 877 | 926 | 971 |
| | Source: ECIS - European Cancer Information System | | | | | | | | |
| | From https://ecis.jrc.ec.europa.eu/ | | | | | | | | |
| | (C) European union | | | | | | | | |

| | ECIS data | | | | | Fraction MZL, MCL, FL, DLBCL | | | 0.854 |
|---|---|---|---|---|---|---|---|---|---|
| | ALL Non-Hodgkin lymphoma | | | | | MZL, MCL, FL, DLBCL only | | | |
| Country | Cases 2022 | Crude rate | Cases 2025 | Cases 2030 | Cases 2035 | 2022 | 2025 | 2030 | 2035 |
| Austria | 1,600 | 17.8 | 1,667 | 1,773 | 1,881 | 1366 | 1424 | 1514 | 1606 |
| Belgium | 2,653 | 22.8 | 2,753 | 2,929 | 3,116 | 2266 | 2351 | 2501 | 2661 |
| Bulgaria | 492 | 7.2 | 489 | 488 | 492 | 420 | 418 | 417 | 420 |
| Croatia | 846 | 21.9 | 836 | 849 | 862 | 722 | 714 | 725 | 736 |
| Cyprus | 171 | 18.9 | 182 | 197 | 205 | 146 | 155 | 168 | 175 |
| Czechia | 1,765 | 16.8 | 1,820 | 1,894 | 1,963 | 1507 | 1554 | 1617 | 1676 |
| Denmark | 1,758 | 29.9 | 1,841 | 1,955 | 2,042 | 1501 | 1572 | 1670 | 1744 |
| EU-27 | 92,707 | 20.8 | 95,483 | 100,638 | 105,702 | 79172 | 81542 | 85945 | 90270 |
| Estonia | 255 | 19.1 | 265 | 272 | 284 | 218 | 226 | 232 | 243 |
| Finland | 1,502 | 27.1 | 1,567 | 1,657 | 1,724 | 1283 | 1338 | 1415 | 1472 |
| France | 16,972 | 25 | 17,692 | 18,965 | 20,202 | 14494 | 15109 | 16196 | 17253 |
| Germany | 18,980 | 22.8 | 19,513 | 20,219 | 20,915 | 16209 | 16664 | 17267 | 17861 |
| Greece | 1,663 | 15.9 | 1,672 | 1,730 | 1,785 | 1420 | 1428 | 1477 | 1524 |
| Hungary | 1,316 | 13.6 | 1,320 | 1,343 | 1,373 | 1124 | 1127 | 1147 | 1173 |
| Ireland | 1,028 | 20.3 | 1,110 | 1,242 | 1,387 | 878 | 948 | 1061 | 1184 |
| Italy | 15,576 | 26.4 | 15,795 | 16,579 | 17,281 | 13302 | 13489 | 14158 | 14758 |
| Latvia | 253 | 13.5 | 253 | 253 | 249 | 216 | 216 | 216 | 213 |
| Lithuania | 306 | 10.9 | 317 | 320 | 332 | 261 | 271 | 273 | 284 |
| Luxembourg | 93 | 14.4 | 99 | 110 | 126 | 79 | 85 | 94 | 108 |
| Malta | 131 | 25.1 | 139 | 154 | 172 | 112 | 119 | 132 | 147 |
| Netherlands | 4,381 | 24.9 | 4,589 | 4,904 | 5,161 | 3741 | 3919 | 4188 | 4407 |
| Poland | 4,169 | 11.1 | 4,293 | 4,559 | 4,830 | 3560 | 3666 | 3893 | 4125 |
| Portugal | 2,437 | 23.5 | 2,594 | 2,713 | 2,817 | 2081 | 2215 | 2317 | 2406 |
| Romania | 1,754 | 9.2 | 1,777 | 1,784 | 1,787 | 1498 | 1518 | 1524 | 1526 |
| Slovakia | 683 | 12.6 | 711 | 760 | 809 | 583 | 607 | 649 | 691 |
| Slovenia | 478 | 22.7 | 501 | 532 | 564 | 408 | 428 | 454 | 482 |
| Spain | 9,070 | 19.1 | 9,482 | 10,265 | 11,026 | 7746 | 8098 | 8766 | 9416 |
| Sweden | 2,375 | 22.7 | 2,475 | 2,641 | 2,777 | 2028 | 2114 | 2255 | 2372 |
| | Source: ECIS - European Cancer Information System | | | | | | | | |
| | From https://ecis.jrc.ec.europa.eu/ | | | | | | | | |
| | (C) European union | | | | | | | | |

## Table S18: Evolution of the estimated pool of AML patients eligible for radioligand therapy used as conditioning for allogeneic hematopoietic stem cell transplantation


1) Passweg et al. (2014) European Society for Blood and Marrow Transplantation EBMT. Hematopoietic SCT in Europe: data and trends in 2012 with special consideration of pediatric transplantation. Bone Marrow Transplant. 2014 Jun;49(6):744-50. doi: 10.1038/bmt.2014.55

2) Passweg et al. (2019) European Society for Blood and Marrow Transplantation (EBMT). The EBMT activity survey report 2017: a focus on allogeneic HCT for nonmalignant indications and on the use of non-HCT cell therapies. Bone Marrow Transplant. 2019 Oct;54(10):1575-1585. doi: 10.1038/s41409-019-0465-9

3) Khalidi e6t al. (1998) The immunophenotype of adult acute myeloid leukemia: high frequency of lymphoid antigen expression and comparison of immunophenotype, French-American-British classification, and karyotypic abnormalities. Am J Clin Pathol. 1998 Feb;109(2):211-20. doi: 10.1093/ajcp/109.2.211


| | | | | | | |
|---|---|---|---|---|---|---|
| stem cell transplantations in EU in 2012 (1) | 4784 | | | | | |
| stem cell transplantations in EU in 2017 (2) | 6676 | | | | | |
| cases extrapolated to 2022 (same rate) | 9316 | | | | | |
| of which with target expression 97,2% (3) | 9055 | 0.454 | calc. fraction of AML treatments with allogenic stem cell transplantation | Increase rate (>2022) | 0.03 | 3% |

| | AML and CLL only | | | | AML only (38%) target expression 97.2% | | | | Restricted to stem cell transplantation (first authorisation could be in 2025/26) | | | | stem cell transplantation + 3% p.a. | | | |
|---|---|---|---|---|---|---|---|---|---|---|---|---|---|---|---|---|
| Country | 2022 | 2025 | 2030 | 2035 | 2022 | 2025 | 2030 | 2035 | 2022 | 2025 | 2030 | 2035 | 2022 | 2025 | 2030 | 2035 |
| Austria | 893 | 932 | 993 | 1053 | 330 | 344 | 367 | 389 | 150 | 157 | 167 | 177 | 150 | 171 | 211 | 260 |
| Belgium | 1629 | 1687 | 1781 | 1878 | 602 | 623 | 658 | 694 | 273 | 283 | 299 | 315 | 273 | 309 | 379 | 463 |
| Bulgaria | 468 | 465 | 468 | 471 | 173 | 172 | 173 | 174 | 79 | 78 | 79 | 79 | 79 | 85 | 99 | 116 |
| Croatia | 506 | 502 | 515 | 535 | 187 | 185 | 190 | 198 | 85 | 84 | 87 | 90 | 85 | 92 | 110 | 132 |
| Cyprus | 133 | 141 | 157 | 173 | 49 | 52 | 58 | 64 | 22 | 24 | 26 | 29 | 22 | 26 | 33 | 43 |
| Czechia | 1219 | 1265 | 1341 | 1407 | 450 | 467 | 495 | 520 | 205 | 212 | 225 | 236 | 205 | 232 | 285 | 347 |
| Denmark | 855 | 896 | 957 | 1001 | 316 | 331 | 354 | 370 | 143 | 150 | 161 | 168 | 143 | 164 | 204 | 247 |
| EU-27 | 53941 | 55577 | 58816 | 62212 | 19924 | 20528 | 21724 | 22979 | 9055 | 9330 | 9874 | 10444 | 9055 | 10195 | 12508 | 15337 |
| Estonia | 170 | 175 | 183 | 191 | 63 | 65 | 68 | 71 | 29 | 29 | 31 | 32 | 29 | 32 | 39 | 47 |
| Finland | 598 | 624 | 660 | 691 | 221 | 230 | 244 | 255 | 100 | 105 | 111 | 116 | 100 | 114 | 140 | 170 |
| France | 9914 | 10310 | 11013 | 11713 | 3662 | 3808 | 4068 | 4326 | 1664 | 1731 | 1849 | 1966 | 1664 | 1891 | 2342 | 2887 |
| Germany | 11074 | 11403 | 11819 | 12268 | 4090 | 4212 | 4365 | 4531 | 1859 | 1914 | 1984 | 2059 | 1859 | 2092 | 2513 | 3024 |
| Greece | 1421 | 1410 | 1462 | 1530 | 525 | 521 | 540 | 565 | 238 | 237 | 245 | 257 | 238 | 259 | 311 | 377 |
| Hungary | 1017 | 1023 | 1053 | 1087 | 376 | 378 | 389 | 402 | 171 | 172 | 177 | 182 | 171 | 188 | 224 | 268 |
| Ireland | 416 | 446 | 494 | 547 | 154 | 165 | 182 | 202 | 70 | 75 | 83 | 92 | 70 | 82 | 105 | 135 |
| Italy | 7916 | 8022 | 8463 | 8946 | 2924 | 2963 | 3126 | 3304 | 1329 | 1347 | 1421 | 1502 | 1329 | 1472 | 1800 | 2205 |
| Latvia | 214 | 216 | 215 | 216 | 79 | 80 | 79 | 80 | 36 | 36 | 36 | 36 | 36 | 40 | 46 | 53 |
| Lithuania | 347 | 355 | 367 | 380 | 128 | 131 | 136 | 141 | 58 | 60 | 62 | 64 | 58 | 65 | 78 | 94 |
| Luxembou | 57 | 61 | 69 | 80 | 21 | 22 | 25 | 30 | 10 | 10 | 12 | 13 | 10 | 11 | 15 | 20 |
| Malta | 37 | 40 | 44 | 49 | 14 | 15 | 16 | 18 | 6 | 7 | 7 | 8 | 6 | 7 | 9 | 12 |
| Netherland | 2299 | 2407 | 2568 | 2699 | 849 | 889 | 948 | 997 | 386 | 404 | 431 | 453 | 386 | 442 | 546 | 665 |
| Poland | 3776 | 3905 | 4202 | 4512 | 1395 | 1442 | 1552 | 1667 | 634 | 655 | 705 | 757 | 634 | 716 | 894 | 1112 |
| Portugal | 1147 | 1234 | 1305 | 1379 | 424 | 456 | 482 | 509 | 193 | 207 | 219 | 231 | 193 | 226 | 278 | 340 |
| Romania | 1448 | 1462 | 1495 | 1535 | 535 | 540 | 552 | 567 | 243 | 245 | 251 | 258 | 243 | 268 | 318 | 378 |
| Slovakia | 512 | 532 | 575 | 614 | 189 | 197 | 212 | 227 | 86 | 89 | 96 | 103 | 86 | 98 | 122 | 151 |
| Slovenia | 295 | 312 | 339 | 367 | 109 | 115 | 125 | 136 | 50 | 52 | 57 | 62 | 50 | 57 | 72 | 91 |
| Spain | 4380 | 4558 | 4970 | 5426 | 1618 | 1683 | 1836 | 2004 | 735 | 765 | 834 | 911 | 735 | 836 | 1057 | 1338 |
| Sweden | 1199 | 1242 | 1321 | 1382 | 443 | 459 | 488 | 511 | 201 | 209 | 222 | 232 | 201 | 228 | 281 | 341 |

| | AML and CLL only | | | | AML only (38%) target expression 97.2% | | | | lower: Restricted to stem cell transplantation | | | | upper: stem cell transplantation + 3% p.a. | | | | |
|---|---|---|---|---|---|---|---|---|---|---|---|---|---|---|---|---|---|
| Country | 2022 | 2025 | 2030 | 2035 | 2022 | 2025 | 2030 | 2035 | 2022 | 2025 | 2030 | 2035 | 2022 | 2025 | 2030 | 2035 | Country |
| EU-27 | 53941 | 55577 | 58816 | 62212 | 19924 | 20528 | 21724 | 22979 | 9055 | 9330 | 9874 | 10444 | 9055 | 10195 | 12508 | 15337 | EU-27 |
| France | 9914 | 10310 | 11013 | 11713 | 3662 | 3808 | 4068 | 4326 | 1664 | 1731 | 1849 | 1966 | 1664 | 1891 | 2342 | 2887 | France |
| Germany | 11074 | 11403 | 11819 | 12268 | 4090 | 4212 | 4365 | 4531 | 1859 | 1914 | 1984 | 2059 | 1859 | 2092 | 2513 | 3024 | Germany |
| Italy | 7916 | 8022 | 8463 | 8946 | 2924 | 2963 | 3126 | 3304 | 1329 | 1347 | 1421 | 1502 | 1329 | 1472 | 1800 | 2205 | Italy |
| Spain | 4380 | 4558 | 4970 | 5426 | 1618 | 1683 | 1836 | 2004 | 735 | 765 | 834 | 911 | 735 | 836 | 1057 | 1338 | Spain |
| EU big 4 | | | | | | | | | | 5757 | 6088 | 6438 | | 6291 | 7712 | 9455 | EU big 4 |

## Table S19: Evolution of the estimated pool of patients eligible for radioligand treatment of Waldenström's Macroglobulinemia

1) García-Sanz and Tedeschi. The Management of Relapsed or Refractory Waldenström's Macroglobulinemia. Hematol Oncol Clin North Am. 2023 Aug;37(4):727-749. doi: 10.1016/j.hoc.2023.04.006

2) Chohan and Kapoor. BTK Inhibitors and Other Targeted Therapies in Waldenström Macroglobulinemia. Hemato. 2023; 4(2):135-157. doi.org/10.3390/hemato4020012

| | |
|---|---|
| incidence, min (1) | 0.0000036 |
| incidence max (1) | 0.0000055 |

**all cases considered eligible over time as there is no cure for LPL/WM (1,2)**

| | | | | | 2022 | | 2025 | | 2030 | | 2035 | |
|---|---|---|---|---|---|---|---|---|---|---|---|---|
| Country | Population 2022 | Population 2025 | Population 2030 | Population 2035 | min | max | min | max | min | max | min | max |
| Austria | 8,978,929 | 9,111,243 | 9,214,690 | 9,319,086 | 32 | 49 | 33 | 50 | 33 | 51 | 34 | 51 |
| Belgium | 11,617,623 | 11,829,411 | 12,009,045 | 12,179,830 | 42 | 64 | 43 | 65 | 43 | 66 | 44 | 67 |
| Bulgaria | 6,838,937 | 6,860,349 | 6,574,153 | 6,333,689 | 25 | 38 | 25 | 38 | 24 | 36 | 23 | 35 |
| Croatia | 3,862,305 | 3,810,628 | 3,693,206 | 3,593,292 | 14 | 21 | 14 | 21 | 13 | 20 | 13 | 20 |
| Cyprus | 904,705 | 941,765 | 957,744 | 967,207 | 3 | 5 | 3 | 5 | 3 | 5 | 3 | 5 |
| Czechia | 10,516,707 | 11,017,341 | 10,851,301 | 10,728,942 | 38 | 58 | 40 | 61 | 39 | 60 | 39 | 59 |
| Denmark | 5,873,420 | 5,979,924 | 6,059,699 | 6,112,281 | 21 | 32 | 22 | 33 | 22 | 33 | 22 | 34 |
| Estonia | 1,331,796 | 1,377,519 | 1,358,611 | 1,344,440 | 5 | 7 | 5 | 8 | 5 | 7 | 5 | 7 |
| EU-27 | 446,735,291 | 453,168,040 | 452,700,101 | 451,991,345 | 1608 | 2457 | 1631 | 2492 | 1630 | 2490 | 1627 | 2486 |
| Finland | 5,548,241 | 5,640,423 | 5,631,487 | 5,601,455 | 20 | 31 | 20 | 31 | 20 | 31 | 20 | 31 |
| France | 67,871,925 | 68,658,223 | 69,386,211 | 70,026,306 | 244 | 373 | 247 | 378 | 250 | 382 | 252 | 385 |
| Germany | 83,237,124 | 85,207,514 | 85,284,256 | 85,216,229 | 300 | 458 | 307 | 469 | 307 | 469 | 307 | 469 |
| Greece | 10,459,782 | 10,320,364 | 10,032,545 | 9,758,893 | 38 | 58 | 37 | 57 | 36 | 55 | 35 | 54 |
| Hungary | 9,689,010 | 9,644,847 | 9,526,758 | 9,422,235 | 35 | 53 | 35 | 53 | 34 | 52 | 34 | 52 |
| Ireland | 5,060,004 | 5,257,383 | 5,416,927 | 5,579,300 | 18 | 28 | 19 | 29 | 20 | 30 | 20 | 31 |
| Italy | 59,030,133 | 58,951,070 | 58,773,783 | 58,655,761 | 213 | 325 | 212 | 324 | 212 | 323 | 211 | 323 |
| Latvia | 1,875,757 | 1,863,089 | 1,756,334 | 1,660,761 | 7 | 10 | 7 | 10 | 6 | 10 | 6 | 9 |
| Lithuania | 2,805,998 | 2,860,472 | 2,741,927 | 2,622,099 | 10 | 15 | 10 | 16 | 10 | 15 | 9 | 14 |
| Luxembourg | 645,397 | 687,081 | 740,420 | 788,408 | 2 | 4 | 2 | 4 | 3 | 4 | 3 | 4 |
| Malta | 520,971 | 553,623 | 604,727 | 649,012 | 2 | 3 | 2 | 3 | 2 | 3 | 2 | 4 |
| Netherlands | 17,590,672 | 18,048,588 | 18,341,701 | 18,564,556 | 63 | 97 | 65 | 99 | 66 | 101 | 67 | 102 |
| Poland | 37,654,247 | 38,381,332 | 37,420,524 | 36,517,358 | 136 | 207 | 138 | 211 | 135 | 206 | 131 | 201 |
| Portugal | 10,352,042 | 10,372,141 | 10,249,138 | 10,120,798 | 37 | 57 | 37 | 57 | 37 | 56 | 36 | 56 |
| Romania | 19,042,455 | 18,831,698 | 18,218,553 | 17,683,694 | 69 | 105 | 68 | 104 | 66 | 100 | 64 | 97 |
| Slovakia | 5,434,712 | 5,521,368 | 5,450,183 | 5,368,574 | 20 | 30 | 20 | 30 | 20 | 30 | 19 | 30 |
| Slovenia | 2,107,180 | 2,120,770 | 2,118,806 | 2,113,672 | 8 | 12 | 8 | 12 | 8 | 12 | 8 | 12 |
| Spain | 47,432,893 | 48,614,060 | 49,266,930 | 49,760,920 | 171 | 261 | 175 | 267 | 177 | 271 | 179 | 274 |
| Sweden | 10,452,326 | 10,705,814 | 11,020,442 | 11,302,547 | 38 | 57 | 39 | 59 | 40 | 61 | 41 | 62 |

| | | | | | 2022 | | 2025 | | 2030 | | 2035 | | |
|---|---|---|---|---|---|---|---|---|---|---|---|---|---|
| Country | Population 2022 | Population 2025 | Population 2030 | Population 2035 | min | max | min | max | min | max | min | max | Country |
| EU-27 | 446,735,291 | 453,168,040 | 452,700,101 | 451,991,345 | 1608 | 2457 | 1631 | 2492 | 1630 | 2490 | 1627 | 2486 | EU-27 |
| France | 67,871,925 | 68,658,223 | 69,386,211 | 70,026,306 | 244 | 373 | 247 | 378 | 250 | 382 | 252 | 385 | France |
| Germany | 83,237,124 | 85,207,514 | 85,284,256 | 85,216,229 | 300 | 458 | 307 | 469 | 307 | 469 | 307 | 469 | Germany |
| Italy | 59,030,133 | 58,951,070 | 58,773,783 | 58,655,761 | 213 | 325 | 212 | 324 | 212 | 323 | 211 | 323 | Italy |
| Spain | 47,432,893 | 48,614,060 | 49,266,930 | 49,760,920 | 171 | 261 | 175 | 267 | 177 | 271 | 179 | 274 | Spain |
| EU big 4 | | | | | | | | | 946 | 1445 | 949 | 1450 | EU big 4 |

## Table S20: Estimate of the pool of Multiple Myeloma patients eligible for radioligand therapy after at least five prior treatment regimes.


1) Kanas et al. (2021) Estimate of multiple myeloma patients by line of therapy in the USA: population-level projections 2020-2025. Future Oncol. 2021 Mar;17(8):921-930. doi: 10.2217/fon-2020-0970

2) Daniele et al. (2022) Response rates and minimal residual disease outcomes as potential surrogates for progression-free survival in newly diagnosed multiple myeloma. PLoS One. 2022 May 12;17(5):e0267979. doi: 10.1371/journal.pone.0267979


| Min | Max |
|---|---|
| 0.005 | 0.02 |

6th line treatment 0.5% only (1) | acumulating effect may be 2% (1,2) | authorisation within 2030 uncertain

Fraction of multiple myeloma: 0.935

| | ALL Myeloma | | | | | Multiple myeloma only | | | | lower bound | | | | upper bound | | | |
|---|---|---|---|---|---|---|---|---|---|---|---|---|---|---|---|---|---|
| Country | Cases 2022 | Crude rate | Cases 2025 | Cases 2030 | Cases 2035 | 2022 | 2025 | 2030 | 2035 | 2022 | 2025 | 2030 | 2035 | 2022 | 2025 | 2030 | 2035 |
| Austria | 538 | 6 | 563 | 609 | 654 | 503 | 526 | 569 | 611 | 3 | 3 | 3 | 3 | 10 | 11 | 11 | 12 |
| Belgium | 1,013 | 8.7 | 1052 | 1,125 | 1,198 | 947 | 984 | 1052 | 1120 | 5 | 5 | 5 | 6 | 19 | 20 | 21 | 22 |
| Bulgaria | 208 | 3 | 209 | 209 | 212 | 194 | 195 | 195 | 198 | 1 | 1 | 1 | 1 | 4 | 4 | 4 | 4 |
| Croatia | 400 | 10.4 | 400 | 415 | 423 | 374 | 374 | 388 | 396 | 2 | 2 | 2 | 2 | 7 | 7 | 8 | 8 |
| Cyprus | 78 | 8.6 | 81 | 91 | 103 | 73 | 76 | 85 | 96 | 0 | 0 | 0 | 0 | 1 | 2 | 2 | 2 |
| Czechia | 624 | 5.9 | 647 | 671 | 695 | 583 | 605 | 627 | 650 | 3 | 3 | 3 | 3 | 12 | 12 | 13 | 13 |
| Denmark | 661 | 11.3 | 691 | 733 | 766 | 618 | 646 | 685 | 716 | 3 | 3 | 3 | 4 | 12 | 13 | 14 | 14 |
| EU-27 | 35,333 | 7.9 | 36556 | 38,938 | 41,264 | 33036 | 34180 | 36407 | 38582 | 165 | 171 | 182 | 193 | 661 | 684 | 728 | 772 |
| Estonia | 112 | 8.4 | 113 | 122 | 126 | 105 | 106 | 114 | 118 | 1 | 1 | 1 | 1 | 2 | 2 | 2 | 2 |
| Finland | 466 | 8.4 | 492 | 521 | 545 | 436 | 460 | 487 | 510 | 2 | 2 | 2 | 3 | 9 | 9 | 10 | 10 |
| France | 5,425 | 8 | 5695 | 6,112 | 6,441 | 5072 | 5325 | 5715 | 6022 | 25 | 27 | 29 | 30 | 101 | 106 | 114 | 120 |
| Germany | 6,932 | 8.3 | 7108 | 7,408 | 7,764 | 6481 | 6646 | 6926 | 7259 | 32 | 33 | 35 | 36 | 130 | 133 | 139 | 145 |
| Greece | 763 | 7.3 | 766 | 805 | 844 | 713 | 716 | 753 | 789 | 4 | 4 | 4 | 4 | 14 | 14 | 15 | 16 |
| Hungary | 404 | 4.2 | 407 | 416 | 433 | 378 | 381 | 389 | 405 | 2 | 2 | 2 | 2 | 8 | 8 | 8 | 8 |
| Ireland | 438 | 8.7 | 476 | 538 | 606 | 410 | 445 | 503 | 567 | 2 | 2 | 3 | 3 | 8 | 9 | 10 | 11 |
| Italy | 6,298 | 10.7 | 6443 | 6,873 | 7,294 | 5889 | 6024 | 6426 | 6820 | 29 | 30 | 32 | 34 | 118 | 120 | 129 | 136 |
| Latvia | 119 | 6.3 | 120 | 122 | 122 | 111 | 112 | 114 | 114 | 1 | 1 | 1 | 1 | 2 | 2 | 2 | 2 |
| Lithuania | 179 | 6.4 | 186 | 191 | 199 | 167 | 174 | 179 | 186 | 1 | 1 | 1 | 1 | 3 | 3 | 4 | 4 |
| Luxembourg | 38 | 5.9 | 38 | 47 | 53 | 36 | 36 | 44 | 50 | 0 | 0 | 0 | 0 | 1 | 1 | 1 | 1 |
| Malta | 32 | 6.1 | 33 | 39 | 45 | 30 | 31 | 36 | 42 | 0 | 0 | 0 | 0 | 1 | 1 | 1 | 1 |
| Netherlands | 1,607 | 9.1 | 1691 | 1,817 | 1,916 | 1503 | 1581 | 1699 | 1791 | 8 | 8 | 8 | 9 | 30 | 32 | 34 | 36 |
| Poland | 2,433 | 6.5 | 2507 | 2,672 | 2,844 | 2275 | 2344 | 2498 | 2659 | 11 | 12 | 12 | 13 | 45 | 47 | 50 | 53 |
| Portugal | 994 | 9.6 | 1080 | 1,149 | 1,217 | 929 | 1010 | 1074 | 1138 | 5 | 5 | 5 | 6 | 19 | 20 | 21 | 23 |
| Romania | 725 | 3.8 | 738 | 757 | 771 | 678 | 690 | 708 | 721 | 3 | 3 | 4 | 4 | 14 | 14 | 14 | 14 |
| Slovakia | 337 | 6.2 | 351 | 383 | 415 | 315 | 328 | 358 | 388 | 2 | 2 | 2 | 2 | 6 | 7 | 7 | 8 |
| Slovenia | 171 | 8.1 | 180 | 193 | 210 | 160 | 168 | 180 | 196 | 1 | 1 | 1 | 1 | 3 | 3 | 4 | 4 |
| Spain | 3,434 | 7.2 | 3616 | 4,015 | 4,437 | 3211 | 3381 | 3754 | 4149 | 16 | 17 | 19 | 21 | 64 | 68 | 75 | 83 |
| Sweden | 904 | 8.6 | 938 | 990 | 1,039 | 845 | 877 | 926 | 971 | 4 | 4 | 5 | 5 | 17 | 18 | 19 | 19 |

**Upper bound not considered - assumption to extend treatment to patients with short period between subsequent relapses is too speculative**

Fraction of multiple myeloma: 0.935

| | ALL Myeloma | | | | | Multiple myeloma only | | | | lower bound | | | | upper bound | | | |
|---|---|---|---|---|---|---|---|---|---|---|---|---|---|---|---|---|---|
| Country | Cases 2022 | Crude rate | Cases 2025 | Cases 2030 | Cases 2035 | 2022 | 2025 | 2030 | 2035 | 2022 | 2025 | 2030 | 2035 | 2022 | 2025 | 2030 | 2035 |
| EU-27 | 35,333 | 7.9 | 36556 | 38,938 | 41,264 | 33036 | 34180 | 36407 | 38582 | | | | | | | | |
| France | 5,425 | 8 | 5695 | 6,112 | 6,441 | 5072 | 5325 | 5715 | 6022 | 25 | 27 | 29 | 30 | 101 | 106 | 114 | 120 |
| Germany | 6,932 | 8.3 | 7108 | 7,408 | 7,764 | 6481 | 6646 | 6926 | 7259 | 32 | 33 | 35 | 36 | 130 | 133 | 139 | 145 |
| Italy | 6,298 | 10.7 | 6443 | 6,873 | 7,294 | 5889 | 6024 | 6426 | 6820 | 29 | 30 | 32 | 34 | 118 | 120 | 129 | 136 |
| Spain | 3,434 | 7.2 | 3616 | 4,015 | 4,437 | 3211 | 3381 | 3754 | 4149 | 16 | 17 | 19 | 21 | 64 | 68 | 75 | 83 |
| EU big 4 | | | | | | 20653 | 21376 | 22821 | 24250 | 103 | 107 | 114 | 121 | 413 | 428 | 456 | 485 |

# Table S21: Estimate of the pool of patients eligible for radioligand therapy treating clear cell Renal Cell Carcinoma (ccRCC)

| Parameters relevant for the caluclation of eligible patients | reported range | |
|---|---|---|
| fraction of ccRCC of all kidney cancers (1,2) | 59.5% | 72.8% |
| overall fraction of patients with metastattic disease at the time of first diagnosis (3) | 13.0% | 13.0% |
| patients considered at risk of metastatic progression after complete or partial nephrectomy (4) | 50.0% | 50.0% |
| fraction of patients at risk of metastatic progression after nephrectomy (3,4): 50% of (100%-13%) = | 43.5% | 43.5% |
| Fraction of patients to be treated to prevent OR control metastatic disease (43.5% + 13%) | 56.5% | 56.5% |
| Limitation due to toxicity (7) - 6/14 eligible for 2 cycles (5 responders), 1/6 for 3 cycles | 10.0% | 20.0% |
| prevalence of CA IX expression (5,6) | 91.7% | 97.0% |

| | |
|---|---|
| upper range estimate | 7.98% |
| lower range estimate | 3.08% |


1) Motzer et al. (2022) NCCN Clinical Practice Guidelines in Oncology. J Natl Compr Canc Netw. 2022 Jan;20(1):71-90. doi: 10.6004/jnccn.2022.0001 (59.5%)
Shuch et al. (2015) Understanding pathologic variants of renal cell carcinoma: distilling therapeutic opportunities from biologic complexity. Eur Urol. 2015 Jan;67(1):85-97. doi: 10.1016/j.eururo.2014.04.029 (63.8%)
Lipworth et al. (2016) Renal cell cancer histological subtype distribution differs by race and sex. BJU Int. 2016 Feb;117(2):260-5. doi: 10.1111/bju.12950 (64.5%)
Chow WH, Dong LM, Devesa SS. Epidemiology and risk factors for kidney cancer. Nat Rev Urol. 2010 May;7(5):245-57. doi: 10.1038/nrurol.2010.46 (67.9%)
2) Gansler et al. (2018) Trends in reporting histological subtyping of renal cell carcinoma: association with cancer center type. Hum Pathol. 2018 Apr;74:99-108. doi: 10.1016/j.humpath.2018.01.010 (72.8%)
3) Monda et al. (2013) The Metastatic Risk of Renal Cell Carcinoma by Primary Tumor Size and Subtype. Eur Urol Open Sci. 2023 May 10;52:137-144. doi: 10.1016/j.euros.2023.04.015
4) Bandini et al. (2018) Effect of pathological high-risk features on cancer-specific mortality in non-metastatic clear cell renal cell carcinoma: a tool for optimizing patient selection for adjuvant therapy. World J Urol. 2018 Jan;36(1):51-57. doi: 10.1007/s00345-017-2093-6
5) Soyupak et al. (2005) CA9 expression as a prognostic factor in renal clear cell carcinoma. Urol Int. 2005;74(1):68-73. doi: 10.1159/000082713
6) Tostain et al. (2010) Carbonic anhydrase 9 in clear cell renal cell carcinoma: a marker for diagnosis, prognosis and treatment. Eur J Cancer. 2010 Dec;46(18):3141-8. doi: 10.1016/j.ejca.2010.07.020
7) Muselaers et al. (2016) Phase 2 Study of Lutetium 177–Labeled Anti–Carbonic Anhydrase IX Monoclonal Antibody Girentuximab in Patients with Advanced Renal Cell Carcinoma. European Urology 2016, 69: 767-770 http://dx.doi.org/10.1016/j.eururo.2015.11.033


| | | | | | | authorisation expected for 2031 - thus only to consider for 2035 estimate | | | | | | | |
|---|---|---|---|---|---|---|---|---|---|---|---|---|---|
| | All cases kidney cancer | | | | | lower estimate | | | | upper estimate | | | |
| Country | Cases 2022 | Crude rate | Cases 2025 | Cases 2030 | Cases 2035 | Cases 2022 | Cases 2025 | Cases 2030 | Cases 2035 | Cases 2022 | Cases 2025 | Cases 2030 | Cases 2035 |
| Austria | 1,427 | 15.9 | 1,451 | 1,546 | 1,628 | 44 | 45 | 48 | 50 | 114 | 116 | 123 | 130 |
| Belgium | 1,924 | 16.6 | 2,318 | 2,443 | 2,549 | 59 | 71 | 75 | 79 | 154 | 185 | 195 | 203 |
| Bulgaria | 790 | 11.6 | 847 | 845 | 844 | 24 | 26 | 26 | 26 | 63 | 68 | 67 | 67 |
| Croatia | 871 | 22.6 | 900 | 918 | 930 | 27 | 28 | 28 | 29 | 70 | 72 | 73 | 74 |
| Cyprus | 79 | 8.7 | 105 | 114 | 120 | 2 | 3 | 4 | 4 | 6 | 8 | 9 | 10 |
| Czechia | 3,087 | 29.4 | 3,560 | 3,788 | 3,979 | 95 | 110 | 117 | 123 | 246 | 284 | 302 | 318 |
| Denmark | 1,260 | 21.5 | 1,196 | 1,251 | 1,288 | 39 | 37 | 39 | 40 | 101 | 95 | 100 | 103 |
| EU-27 | 90,572 | 20.3 | 92,171 | 96,912 | 101,062 | 2792 | 2841 | 2988 | 3115 | 7227 | 7355 | 7733 | 8064 |
| Estonia | 303 | 22.8 | 416 | 438 | 461 | 9 | 13 | 14 | 14 | 24 | 33 | 35 | 37 |
| Finland | 1,118 | 20.2 | 1,141 | 1,200 | 1,249 | 34 | 35 | 37 | 39 | 89 | 91 | 96 | 100 |
| France | 14,541 | 21.4 | 15,791 | 16,692 | 17,482 | 448 | 487 | 515 | 539 | 1160 | 1260 | 1332 | 1395 |
| Germany | 20,514 | 24.6 | 17,797 | 18,413 | 18,929 | 632 | 549 | 568 | 584 | 1637 | 1420 | 1469 | 1510 |
| Greece | 1,875 | 17.9 | 1,886 | 1,951 | 2,018 | 58 | 58 | 60 | 62 | 150 | 150 | 156 | 161 |
| Hungary | 2,006 | 20.7 | 2,123 | 2,192 | 2,264 | 62 | 65 | 68 | 70 | 160 | 169 | 175 | 181 |
| Ireland | 883 | 17.5 | 1,195 | 1,339 | 1,479 | 27 | 37 | 41 | 46 | 70 | 95 | 107 | 118 |
| Italy | 13,666 | 23.2 | 13,065 | 13,718 | 14,276 | 421 | 403 | 423 | 440 | 1090 | 1043 | 1095 | 1139 |
| Latvia | 531 | 28.3 | 539 | 541 | 538 | 16 | 17 | 17 | 17 | 42 | 43 | 43 | 43 |
| Lithuania | 642 | 22.9 | 864 | 880 | 886 | 20 | 27 | 27 | 27 | 51 | 69 | 70 | 71 |
| Luxembourg | 74 | 11.5 | 69 | 78 | 89 | 2 | 2 | 2 | 3 | 6 | 6 | 6 | 7 |
| Malta | 92 | 17.7 | 88 | 97 | 106 | 3 | 3 | 3 | 3 | 7 | 7 | 8 | 8 |
| Netherlands | 2,978 | 16.9 | 3,284 | 3,495 | 3,652 | 92 | 101 | 108 | 113 | 238 | 262 | 279 | 291 |
| Poland | 5,664 | 15 | 6,502 | 6,903 | 7,284 | 175 | 200 | 213 | 225 | 452 | 519 | 551 | 581 |
| Portugal | 1,624 | 15.7 | 1,268 | 1,324 | 1,381 | 50 | 39 | 41 | 43 | 130 | 101 | 106 | 110 |
| Romania | 2,649 | 13.9 | 2,783 | 2,833 | 2,865 | 82 | 86 | 87 | 88 | 211 | 222 | 226 | 229 |
| Slovakia | 1,183 | 21.8 | 1,455 | 1,560 | 1,652 | 36 | 45 | 48 | 51 | 94 | 116 | 124 | 132 |
| Slovenia | 387 | 18.4 | 506 | 537 | 565 | 12 | 16 | 17 | 17 | 31 | 40 | 43 | 45 |
| Spain | 8,686 | 18.3 | 9,454 | 10,167 | 10,822 | 268 | 291 | 313 | 334 | 693 | 754 | 811 | 864 |
| Sweden | 1,718 | 16.4 | 1,568 | 1,649 | 1,726 | 53 | 48 | 51 | 53 | 137 | 125 | 132 | 138 |

Source: ECIS - European Cancer Information System
From https://ecis.jrc.ec.europa.eu/
(C) European union

| | All cases kidney cancer | | | | | lower estimate | | | | upper estimate | | | | |
|---|---|---|---|---|---|---|---|---|---|---|---|---|---|---|
| Country | Cases 2022 | Crude rate | Cases 2025 | Cases 2030 | Cases 2035 | Cases 2022 | Cases 2025 | Cases 2030 | Cases 2035 | Cases 2022 | Cases 2025 | Cases 2030 | Cases 2035 | Country |
| EU-27 | 90,572 | 20.3 | 92,171 | 96,912 | 101,062 | 2792 | 2841 | 2988 | 3115 | 7227 | 7355 | 7733 | 8064 | EU-27 |
| France | 14,541 | 21.4 | 15,791 | 16,692 | 17,482 | 448 | 487 | 515 | 539 | 1160 | 1260 | 1332 | 1395 | France |
| Germany | 20,514 | 24.6 | 17,797 | 18,413 | 18,929 | 632 | 549 | 568 | 584 | 1637 | 1420 | 1469 | 1510 | Germany |
| Italy | 13,666 | 23.2 | 13,065 | 13,718 | 14,276 | 421 | 403 | 423 | 440 | 1090 | 1043 | 1095 | 1139 | Italy |
| Spain | 8,686 | 18.3 | 9,454 | 10,167 | 10,822 | 268 | 291 | 313 | 334 | 693 | 754 | 811 | 864 | Spain |
| EU big 4 | | | | | | | | | 1896 | | | | 4908 | EU big 4 |

# Table S22: Estimate of the pool of B7-H3+ patients eligible for neuroblastoma treatment with radioligand therapy

| Parameters considered in the estimate | range | | | |
|---|---|---|---|---|
| Incidence rate for neuroblastoma (1,2) | 0.00000012 | 0.000001 | | |
| prevalence of B7-H3 expression (3) | 0.82 | 0.82 | for lower range estimate | 9.84E-08 |
| fraction of patients with inoperable tumours | 1 | 1 | for upper range estimate | 0.00000082 |


1) Darlix et al. (2017) Epidemiology for primary brain tumors: a nationwide population-based study. J Neurooncol. 2017 Feb;131(3):525-546. doi: 10.1007/s11060-016-2318-3

2) Gatta et al. (2017) RARECAREnet working group. Burden and centralised treatment in Europe of rare tumours: results of RARECAREnet-a population-based study. Lancet Oncol. 2017 Aug;18(8):1022-1039. doi: 10.1016/S1470-2045(17)30445-X

3) Majzner et al. (2019) CAR T Cells Targeting B7-H3, a Pan-Cancer Antigen, Demonstrate Potent Preclinical Activity Against Pediatric Solid Tumors and Brain Tumors. Clin Cancer Res. 2019 Apr 15;25(8):2560-2574. doi: 10.1158/1078-0432.CCR-18-0432


| | | | | | in spite of new target and drawbacks - childhood cancer - without adequate therapy! | | | | | | | |
|---|---|---|---|---|---|---|---|---|---|---|---|---|
| | Population 2022 and projections | | | | lower range estimate | | | | upper range estimate | | | |
| Country | 2022 | 2025 | 2030 | 2035 | 2022 | 2025 | 2030 | 2035 | 2022 | 2025 | 2030 | 2035 |
| Austria | 8,978,929 | 9,111,243 | 9,214,690 | 9,319,086 | 1 | 1 | 1 | 1 | 7 | 7 | 8 | 8 |
| Belgium | 11,617,623 | 11,829,411 | 12,009,045 | 12,179,830 | 1 | 1 | 1 | 1 | 10 | 10 | 10 | 10 |
| Bulgaria | 6,838,937 | 6,860,349 | 6,574,153 | 6,333,689 | 1 | 1 | 1 | 1 | 6 | 6 | 5 | 5 |
| Croatia | 3,862,305 | 3,810,628 | 3,693,206 | 3,593,292 | 0 | 0 | 0 | 0 | 3 | 3 | 3 | 3 |
| Cyprus | 904,705 | 941,765 | 957,744 | 967,207 | 0 | 0 | 0 | 0 | 1 | 1 | 1 | 1 |
| Czechia | 10,516,707 | 11,017,341 | 10,851,301 | 10,728,942 | 1 | 1 | 1 | 1 | 9 | 9 | 9 | 9 |
| Denmark | 5,873,420 | 5,979,924 | 6,059,699 | 6,112,281 | 1 | 1 | 1 | 1 | 5 | 5 | 5 | 5 |
| Estonia | 1,331,796 | 1,377,519 | 1,358,611 | 1,344,440 | 0 | 0 | 0 | 0 | 1 | 1 | 1 | 1 |
| EU-27 | 446,735,291 | 453,168,040 | 452,700,101 | 451,991,345 | 44 | 45 | 45 | 44 | 366 | 372 | 371 | 371 |
| Finland | 5,548,241 | 5,640,423 | 5,631,487 | 5,601,455 | 1 | 1 | 1 | 1 | 5 | 5 | 5 | 5 |
| France | 67,871,925 | 68,658,223 | 69,386,211 | 70,026,306 | 7 | 7 | 7 | 7 | 56 | 56 | 57 | 57 |
| Germany | 83,237,124 | 85,207,514 | 85,284,256 | 85,216,229 | 8 | 8 | 8 | 8 | 68 | 70 | 70 | 70 |
| Greece | 10,459,782 | 10,320,364 | 10,032,545 | 9,758,893 | 1 | 1 | 1 | 1 | 9 | 8 | 8 | 8 |
| Hungary | 9,689,010 | 9,644,847 | 9,526,758 | 9,422,235 | 1 | 1 | 1 | 1 | 8 | 8 | 8 | 8 |
| Ireland | 5,060,004 | 5,257,383 | 5,416,927 | 5,579,300 | 0 | 1 | 1 | 1 | 4 | 4 | 4 | 5 |
| Italy | 59,030,133 | 58,951,070 | 58,773,783 | 58,655,761 | 6 | 6 | 6 | 6 | 48 | 48 | 48 | 48 |
| Latvia | 1,875,757 | 1,863,089 | 1,756,334 | 1,660,761 | 0 | 0 | 0 | 0 | 2 | 2 | 1 | 1 |
| Lithuania | 2,805,998 | 2,860,472 | 2,741,927 | 2,622,099 | 0 | 0 | 0 | 0 | 2 | 2 | 2 | 2 |
| Luxembourg | 645,397 | 687,081 | 740,420 | 788,408 | 0 | 0 | 0 | 0 | 1 | 1 | 1 | 1 |
| Malta | 520,971 | 553,623 | 604,727 | 649,012 | 0 | 0 | 0 | 0 | 0 | 0 | 0 | 1 |
| Netherlands | 17,590,672 | 18,048,588 | 18,341,701 | 18,564,556 | 2 | 2 | 2 | 2 | 14 | 15 | 15 | 15 |
| Poland | 37,654,247 | 38,381,332 | 37,420,524 | 36,517,358 | 4 | 4 | 4 | 4 | 31 | 31 | 31 | 30 |
| Portugal | 10,352,042 | 10,372,141 | 10,249,138 | 10,120,798 | 1 | 1 | 1 | 1 | 8 | 9 | 8 | 8 |
| Romania | 19,042,455 | 18,831,698 | 18,218,553 | 17,683,694 | 2 | 2 | 2 | 2 | 16 | 15 | 15 | 15 |
| Slovakia | 5,434,712 | 5,521,368 | 5,450,183 | 5,368,574 | 1 | 1 | 1 | 1 | 4 | 5 | 4 | 4 |
| Slovenia | 2,107,180 | 2,120,770 | 2,118,806 | 2,113,672 | 0 | 0 | 0 | 0 | 2 | 2 | 2 | 2 |
| Spain | 47,432,893 | 48,614,060 | 49,266,930 | 49,760,920 | 5 | 5 | 5 | 5 | 39 | 40 | 40 | 41 |
| Sweden | 10,452,326 | 10,705,814 | 11,020,442 | 11,302,547 | 1 | 1 | 1 | 1 | 9 | 9 | 9 | 9 |

Source: ECIS - European Cancer Information System
From https://ecis.jrc.ec.europa.eu/
(C) European union

| | Population 2022 and projections | | | | lower range estimate | | | | upper range estimate | | | |
|---|---|---|---|---|---|---|---|---|---|---|---|---|
| Country | 2022 | 2025 | 2030 | 2035 | 2022 | 2025 | 2030 | 2035 | 2022 | 2025 | 2030 | 2035 |
| EU-27 | 446,735,291 | 453,168,040 | 452,700,101 | 451,991,345 | 44 | 45 | 45 | 44 | 366 | 372 | 371 | 371 |
| France | 67,871,925 | 68,658,223 | 69,386,211 | 70,026,306 | 7 | 7 | 7 | 7 | 56 | 56 | 57 | 57 |
| Germany | 83,237,124 | 85,207,514 | 85,284,256 | 85,216,229 | 8 | 8 | 8 | 8 | 68 | 70 | 70 | 70 |
| Italy | 59,030,133 | 58,951,070 | 58,773,783 | 58,655,761 | 6 | 6 | 6 | 6 | 48 | 48 | 48 | 48 |
| Spain | 47,432,893 | 48,614,060 | 49,266,930 | 49,760,920 | 5 | 5 | 5 | 5 | 39 | 40 | 40 | 41 |
| EU big 4 | | | | | | 26 | 26 | 26 | | 214 | 215 | 216 |

## Table S23: Estimate of the pool of sst2+ malignant meningioma patients eligible for radioligand therapy and summary for brain cancer indications

| Parameters considered in the estimate | range | | | |
|---|---|---|---|---|
| Incidence rate for malignant meningioma (1,2) | 0.00000054 | 0.0000036 | for lower range estimate | 3.456E-07 |
| prevalence of somatostatin receptor expression (3) | 0.64 | 1 | for upper range estimate | 0.0000036 |

1) Wöhrer et al. (2009) The Austrian Brain Tumour Registry: a cooperative way to establish a population-based brain tumour registry. J Neurooncol. 2009 Dec;95(3):401-411. doi: 10.1007/s11060-009-9938-9

2) Darlix et al. (2017) Epidemiology for primary brain tumors: a nationwide population-based study. J Neurooncol. 2017 Feb;131(3):525-546. doi: 10.1007/s11060-016-2318-3

3) Wu et al. (2020) Clinical Significance of Somatostatin Receptor (SSTR) 2 in Meningioma. Front Oncol. 2020 Sep 3;10:1633. doi: 10.3389/fonc.2020.01633

| | | | | | **malignant meningioma** - off-label use of Lutathera could be authorised fast, before 2030 | | | | | | | | **Neuroblastoma**, target B7-H3 | | | | **malignant menigioma & neuroblastoma** | | | |
|---|---|---|---|---|---|---|---|---|---|---|---|---|---|---|---|---|---|---|---|---|
| | Population 2022 and projections | | | | lower range estimate | | | | upper range estimate | | | | lower estimate | | upper estimate | | lower estimate | | upper estimate | |
| Country | 2022 | 2025 | 2030 | 2035 | 2022 | 2025 | 2030 | 2035 | 2022 | 2025 | 2030 | 2035 | 2030 | 2035 | 2030 | 2035 | 2030 | 2035 | 2030 | 2035 |
| Austria | 8,978,929 | 9,111,243 | 9,214,690 | 9,319,086 | 3 | 3 | 3 | 3 | 32 | 33 | 33 | 34 | 1 | 1 | 8 | 8 | 4 | 4 | 41 | 41 |
| Belgium | 11,617,623 | 11,829,411 | 12,009,045 | 12,179,830 | 4 | 4 | 4 | 4 | 42 | 43 | 43 | 44 | 1 | 1 | 10 | 10 | 5 | 5 | 53 | 54 |
| Bulgaria | 6,838,937 | 6,860,349 | 6,574,153 | 6,333,689 | 2 | 2 | 2 | 2 | 25 | 25 | 24 | 23 | 1 | 1 | 5 | 5 | 3 | 3 | 29 | 28 |
| Croatia | 3,862,305 | 3,810,628 | 3,693,206 | 3,593,292 | 1 | 1 | 1 | 1 | 14 | 14 | 13 | 13 | 0 | 0 | 3 | 3 | 2 | 2 | 16 | 16 |
| Cyprus | 904,705 | 941,765 | 957,744 | 967,207 | 0 | 0 | 0 | 0 | 3 | 3 | 3 | 3 | 0 | 0 | 1 | 1 | 0 | 0 | 4 | 4 |
| Czechia | 10,516,707 | 11,017,341 | 10,851,301 | 10,728,942 | 4 | 4 | 4 | 4 | 38 | 40 | 39 | 39 | 1 | 1 | 9 | 9 | 5 | 5 | 48 | 47 |
| Denmark | 5,873,420 | 5,979,924 | 6,059,699 | 6,112,281 | 2 | 2 | 2 | 2 | 21 | 22 | 22 | 22 | 1 | 1 | 5 | 5 | 3 | 3 | 27 | 27 |
| Estonia | 1,331,796 | 1,377,519 | 1,358,611 | 1,344,440 | 0 | 0 | 0 | 0 | 5 | 5 | 5 | 5 | 0 | 0 | 1 | 1 | 1 | 1 | 6 | 6 |
| EU-27 | 446,735,291 | 453,168,040 | 452,700,101 | 451,991,345 | 154 | 157 | 156 | 156 | 1608 | 1631 | 1630 | 1627 | 45 | 44 | 371 | 371 | 201 | 201 | 2001 | 1998 |
| Finland | 5,548,241 | 5,640,423 | 5,631,487 | 5,601,455 | 2 | 2 | 2 | 2 | 20 | 20 | 20 | 20 | 1 | 1 | 5 | 5 | 3 | 2 | 25 | 25 |
| France | 67,871,925 | 68,658,223 | 69,386,211 | 70,026,306 | 23 | 24 | 24 | 24 | 244 | 247 | 250 | 252 | 7 | 7 | 57 | 57 | 31 | 31 | 307 | 310 |
| Germany | 83,237,124 | 85,207,514 | 85,284,256 | 85,216,229 | 29 | 29 | 29 | 29 | 300 | 307 | 307 | 307 | 8 | 8 | 70 | 70 | 38 | 38 | 377 | 377 |
| Greece | 10,459,782 | 10,320,364 | 10,032,545 | 9,758,893 | 4 | 4 | 3 | 3 | 38 | 37 | 36 | 35 | 1 | 1 | 8 | 8 | 4 | 4 | 44 | 43 |
| Hungary | 9,689,010 | 9,644,847 | 9,526,758 | 9,422,235 | 3 | 3 | 3 | 3 | 35 | 35 | 34 | 34 | 1 | 1 | 8 | 8 | 4 | 4 | 42 | 42 |
| Ireland | 5,060,004 | 5,257,383 | 5,416,927 | 5,579,300 | 2 | 2 | 2 | 2 | 18 | 19 | 20 | 20 | 1 | 1 | 4 | 5 | 2 | 2 | 24 | 25 |
| Italy | 59,030,133 | 58,951,070 | 58,773,783 | 58,655,761 | 20 | 20 | 20 | 20 | 213 | 212 | 212 | 211 | 6 | 6 | 48 | 48 | 26 | 26 | 260 | 259 |
| Latvia | 1,875,757 | 1,863,089 | 1,756,334 | 1,660,761 | 1 | 1 | 1 | 1 | 7 | 7 | 6 | 6 | 0 | 0 | 1 | 1 | 1 | 1 | 8 | 7 |
| Lithuania | 2,805,998 | 2,860,472 | 2,741,927 | 2,622,099 | 1 | 1 | 1 | 1 | 10 | 10 | 10 | 9 | 0 | 0 | 2 | 2 | 1 | 1 | 12 | 12 |
| Luxembourg | 645,397 | 687,081 | 740,420 | 788,408 | 0 | 0 | 0 | 0 | 2 | 2 | 3 | 3 | 0 | 0 | 1 | 1 | 0 | 0 | 3 | 3 |
| Malta | 520,971 | 553,623 | 604,727 | 649,012 | 0 | 0 | 0 | 0 | 2 | 2 | 2 | 2 | 0 | 0 | 0 | 1 | 0 | 0 | 3 | 3 |
| Netherlands | 17,590,672 | 18,048,588 | 18,341,701 | 18,564,556 | 6 | 6 | 6 | 6 | 63 | 65 | 66 | 67 | 2 | 2 | 15 | 15 | 8 | 8 | 81 | 82 |
| Poland | 37,654,247 | 38,381,332 | 37,420,524 | 36,517,358 | 13 | 13 | 13 | 13 | 136 | 138 | 135 | 131 | 4 | 4 | 31 | 30 | 17 | 16 | 165 | 161 |
| Portugal | 10,352,042 | 10,372,141 | 10,249,138 | 10,120,798 | 4 | 4 | 4 | 3 | 37 | 37 | 37 | 36 | 1 | 1 | 8 | 8 | 5 | 4 | 45 | 45 |
| Romania | 19,042,455 | 18,831,698 | 18,218,553 | 17,683,694 | 7 | 7 | 6 | 6 | 69 | 68 | 66 | 64 | 2 | 2 | 15 | 15 | 8 | 8 | 81 | 78 |
| Slovakia | 5,434,712 | 5,521,368 | 5,450,183 | 5,368,574 | 2 | 2 | 2 | 2 | 20 | 20 | 20 | 19 | 1 | 1 | 4 | 4 | 2 | 2 | 24 | 24 |
| Slovenia | 2,107,180 | 2,120,770 | 2,118,806 | 2,113,672 | 1 | 1 | 1 | 1 | 8 | 8 | 8 | 8 | 0 | 0 | 2 | 2 | 1 | 1 | 9 | 9 |
| Spain | 47,432,893 | 48,614,060 | 49,266,930 | 49,760,920 | 16 | 17 | 17 | 17 | 171 | 175 | 177 | 179 | 5 | 5 | 40 | 41 | 22 | 22 | 218 | 220 |
| Sweden | 10,452,326 | 10,705,814 | 11,020,442 | 11,302,547 | 4 | 4 | 4 | 4 | 38 | 39 | 40 | 41 | 1 | 1 | 9 | 9 | 5 | 5 | 49 | 50 |

Source: ECIS - European Cancer Information System
From https://ecis.jrc.ec.europa.eu/
(C) European union

| | | | | | | | | | | | | | | | | | **malignant menigioma & neuroblastoma** | | | |
|---|---|---|---|---|---|---|---|---|---|---|---|---|---|---|---|---|---|---|---|---|
| | Population 2022 and projections | | | | lower range estimate | | | | upper range estimate | | | | lower estimate | | upper estimate | | lower estimate | | upper estimate | |
| Country | 2022 | 2025 | 2030 | 2035 | 2022 | 2025 | 2030 | 2025 | 2022 | 2025 | 2030 | 2025 | 2030 | 2035 | 2030 | 2035 | 2030 | 2035 | 2030 | 2035 |
| EU-27 | 446,735,291 | 453,168,040 | 452,700,101 | 451,991,345 | 154 | 157 | 156 | 156 | 1608 | 1631 | 1630 | 1627 | 45 | 44 | 371 | 371 | 201 | 201 | 2001 | 1998 |
| France | 67,871,925 | 68,658,223 | 69,386,211 | 70,026,306 | 23 | 24 | 24 | 24 | 244 | 247 | 250 | 252 | 7 | 7 | 57 | 57 | 31 | 31 | 307 | 310 |
| Germany | 83,237,124 | 85,207,514 | 85,284,256 | 85,216,229 | 29 | 29 | 29 | 29 | 300 | 307 | 307 | 307 | 8 | 8 | 70 | 70 | 38 | 38 | 377 | 377 |
| Italy | 59,030,133 | 58,951,070 | 58,773,783 | 58,655,761 | 20 | 20 | 20 | 20 | 213 | 212 | 212 | 211 | 6 | 6 | 48 | 48 | 26 | 26 | 260 | 259 |
| Spain | 47,432,893 | 48,614,060 | 49,266,930 | 49,760,920 | 16 | 17 | 17 | 17 | 171 | 175 | 177 | 179 | 5 | 5 | 40 | 41 | 22 | 22 | 218 | 220 |
| EU big 4 | 257,572,075 | 261,430,867 | 262,711,180 | 263,659,216 | | | | | | | | | | | | | 117 | 117 | 1161 | 1165 |

**Figure S7: Incidence-derived estimate of the maximum patient pool biologically and clinically eligible for radioligand therapy in the EU-27 for authorised indications for $^{177}$Lu-DOTATATE and $^{177}$Lu-PSMA-617, potential authorisation expansions to earlier disease stages, and potential new authorisations of radioligand therapies in phase 2 or 3 clinical trials. The columns represent the lower and upper bound of the sensitivity analysis.**

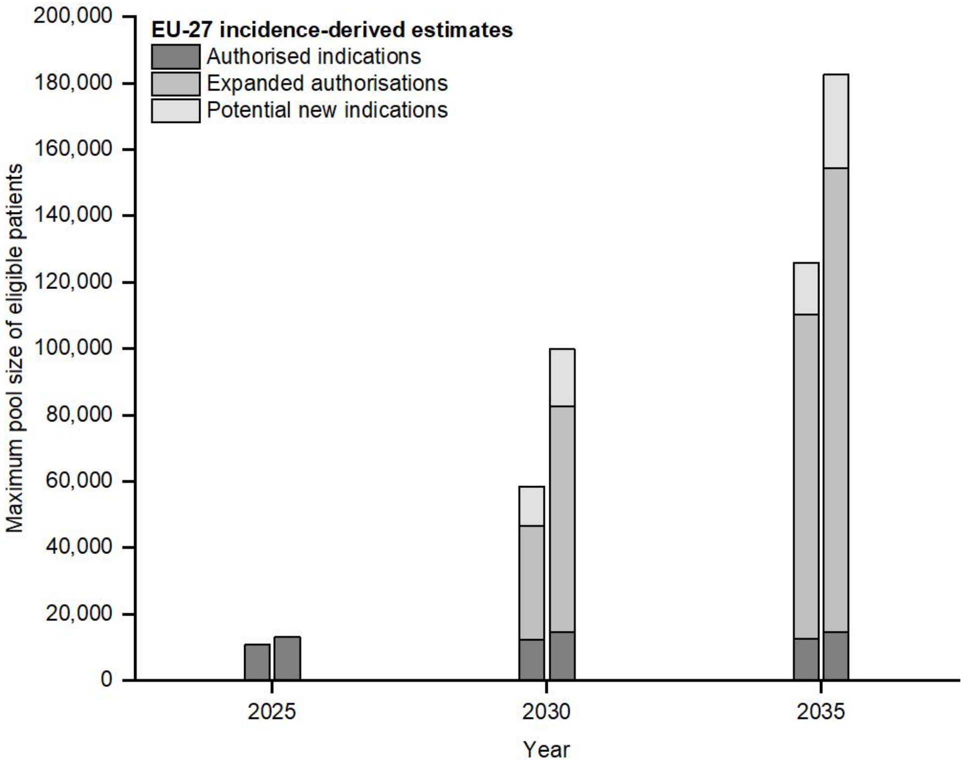

**Table S24: Scenario for the uptake of RLTs (authorised 2017 and later) in the healthcare systems of the EU-4 (Grermany, France, Italy and Spain), which have well-developed nuclear medicine infrastructure.[a]**

| | | *Eligible patient pool EU-4 - Germany, France, Italy, Spain* | | | | | | *Uptake scenario EU-4 - Germany, France, Italy, Spain* | | | | | |
|---|---|---|---|---|---|---|---|---|---|---|---|---|---|
| | | *Prevalence-derived* | | *Incidence-derived* | | | | *Prevalence-derived* | | *Incidence-derived* | | | |
| | | **2030** | **2033** | **2030** | | **2035** | | **2030** | **2033** | **2030** | | **2035** | |
| | | Midpoint values | | Min | Max | Min | Max | Midpoint values | | Min | Max | Min | Max |
| ***Haematological malignancies*** | AML | 43,800 | 45,500 | 6,100 | 7,700 | 6,400 | 9,500 | 0 | 0 | 0 | 0 | 0 | 0 |
| | LPL/WM | 2,600 | 2,600 | 900 | 1,400 | 900 | 1,500 | 1,040 | 1,040 | 360 | 560 | 360 | 600 |
| | MM | 500 | 500 | 100 | 100 | 100 | 100 | 200 | 200 | 40 | 40 | 40 | 40 |
| ***Kidney cancer*** | mccRCC | – | 3,200 | – | – | 1,900 | 4,900 | – | 1,300 | – | – | 760 | 1,960 |
| ***Neuroendocrine tumors*** | NET | 33,300 | 33,400 | 10,000 | 10,700 | 10,100 | 10,800 | 13,320 | 13,360 | 4,000 | 4,280 | 4,040 | 4,320 |
| ***Prostate cancer*** | mPC | – | – | 17,300 | 37,900 | 55,300 | 80,900 | – | – | 7,785 | 17,055 | 24,885 | 36,405 |
| | mCRPC | 11,700 | 12,500 | – | – | – | – | 5,265 | 5,625 | – | – | – | – |
| | OMPC | 6,000 | 6,400 | – | – | – | – | 0 | 0 | – | – | – | – |
| | mHSPC | 24,200 | 25,700 | – | – | – | – | 10,890 | 11,565 | – | – | – | – |
| ***Brain and CNS tumors*** | | 9,500 | 9,500 | 100 | 1,200 | 100 | 1,200 | 3,800 | 3,800 | 40 | 480 | 40 | 480 |
| **Total** | | 131,600 | 139,300 | 34,500 | 59,000 | 74,800 | 108,900 | 34,515 | 37,190 | 12,225 | 22,415 | 30,125 | 43,805 |
| **Mean** | | | | 46,750 | | 91,850 | | | | 17,320 | | 36,965 | |
| **Percentage of patients treated from the eligible pool** | | | | | | | | **26%** | **27%** | **37%** | | **40%** | |

[a]Estimates are based on the pool of patients eligible for RLT and on eligible-to-treated patient ratios derived from Table 5. For prostate cancer, 45% of the eligible patients were assumed to be treated; for all other indications, 40% was used. For AML, no uptake was assumed due to the hypothesis that competition with immune therapies may hinder demonstrating superiority, and for oligometastatic prostate cancer, it was assumed that less expensive and resource demanding methods might be prioritised.

AML: acute myeloid leukemia; mccRCC: metastatic clear cell renal cell carcinoma; mCRPC: metastatic castration resistant prostate cancer; mHSPC: metastatic hormone sensitive prostate cancer; LPL/WM: lymphoplasmacytic lymphoma/Waldenström Macroglobulinemia; MM: multiple myeloma; NET: neuroendocrine tumor; mPC, metastatic prostate cancer; OMPC: oligometastatic prostate cancer; RLT, radioligand therapy.

**Table S25: Patients treated with $^{177}$Lu radioligand therapies in 2020 as derived from radionuclide utilisation data in Table S3[a] and hypothetical breakdown of the uptake scenario[b] to individual EU countries. It is questionable whether countries with zero or low utilisation in 2020 may achieve the number of treatments forecasted for 2030 and 2035. Incidence-derived data are presented for all EU-27 countries and prevalence-derived data are presented for the EU-4 (Germany, France, Italy, and Spain) and UK (marked with (*); for these data, the last column refers to 2033). Numbers are rounded to the nearest hundred.**

| Country | Patients treated in 2020 and uptake scenario forecast for 2030 and 2035 | | |
|---|---|---|---|
| | 2020 | 2030 | 2035 |
| **Austria** | 500 | 400 – 700 | 1,000 – 1,400 |
| **Belgium** | 40 | 600 – 1,200 | 1,600 – 2,300 |
| **Bulgaria** | 0 | 200 – 400 | 500 – 700 |
| **Croatia** | 0 | 400 – 800 | 1,300 – 1,800 |
| **Cyprus** | 0 | < 100 | ≈ 100 |
| **Czechia** | 40 | 500 – 900 | 1,200 – 1,700 |
| **Denmark** | No data | 300 – 600 | 700 – 1,100 |
| **Estonia** | 10 | ≈ 100 | 200 – 300 |
| **Finland** | 10 | 300 – 600 | 800 – 1,200 |
| **France** | 400 | 3,500 – 6,400 | 8,500 – 12,300 |
| **France (*)** | 400 | 6,700 | 7,400 |
| **Germany** | 2,500 | 4,000 – 7,400 | 10,000 – 14,500 |
| **Germany (*)** | 2,500 | 17,000 | 18,400 |
| **Greece** | 10 | 400 – 800 | 1,100 – 1,500 |
| **Hungary** | 0 | 400 – 500 | 1,000 – 1,400 |
| **Ireland** | 0 | 300 – 500) | (700 – 1,000 |
| **Italy** | 1,000 | 2,500 – 4,500 | 6,100 – 8,800 |
| **Italy (*)** | 1,000 | 6,100 | 6,900 |
| **Latvia** | 0 | 100 – 200 | 400 – 700 |
| **Lithuania** | 0 | 200 – 300 | 400 – 700 |
| **Luxembourg** | 0 | < 100 | ≈ 100 |
| **Malta** | 0 | < 100 | ≈ 100 |
| **Netherlands** | 200 | 800 – 1,400 | 1,900 – 2,700 |
| **Poland** | 100 | 1,500 – 2,700 | 3,500 – 5,100 |
| **Portugal** | 10 | 500 – 900 | 1,500 – 1,700 |
| **Romania** | 0 | 700 – 1,200 | 1,600 – 2,300 |
| **Slovakia** | 30 | 200 – 400 | 600 – 900 |
| **Slovenia** | 0 | 100 – 200 | 300 – 400 |
| **Spain** | 100 | 2,200 – 4,100 | 5,600 – 8,100 |
| **Spain (*)** | 100 | 17,000 | 18,400 |
| **Sweden** | 100 | 600 – 1,200 | 1,600 – 2,400 |
| **UK (*)** | 600 | 6,900 | 7,300 |
| **EU-27** | **5,100** | **20,900 – 37,700** | **50,900 – 73,700** |
| **EU-4** | 4,100 | 12,000 – 22,400 | 30,100 – 43,800 |
| **EU-4 (*)** | 4,100 | 34,500 | 37,200 |

[a] An average of 4 procedures was assumed per patient treated with $^{177}$Lu-DOTATATE and $^{177}$Lu-PSMA.

[c] The criteria outlined in the discussion and Table S24 were applied to all EU countries: Estimates are based on the pool of patients eligible for RLT. For prostate cancer, 45% of the eligible patients were assumed to be treated; for all other indications, 40% was used. For AML, no uptake was assumed due to the hypothesis that competition with immune therapies may hinder demonstrating superiority.

### Table S26: IQVIA estimate of RLT treatment capacity in Germany, France, Italy, and Spain in 2023.[1]

| Country | Available treatment slots in 2023 per 100.000 | Population (ECIS, 2022) | Available treatment slots in 2023 | Number of patients assuming an average of 4 doses (1 dose = 1 treatment cycle) per patient |
|---|---|---|---|---|
| **Germany** | 55–60 | 83,166,711 | 45,742–49,900 | 11,436–12,475 |
| **France** | 25–30 | 67,320,216 | 16,830–20,196 | 4,208–5,049 |
| **Italy** | 5–10 | 59,641,488 | 2,982–5,964 | 746–1,491 |
| **Spain** | 75–80 | 47,332,614 | 35,499–37,866 | 8,875–9,467 |

[1] IQVIA. Succeeding with Innovation: The State of Radioligand Therapy Readiness in Europe. 2023. https://www.iqvia.com/insights/the-iqvia-institute/reports-and-publications/reports/succeeding-with-innovation-state-of-radioligand-therapy (accessed 31 January

## Radionuclide production and availability

Most of the radiopharmaceuticals considered in the present work will be radiolabelled with $^{177}$Lu (177-Lutetium). Products radiolabelled with the alpha emitters $^{225}$Ac (225-Actinium) and $^{212}$Pb (212 Lead) will have to overcome the hurdle of first marketing authorisation, which is not expected before 2030, and there is only one phase 2 trial using $^{212}$Pb (cf. Table S2). The following considerations will focus on $^{177}$Lu, $^{225}$Ac and $^{212}$Pb and the assumptions made in the uptake scenario in Table S24 are applied to the incidence-derived pool size in Table 1 in order to estimate the radionuclide demand for the whole EU.

The production capacity for $^{177}$Lu has recently been estimated by Giammarile et al. (2024).[1] With the currently used 6 reactors (2023) a global annual production capacity of 204 kCi (7,500 TBq) has been calculated, which is supposed to be upscaled by 2032 to 612 kCi (22,600 TBq) involving 9 reactors.[1] Using the numbers of the patient pool of Table 1 with a fraction of 45% of the prostate cancer patients really treated and 40% of the patients with other indications, and an average of four treatment cycles with 7.4 GBq each, the required total activity at bed side in 2035 can be calculated as about 1,500 - 2,100 TBq. If 50% is lost during production and distribution, the EU-27 may consume 3,000 - 4,000 TBq, which roughly equals to 15-20% of the predicted production capacity in 2032. Considering the slowly expanding access to nuclear medical treatments in large parts of the world,[2] it can be assumed that there will be no supply shortages in Europe under the condition that all reactors will work as scheduled.

Zimmermann recently described the production methods and production sites for $^{225}$Ac as well as the evolution of the landscape until 2032.[3] Zimmermann estimated a production capacity by 2032 of "largely above 25 TBq (670 Ci)" per year, which is sufficient for "at least 2 million patent doses per year".[3] This is an expected increase by a factor of 250 compared to the current availability of 0.1 TBq (2023), accompanied by significant diversification of production methods and suppliers.[3] The same author reviewed the supply of $^{212}$Pb and reported an availability of more than 10,000 patient doses in 2025 with an up-scaling by a factor of 10 until 2030.[4]

Alpha-emitting radiopharmaceuticals will most likely be used for at maximum one half of non-responders to beta-therapy.[5] For an upper estimate of the $^{225}$Ac demand, we apply the same uptake scenario and assume a typical dose of 10 MBq of $^{225}$Ac administered four times per patient. With these assumptions, the maximum annual demand for $^{225}$Ac at bed may not exceed 1-1.5 TBq. Thus, considering a loss of 50% of the $^{225}$Ac during radiopharmaceutical production and transport, the EU-27 demand will amount to 2-3 TBq, equal to around 10% of the global production capacity forecasted for 2032.[3] Since in our assessment marketing authorisations are unlikely before 2030 supply shortages are not expected provided production will be up-scaled as planned.[3]

Currently the only $^{212}$Pb-labelled product in a phase 2 clinical trial is $^{212}$Pb-DOTAMTATE for the treatment of neuroendocrine tumours. The clinical trial does not require prior $^{177}$Lu-therapy as for the $^{225}$Ac radiopharmaceuticals in development. Thus, its market introduction might reduce the demand for $^{177}$Lu. If we generously assume in our uptake scenario that one third of the patients with neuroendocrine tumours might be treated from 2031 on (cf. Table S2) with 4 cycles of $^{212}$Pb-DOTAMTATE instead of $^{177}$Lu-DOTATATE, less than 10,000 doses would be required, i.e. less than 10% of the expected availability by 2030.

In summary, the production and supply with radionuclides should not be a bottleneck for the evolution of RLT if the production capacity of existing and new research reactors is upscaled as planned and the various industry activities on radionuclide production continue as planned.[1,3,4]

**References**


1. Giammarile F, Paez D, Zimmermann R, et al. Production and regulatory issues for theranostics. *Lancet Oncol.* 2024, **25**(6): e260-e269.
2. Abdel-Wahab M, Giammarile F, Carrara M, et al. Radiotherapy and theranostics: a Lancet Oncology Commission. *Lancet Oncol* 2024; **25**(11): e545-e80.
3. Zimmermann R. Is Actinium Really Happening? *J Nucl Med.* 2023; **64**(10):1516-1518.
4. Zimmermann R. Is $^{212}$Pb Really Happening? The Post-$^{177}$Lu/$^{225}$Ac Blockbuster? *J Nucl Med.* 2024; **65**(2):176-177.
5. Czernin J, Bodei L, Modlin I, Calais J. Reflections on the Demand for PSMA- and SSTR-Targeted Radiopharmaceutical Therapies: Why We Were Wrong (and Why We Will Be Right Eventually). *J Nucl Med* 2025; **66**(3): 333-6.

**Challenges of staffing and training for RLT**

The administration of radiopharmaceuticals requires a multidisciplinary range of expertise starting from radiochemists and radiopharmacists who prepare an injectable radioactive solution if it is not commercially available as a ready-to-inject product. Physicians and medical physicists must be trained in the handling of open radioactive substances; nursing staff must be able to guide patients in following instructions and manage emergencies. Medical physicists and technical assistants must be proficient in operating imaging systems—from calibration and functional testing to image acquisition and reconstruction—and must perform patient dosimetry. Radiation protection personnel must perform dose rate measurements, dispose of radioactive waste, carry out all procedures in accordance with the hospital's nuclear operating license requirements, and handle reporting. A detailed compilation of roles and responsibilities is available in a report of the International Atomic Energy Agency (IAEA).[1]

A translation of patient numbers in staffing requirements is based on algorithms and models that assign a time effort to a certain task for specific healthcare professionals.[1] Such models have recently been reviewed in the report "Analysis on workforce availability, education and training needs for the quality and safety of medical applications involving ionising radiation in the EU" of the EU-funded EU-REST project.[2] The survey-based study "European Union Radiation, Education, Staffing & Training (EU-REST)" aimed to collect and analyse workforce availability, education, and training of radiologists, nuclear medicine physicians, radiation oncologists, medical physicists, radiographers, and radiation therapists.[2]

The EU-REST report and the IAEA document specify the time effort for a nuclear medicine physician of 6 hours for a single RLT treatment cycle (p.78).[1,2] This estimate reflects primarily direct, patient-related procedural activities including patient education and consent, treatment administration, and follow-up care. In routine clinical practice, however, nuclear medicine physicians are additionally engaged in a wide spectrum of tasks such as interdisciplinary case discussions (e.g., tumour boards), supervision and training of junior staff, and participation in clinical research, continuing medical education, and administrative responsibilities. When these additional duties are taken into account, the effective physician capacity is substantially reduced. Taking into account factors such as annual leave, sick leave and absences for training, human resource departments do consider an average work time per year of 1640 hours as one full time equivalent (FTE).[1]

Four treatment cycles are authorised for $^{177}$Lu-DOTATATE treatment of neuroendocrine tumours and up to six for $^{177}$Lu-PSMA-617 treatment of prostate cancer. If we assume an average of 4 treatment cycles per patient, the number of expected patients treated in the EU-4 countries (Germany, France, Italy, Spain; cf. Table S24) by 2030 (17,000 – 35,000) and by 2035 (37,000) translate into 250-510 and 540 additionally required nuclear medicine physician FTEs, respectively. From the number of 3,865 nuclear medicine physicians (p.202) currently working in the EU-4 given in the EU-REST report we can derive a needed increase of up to 14% until 2030/35.[2] However, it must also be noted that a significant number of currently practicing nuclear medicine specialists do not yet have sufficient experience in performing RLT. The report specifies 6,116 nuclear medicine physicians for the whole EU-27 (p.202).[2] An increase of 14% translates into around 850 additional nuclear medicine physicians (FTE) needed within 2030/35 for the EU-27.

The European Guidelines on Medical Physics Experts say in Annex 2 that 10 complex radionuclide therapy procedures require 0.1 FTE of a medical physicist, and 100 procedures of SPECT/CT or PET/CT would require further 0.02 FTE and 0.05 FTE, respectively.[3] If we assume for our uptake scenario for the EU-4 countries in Table S24 one PET/CT for patient selection and one SPECT/CT for dosimetry and dose delivery control (only for the first treatment cycle) and an average of four treatment cycles per patient, the additional demand for medical physics services will amount to 700-1,400 additional medical physics staff until 2030, slightly increasing to 1,500 in 2035, which correspond to an increase of up to 25% of the medical physicists in the EU-4 (p.202). With currently 9,259 medical physicists working in the EU-27 (p.202) for the whole EU up to about 2,300 additional medical physicists will be required until 2035.

Radiation protection services are frequently also provided by medical physics services and not separately dealt with in the EU-REST report. The additional effort is related to the authorisation and documentation of patient release after RLT, the decontamination of administration rooms after therapy and its preparation for the next patient as well as with radioactive waste handling (which can be done in batches) and dosimetry of professionally exposed staff. If we assume an effort of one hour per patient and treatment cycle, this will amount to 90 FTE for the EU-4 and up to 150 for the whole EU (1 FTE = 1640 hours/year).

The time effort for nursing staff has not been assessed in the EU-REST project. The IAEA report "A model to assess staffing needs in nuclear medicine" specifies for $^{177}$Lu-PSMA therapies and for $^{177}$Lu-DOTATATE therapy a time effort for nurses of 25 hours and 38 hours, respectively. The 38 hours for neuroendocrine tumours are based on a hospitalisation of three days. While hospitalisation of RLT patients may be required in some cases, in an outpatient scheme the face-to-face availability of a nurse should not exceed 8 hours including some time for

patient preparation and preparation for patient release. A time effort of 8 hours per RLT treatment cycle yields additional 1,000 to 1,400 nurses that might be required for the EU-27 until 2035, most likely more depending on the local hospitalisation requirements.

RLTs require a diagnostic PET/CT scan before therapy to quantitatively assess receptor positivity and to select the patients which may benefit from RLT. The short-lived radionuclides used for this purpose ($^{18}F$ or $^{68}Ga$) will in most cases necessitate the preparation of the diagnostic radiopharmaceutical in the local hospital radiopharmacy. This will require the availability of a radiopharmacists or radiochemist and a pharmaceutical technologist. Whether additional workforce is needed for therapy depends on whether commercially available products can be used or not. Larger batch sizes of commercially produced radioligand therapies lead to staff savings by scale effects. Thus, the number of additional radiopharmacists and pharmaceutical technologist is difficult to predict and depends on the number of patients that can be treated locally with a single batch of the radiopharmaceutical.

**Table S27: Additional staffing requirements for nuclear medicine healthcare professionals based on the RLT uptake scenario (see Table S24) applied to EU-27 incidence derived data in Table 1.**

| Healthcare profession | Estimated additional number required | | remarks |
|---|---|---|---|
| | 2030 | 2035 | |
| Nuclear medicine physicians | ≈ 300 - 550 | ≈ 750 – 1,100 | |
| Medical physicists (including radiation protection) | ≈ 900 – 1,600 | ≈ 2,200 – 3,200 | Possible efficiency gains by validated single time point dosimetry procedures |
| Specialised nurses | ≥ 400 - 700 | ≥ 1,000 – 1,400 | Numbers based on outpatient schemes, effort much higher than 8h per single administration when hospitalisation required |
| Radiopharmacists and radiochemists | Ensure ≥ 2 for each treatment centre | | Assuming short-lived diagnostic radiopharmaceuticals produced locally, large batches commercial therapeutic radiopharmaceuticals distributed by industry |

The numbers derived above and compiled in Table S27 give a rough idea on the additional staffing needs in line with the uptake scenario as presented in Table S24. To ensure that efficient, safe and high-quality services are provided, education and training programmes are needed to create and maintain appropriate levels of knowledge and know-how. For this purpose, a greater standardization of professional qualifications is needed.[2]

Education and training of medical staff to the required levels will require coordinated efforts to provide consistent training and to scale up the training landscape.[4,5] The EU-REST report finds that already the definition of nuclear medicine as a separate specialty varies across the EU27.[2] Nevertheless, the training of nuclear medicine physicians appears "somehow harmonised" but in some countries a certification in radiation protection is not required.[2] The training and requirements for medical physicists and medical physics experts are very heterogeneous; still some countries have no education/training for medical physicists is in place.[2,6] A focus should be achieving a harmonised certification processes across countries to ensure consistency in the qualification of MPEs enabling them to fulfil their legal roles assigned by the Council Directive 2013/59/Euratom.[6-8] Guidelines on training schemes and curricula for medical physicists and medical physics experts have been elaborated by the by the European Federation of Organisations for Medical Physic and the European Commission.[3,4,7,8]

Thus, for each professional group, harmonisation of training across all 27 EU Member States (in terms of duration, curriculum, and certification of successful completion, central registration) is desirable.[2] This would benefit interchangeability of qualifications across Member States, facilitate mobility of relevant professionals, and foster high and homogeneous quality standards.[2] Professional organisations in nuclear medicine (EANM), medical physics (EFOMP) and the European Union (e.g. by PIANOFORTE in radiation protection (https://cordis.europa.eu/project/id/101061037 )) will not be able to shoulder these efforts alone; supporting adjustments of university curricula will be necessary.[8] In order to achieve truly harmonised standards for training, education and certification the EU-REST report suggests that EU Member States should rather adopt the recommendations of the EU-REST project than adapt to them (p.20).[2,6] However, this will depend on "the capacity of Member States to address a number of structural, regulatory and resource-related challenges".[6]

The EU-REST project also recommends that Member States establish and maintain registries for professionals in nuclear medicine. This would greatly facilitate the work of the European Human Resources Observatory for the Nuclear Sector (EHRO-N) managed by the European Commission's Joint Research Centre (JRC),[10] with the objective, among others, to review demand and supply of human resources and competences in the European nuclear sector and to identify gaps and deficiencies in education and training infrastructures. This activity covers the whole nuclear sector which is relevant to nuclear medicine to ensure sustainable radionuclide supply from

reactors, accelerators and where appropriate extracted from nuclear waste as well as training in radiation protection and waste management.

**Referenes:**


1. International Atomic Energy Agency. Human Health Reports No. 19: A Model to assess staffing needs in nuclear medicine. International Atomic Energy Agency, Vienna, 2022. https://www-pub.iaea.org/MTCD/Publications/PDF/PUB1965_web.pdf (accessed 30 March 2026)
2. European Commission, Directorate-General for Health and Food Safety, Directorate-General for Energy. 2025. Analysis on workforce availability, education and training needs for the quality and safety of medical applications involving ionising radiation in the EU. https://op.europa.eu/en/publication-detail/-/publication/dc0dd10d-d879-11ef-be2a-01aa75ed71a1/language-en (accessed 30 March 2026)
3. European Commission, Radiation Protection No 174, European Guidelines on Medical Physics Experts https://www.efomp.org/uploads/rp_174_full.pdf (accessed 30 March 2026)
4. Pascual TNB, Paez D, Iagaru A, et al. Guiding principles on the education and practice of theranostics. *Eur J Nucl Med Mol Imaging* 2024; **51**(8): 2320-31.
5. Bugani V, Battistelli L, Sansovini M, et al. Radioligand therapies in cancer: mapping the educational landscape in Europe. *Eur J Nucl Med Mol Imaging* 2023; **50**(9): 2692-8.
6. Garibaldi C, Sánchez RM, Visvikis D, et al. Analysis on workforce availability, education and training needs for medical physics experts to ensure quality and safety of medical applications involving ionising radiation in the EU - Status and recommendations from the EU-REST project. *Phys Med.* 2026; **142**:10572
7. EC. COUNCIL DIRECTIVE 2013/59/EURATOM of 5 December 2013 laying down basic safety standards for protection against the dangers arising from exposure to ionising radiation, and repealing Directives 89/618/Euratom, 90/641/Euratom, 96/29/Euratom, 97/43/Euratom and 2003/122/Euratom. https://eur-lex.europa.eu/LexUriServ/LexUriServ.do?uri=OJ:L:2014:013:0001:0073:EN:PDF (accessed 30 March 2026)
8. Caruana CJ, Christofides S, Hartmann GH. European Federation of Organisations for Medical Physics (EFOMP) policy statement 12.1: Recommendations on medical physics education and training in Europe 2014. *Phys Med* 2014; **30**(6): 598-603.
9. European Commission. CORDIS - Partnership for european research in radiation protection and detection of ionising radiation: towards a safer use and improved protection of the environment and human health. 2026. https://cordis.europa.eu/project/id/101061037 (accessed 31 January 2026).
10. European Commission, Joint Research Centre. ERHO-N European Human Resources Observatory for the Nuclear Sector (ERHO-N). https://joint-research-centre.ec.europa.eu/ehro-n_en (accessed 31 March 2026).
11. Michailidou E, Martin Ramos M, Galy J, et al. Nuclear education and training activities of the Joint Research Centre of the European Commission: Maintaining and enhancing nuclear skills and competences. *Nuclear Engineering and Design* 2024; **423**:113087
